# Improving selectivity of DNA–RNA binding zinc finger using directed evolution

**DOI:** 10.1186/s13104-019-4833-8

**Published:** 2019-12-04

**Authors:** Agata A. Sulej

**Affiliations:** Laboratory of Bioinformatics and Protein Engineering, International Institute of Molecular and Cell Biology, Ks. Trojdena 4, 02-109 Warsaw, Poland

**Keywords:** Directed evolution, Protein engineering, Zinc finger, Phage display, DNA–RNA hybrids

## Abstract

**Objective:**

Type C2H2 zinc fingers bind a variety of substrates, specific sequences in the double-stranded DNA counting among them. Engineering efforts led to the discovery of a set of general rules that enable obtaining zinc fingers modules that bind to almost any given sequence. The objective of this work was to determine an analogical set of rules for the binding of specific sequences in DNA–RNA hybrids using directed evolution of ZfQQR zinc finger. The target regions for evolution included the amino acid residues that directly interact with the substrate and linkers between the zinc finger modules.

**Results:**

The directed evolution was performed using selection based on biopanning of phage-displayed libraries of randomized regions in the ZfQQR zinc finger. The applied strategy of randomization of the middle module of the zinc finger along with input library bias and materials used for biopanning hindered the selection of the modules with altered specificity. However, the directed evolution of the linker sequence between modules enabled selection of variants with improved selectivity towards DNA–RNA hybrids in the presence of double-stranded DNA in comparison to the original ZfQQR. This confirms the necessity of linker optimization between modules in zinc finger domains.

## Introduction

The C2H2 type zinc fingers (ZFs) are modular domains that specifically recognize and bind bases in the double-stranded DNA (dsDNA), but they can also interact with RNA and proteins [1]. A single domain comprises a β-hairpin and an α-helix stabilized by the coordination of a zinc ion [2]. The helix contains four amino acid residues that form one-to-one contacts with four bases in the dsDNA substrate. A single module binds to three bases on one strand of the dsDNA and to a fourth base on the opposite strand. The recognition code for interaction with all possible DNA sequences had been determined, paving the way for rational design of zinc fingers with custom specificity [3].

The rules for ZF interaction with specific bases of the DNA sequence are well established. However, it was demonstrated that substitution of solely the amino acids directly involved in interaction with bases may not be sufficient to achieve a highly specific zinc finger. Other amino acid residues may also affect affinity for a given sequence, i.e. additional interactions, outside the canonical ones, with the substrate [4]. This affinity can also be influenced by the sequence and length of the linker between successive zinc fingers [5–8]. Engineering efforts were aimed in particular at expanding the recognition sequence by multimerization of zinc finger domains [9] and application of ZFs as targeting modules when fused with effector domains like nucleases, transcription activators and repressors [10]. Efforts were also made to obtain domains that bind other nucleic acids, like structured RNA [11] or specific sequences in DNA–RNA hybrids.

**Fig. 1:**
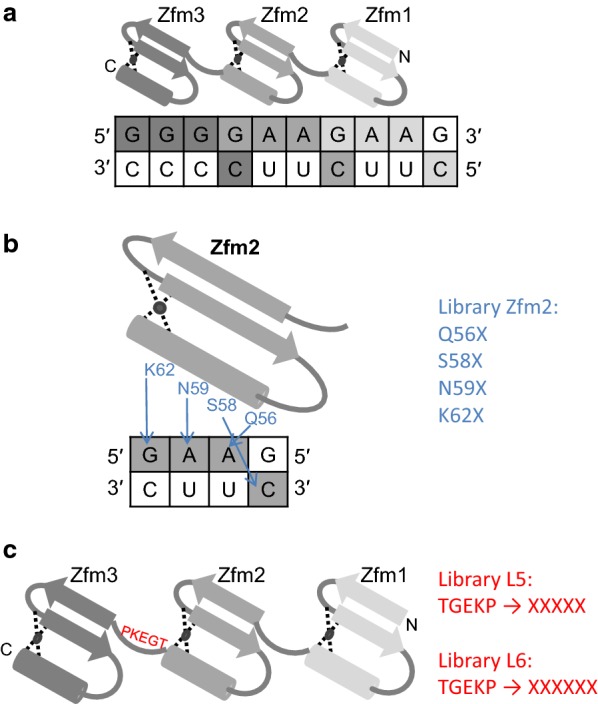
Schematic illustration of interaction between ZfQQR and the recognition sequence in DNA–RNA hybrid substrate. **a** Three zinc finger modules. **b** Zfm2 module and Zfm2 library design. The amino acid residues directly contacting bases of the substrate are labelled in blue. **c** Three zinc finger modules and the L5 and L6 libraries design. The sequence of the amino acid residues of the Zfm2–Zfm3 linker is shown in red

The artificial zinc finger ZfQQR was engineered to bind 5′GGGGAAGAA3′ sequence in the DNA strand of the DNA–RNA hybrid [12]. It comprises three zinc finger modules (Zfm), one that binds the first trinucleotide 5′GGG3′ and two binding the repeated 5′GAA3′ (Fig. 1a). It was used to construct a fusion enzyme with a ribonuclease H domain, turning a nonspecific processive enzyme into a sequence-specific one [13]. This opened the possibility of developing molecular tools for precise manipulation of RNA molecules, similar to how restriction enzymes are applied for dsDNA cleavage.

Here I present the efforts to determine the recognition code for the zinc fingers that bind specific sequences in DNA–RNA hybrids using directed evolution. It involved randomization of two regions in the ZfQQR. The first one encodes amino acid residues that directly recognize the bases in the DNA strand. The second targeted region was the linker between modules of the zinc finger domains. Randomization of the linker sequence involved additional elongation to potentially increase the protein’s affinity to a DNA–RNA hybrid. These processes were followed by selection using a panel of substrates for the first region, and the original binding sequence for the second.

## Main text

### Methods

See Additional file 1.

### Results and discussion

#### Directed evolution of ZfQQR variants

Directed evolution of the ZfQQR zinc finger was attempted by creating a library of variants using saturation mutagenesis by codon cassette insertion in target regions. T7 phage display was used in order to obtain phenotype-to-genotype linkage and selection of desired variants from the libraries was performed using biopanning. For this purpose, a modified version of the *zfqqr* gene with unique restriction sites around the target regions was cloned into a T7 phage downstream of the 10B capsid protein. In order to confirm that the T7ZfQQR recombinant phage expresses a functional zinc finger, biopanning was performed using a control mixture of T7 phages with recombinant T7ZfQQR in a 100:1 ratio. The control mixture was incubated with immobilized biotinylated substrate that contains the ZfQQR recognition sequence and an empty streptavidin-coated well (negative control). After the second round of biopanning, the ratio of T7:T7ZfQQR was 5:8 and 95:1 in substrate-immobilized and empty wells, respectively. The enrichment of the initial mixture with T7ZfQQR phages confirmed the functionality of the expressed zinc finger and effectiveness of the selection method.

Three libraries of genes encoding ZfQQR zinc finger variants, Zfm2, L5 and L6, were constructed. The Zfm2 library was designed to select domains with their sequence specificity altered by randomization of the residues directly interacting with the substrate (Q56, S58, N59 and K62) in the second zinc finger module (Fig. 1b). The L5 and L6 libraries were designed to enable selection of variants that are more selective towards DNA–RNA hybrids containing the target sequence. The region targeted for randomization was the Zfm2–Zfm3 linker. In the L5 library (Fig. 1c), the fragment encoding five amino acid residues (TGEKP) was randomized, whereas in the L6 library the randomized fragment was extended to six residues (Fig. 1c). The rationale behind the linker extension was that the structure of the DNA–RNA hybrid helix is an intermediate between two forms: A, with 11 base pairs per turn and B, with 10.5 base pairs per turn. The hybrid is slightly more packed in comparison to the B form of the dsDNA [14]. A longer, flexible linker might enable the modules to wrap around the DNA–RNA helix and fit better to the compressed structure than a shorter and more rigid one.

In all libraries, the selected codons were replaced by a degenerate NNS codon. After ligation of the library cassettes to the T7ZfQQR construct and in vitro packaging 3.3 × 10^5^ pfu/ml, 2.4 × 10^5^ pfu/ml, 2.1 × 10^6^ pfu/ml recombinant phages were obtained for the Zfm2, L5 and L6 libraries, respectively. Selection of variants from the Zfm2 library was carried out in parallel on a set of 64 biotinylated DNA–RNA hybrid substrates, each carrying a different possible variation of the three middle nucleotides in the recognition site (Fig. 1a, see Additional file 1: Table S1). This approach was aimed at determining the recognition code for binding DNA–RNA hybrids. Libraries L5 and L6 were selected using the original ZfQQR binding sequence. Phage libraries were biopanned for five rounds, the phage titer after each round varied from 10^5^ to 10^7^. The material after biopanning, input libraries and negative control (phage library Zfm2 biopanned on surface without substrate) were sequenced using MiSeq Illumina sequencer.

On average, 67 thousand reads were obtained with the correct length and sequence flanking the randomized regions for each sample. Distribution of the degenerate NNS sequence in the input Zfm2 library was uneven. The predominant codons encoded mainly P, F, L and V residues accounting for around 50% of reads, whereas the frequency should, in theory, be around 25% (see Additional file 2: Table S1). The most frequently appearing sequence encoded the motif PPPP and was present in 4.5% of all the filtered reads. For the input libraries L5 and L6, no bias in the amino acid distribution was observed (see Additional file 2: Tables S2 and S3).

**Fig. 2:**
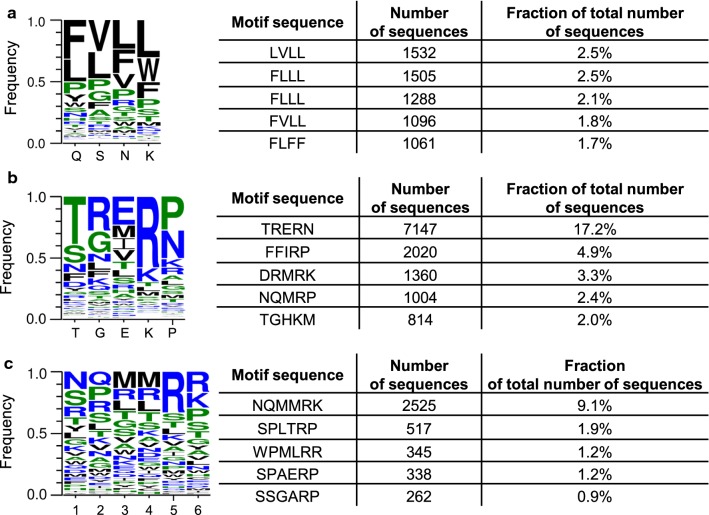
Sequence logos and five most abundant motifs obtained from sequenced fragments after 5 rounds of phage selection using a substrate containing the 5′GGGGAAGAA3′ sequence for: **a** library Zfm2, **b** library L5 and **c** library L6

In the case of variants derived after selection from the Zfm2 library and the negative control, a very similar distribution of amino acids was observed irrespective of the substrate sequence or the substrate’s presence during the selection (see Additional file 3: Table S1). All samples had a similar consensus sequence FVLL (example in Fig. 2a) where the consecutive letters of the motif correspond to the residues in the native protein Q56, S58, N59 and K62. Distribution of the amino acid residues in all sequenced samples resembled to a large extent the distribution of the input library Zfm2 (see Additional file 3: Table S1). The most prominent change observed in the isolated variants was the decline in the frequency of the PPPP motif. Most likely the selection pressure disfavored the presence of a conformationally rigid residue in the zinc finger alpha helix [15, 16]. The above results may have been caused by several factors: uneven distribution in the input Zfm2 library, insufficient selection pressure for randomization of the middle zinc finger module or the selection strategy that promoted binding of the DNA–RNA hybrid structure when central module’s substrate-contacting residues do not bind bases in nucleic acids [17].

Sequencing of the variants derived from the selection of the L5 library revealed that the predominant isolated amino acid sequence was TRERN (17% of obtained sequences, see Fig. 2b). For the L6 library the sequence NQMMRK (9% of obtained sequences, see Fig. 2c) was most frequently observed. None of the above two amino acid sequences appeared in the results from sequencing of the input libraries, which means that they were present less frequently than 1 in 55,162 for the L5 library and 1 in 42,323 for the L6 library. Interestingly, in case of the L5 library the sequence NQMRP, partially resembling the one isolated from the L6 library was the fourth most frequently appearing (Fig. 2b).

#### Binding affinity and selectivity of the isolated variants

The binding affinity of zinc finger variants selected using directed evolution was determined. For the Zfm2 library, the consensus sequence was chosen and variants of the zinc finger containing the Q56F S58V N59L K62L substitutions (termed ZfFVLL) only in the Zfm2 and in both, Zfm2 and Zfm3 (additional substitutions Q28F S30V N31L K34L, termed Zf2× FVLL) were obtained. The most frequently observed amino acid motifs obtained for the L5 and L6 libraries were introduced to the Zfm2–Zfm3 linker (termed ZfTRERN and ZfNQMMRK, respectively) and, additionally to the Zfm1–Zfm2 linker (termed Zf2× TRERN and Zf2× NQMMRK, respectively).

**Fig. 3:**
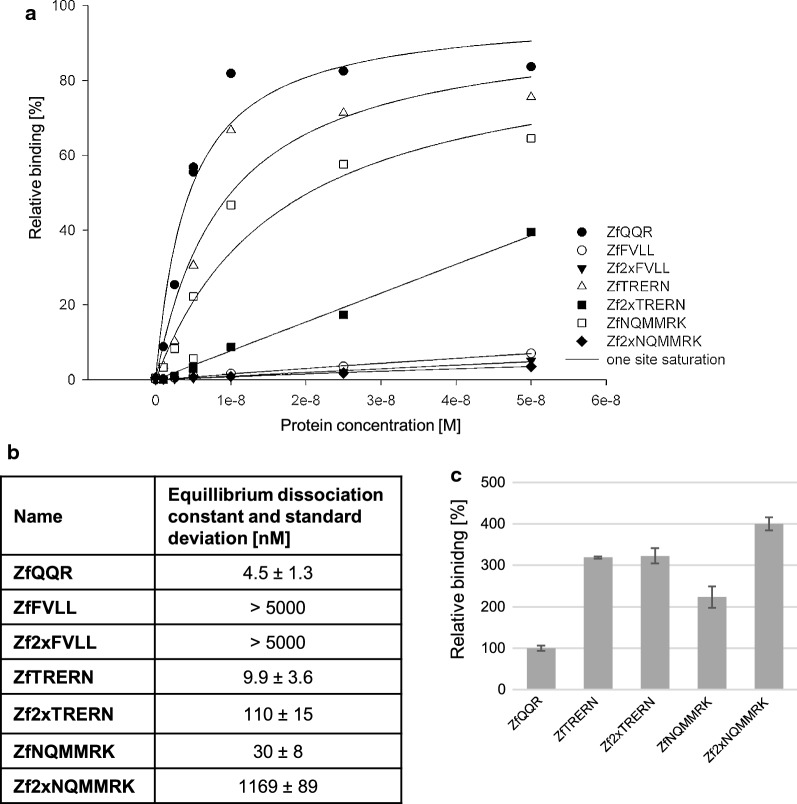
Relative binding of the sequence 5ʹGGGGAAGAA3ʹ by ZfQQR and variants. **a** Binding affinity measured using surface plasmon resonance on BIAcore 3000 instrument. **b** The equilibrium dissociation constants (K_D_) were obtained from global fitting of the results of affinity measurement using the ones site saturation model. **c** Relative binding in the presence of dsDNA competitor in 100-fold molar excess. 100% is the binding of ZfQQR to substrate with 5ʹGGGGAAGAA3ʹ sequence

For native ZfQQR and each protein variant, the equilibrium dissociation constant was measured using surface plasmon resonance (Fig. 3a). The K_D_ for the ZfFVLL and Zf2×FVLL zinc fingers was above 5000 nM and could not be measured using this method because the proteins were aggregating in the assay buffer at concentrations above 2 µM. This result of binding analysis and the sequencing results obtained from selection using a panel of 64 substrates as well as the negative control, support the explanation that the input library bias along with insufficient selection pressure hampered the biopanning. It is most likely that the selected variants result from the background nonspecific binding of phage particles to the streptavidin-coated wells.

The K_D_ of the ZfTRERN and ZfNQMMRK variants was slightly higher than the ZfQQR (Fig. 3b). However, when the motifs were repeated in the Zfm1–Zfm2 linker, the variants had tenfold and 40-fold higher K_D_ than the single motif variants. This result indicates that the engineering of linkers is localization specific and their optimization should be performed separately for each one.

In order to determine if the zinc finger variants were improved in their ability to discriminate between the DNA–RNA hybrids over dsDNA, their relative binding to the substrate with the 5′GGGGAAGAA3′ sequence in the presence of 100-fold excess of a dsDNA competitor (containing the 5′GGGGAAGAA3′ sequence) was measured using nitrocellulose filter binding assay. All single motif and double motif variants displayed at least twofold higher relative binding of the DNA–RNA hybrid than the original ZfQQR (Fig. 3c). Although the variants display a higher K_D_ than the ZfQQR, their selectivity for DNA–RNA hybrids over dsDNA improved. It might indicate that further optimization of preference for DNA–RNA hybrid vs. dsDNA binding is achievable and that it is distinct from optimization of the sequence selectivity.

## Limitations

Sequence bias in the Zfm2 input library resulted in overrepresentation of the P, F, L and V codons. The number of phage particles obtained after in vitro packaging was insufficient to represent all the possible codon combinations in the theoretical library. Affinity binding measurements using surface plasmon resonance were done as single experiments.

## Supplementary information

**Additional file 1.** The description of methods, scheme of a fragment of the capsid-zfqqr fusion (Figure S1), used oligonucleotides (Table S1) and buffer composition (Table S2).

**Additional file 2.** The theoretical and observed frequencies of amino acid residues in input library Zfm2 (Table S1), L5 (Table S2) and L6 (Table S3).

**Additional file 3.** The number of reads passing filters and frequency sequence logos of randomized regions in the selected variants from Zfm2 library after biopanning on a panel of substrates (Table S1), and from the L5 and L6 library (Table S2).

## Data Availability

The datasets used and/or analyzed during the current study are available from the corresponding author on reasonable request

## Abbreviations

dsDNA: double-stranded DNA
Zfm: zinc finger module

## References

[1] Iuchi S (2001). Three classes of C2H2 zinc finger proteins. Cell Mol Life Sci.

[2] Pavletich NP, Pabo CO (1993) Zinc finger-DNA recognition: crystal structure of a Zif268-DNA complex at 2.1 Angstroms. 10.2210/pdb1zaa/pdb. 10.1126/science.2028256 2028256

[3] Sera T, Uranga C (2002). Rational design of artificial zinc-finger proteins using a nondegenerate recognition code table. Biochemistry.

[4] Vandevenne M, Jacques DA, Artuz C, Nguyen CD, Kwan AHY, Segal DJ, Matthews JM, Crossley M, Guss JM, Mackay JP (2013). New insights into DNA recognition by zinc fingers revealed by structural analysis of the oncoprotein ZNF217. J Biol Chem.

[5] Ryan RF, Darby MK (1998). The role of zinc finger linkers in p43 and TFIIIA binding to 5S rRNA and DNA. Nucleic Acids Res.

[6] Moore M, Choo Y, Klug A (2001). Design of polyzinc finger peptides with structured linkers. Proc Natl Acad Sci U S A.

[7] Imanishi M, Sugiura Y (2002). Artificial DNA-bending six-zinc finger peptides with different charged linkers: distinct kinetic properties of DNA bindings†. Biochemistry.

[8] van Leeuwen HC, Strating MJ, Rensen M, de Laat W, van der Vliet PC (1997). Linker length and composition influence the flexibility of Oct-1 DNA binding. EMBO J.

[9] Beerli RR, Barbas CF (2002). Engineering polydactyl zinc-finger transcription factors. Nat Biotechnol.

[10] Klug A (2010). The discovery of zinc fingers and their applications in gene regulation and genome manipulation. Annu Rev Biochem.

[11] Chen Y, Varani G (2013). Engineering RNA-binding proteins for biology. FEBS J.

[12] Shi Y, Berg JM (1995). Specific DNA–RNA hybrid binding by zinc finger proteins. Science.

[13] Sulej AA, Tuszynska I, Skowronek KJ, Nowotny M, Bujnicki JM (2012). Sequence-specific cleavage of the RNA strand in DNA–RNA hybrids by the fusion of ribonuclease H with a zinc finger. Nucleic Acids Res.

[14] Fedoroff OY, Salazar M, Reid BR (1993). Structure of a DNA: RNA hybrid duplex. J Mol Biol.

[15] MacArthur MW, Thornton JM (1991). Influence of proline residues on protein conformation. J Mol Biol.

[16] Kim MK, Kang YK (1999). Positional preference of proline in alpha-helices. Protein Sci.

[17] Luscombe NM, Laskowski RA, Thornton JM (2001). Amino acid-base interactions: a three-dimensional analysis of protein-DNA interactions at an atomic level. Nucleic Acids Res.

