# Supplementary material for: Improving selectivity of DNA–RNA binding zinc finger using directed evolution
Source: BMC Res Notes. 2019 Dec 4;12:792. doi: 10.1186/s13104-019-4833-8 (PMC6894256; doi:10.1186/s13104-019-4833-8)
Supplement: Supplementary file 1 — Additional file 1. The description of methods, scheme of a fragment of the capsid-zfqqr fusion (Figure S1), used oligonucleotides (Table S1) and buffer composition (Table S2). [file 13104_2019_4833_MOESM1_ESM.docx]

**Additional file 1**

**Methods**

**Construction of phage libraries and biopanning**

The pET28zfqqr construct was obtained previously [[1]](https://paperpile.com/c/sST4ml/BpymZ). Three unique restriction sites (HindIII, Pfl23II and KpnI) were introduced as silent mutations flanking the target regions in the *zfqqr* gene sequence (Figure S1 below). The modified *zfqqr* gene was cloned into the BamHI and XhoI sites in the T7Select 10-3b vector (Novagen) yielding the T7ZfQQR construct. The libraries Zfm2, L5 and L6 were generated by restriction site-based cloning of synthetic oligonucleotide libraries with NNS degenerate codons in target positions (for sequence of oligonucleotides see Table S1 below). *In vitro* packaging, amplification of phages and plaque assays was done according to T7Select System Manual.

The biopanning of the recombinant phage libraries was performed using a panel of biotinylated DNA-RNA hybrids in which the RNA strand was labeled at the 5 'end with biotin enabling immobilization on streptavidin-coated surface (sequence of DNA oligonucleotides in Table S1 below). 5 pmols of hybrid was immobilized in streptavidin coated 96-well plates in TBST for 2 h in room temperature and washed three times with TBST. Phage binding to substrate was performed overnight in 4 ℃ in phage binding buffer (buffer composition in Table S2 below) with 100 µl 10^10^ pfu/ml for each library. After five washing steps with washing buffer, the bound phages were eluted for 20 min in room temperature using elution buffer. The phage titer was calculated and phages were amplified before the next round. Biopanning was done for five rounds. After the last selection cycle 132 bp (libraries Zfm2 and L5) or 135 bp (library L6) fragments of the *zfqqr* gene encoding enriched regions were PCR amplified, double indexes were added and the libraries were sequenced using Illumina MiSeq. Filtering involved removal of native *zfqqr* sequences that didn’t arise from the libraries and extraction of 63 bp or 66 bp nucleotide sequences encompassing target regions only from complete reads. Codons were translated to amino acid sequence and sequence logo was generated for each library using Web Logo 3 (in order to show frequencies, the composition adjustment was turned off) [[2, 3]](https://paperpile.com/c/sST4ml/YWEf+vdZ5).

**Zinc finger binding analysis**

For binding analyses the selected variants were cloned in pET28a vector without Hisx6 tag, expressed in *Escherichia coli* BL21 (DE3) strain, purified using preclearing of bacterial lysates by heat treatment, centrifugation and affinity chromatography of lysates on a heparin resin. Protein concentrations were calculated based on absorption at 280 nm measured with a NanoDrop 1000 spectrophotometer. Homogeneity of the protein samples was estimated to be ≥ 95% by SDS-PAGE.

Binding affinity was measured using BIAcore 3000 instrument. Approximately 70 response units of biotinylated ZfQQR DNA-RNA hybrid substrate (sequence in Table S1 below) was immobilized on a flow cell of streptavidin-coated sensor chip (XanTec). The cell without immobilized substrate was used as a reference. The zinc finger proteins at concentrations ranging from 20-2000 nM were injected in Zf binding buffer at flow rate 50 µl/min for 3 min, followed by a 15 min dissociation phase and sensor regeneration with 2 M NaCl. The value of the equilibrium dissociation constant (K_D_) was obtained by fitting a plot of response at equilibrium against the zinc finger protein concentration (SigmaPlot 10, Ligand Binding Module, one site saturation equation).

The selectivity towards DNA-RNA hybrids in the presence of an excess of a double-stranded DNA competitor was determined in nitrocellulose filter binding. An oligonucleotide with the ZfQQR binding site (ZfQQR hybrid, see Table S1 below) was radiolabeled with [γ-33P]ATP and T4 polynucleotide kinase, annealed to the complementary RNA oligonucleotide and purified on Sephadex G-25 to remove the unincorporated label. The binding reactions were performed in Zf binding buffer for 15 min at room temperature in a total volume of 50 μl with 0.1 μM 20 bp ZfQQR DNA-RNA hybrid (1:100 ratio of  ^33^P end-labeled to unlabelled substrate), 10 μM unlabelled dsDNA competitor and 2 μM zinc finger protein. Reactions were incubated 30 min at 25°C and filtered through 0.22 mm nitrocellulose filter (Whatman) in Dot-Blot apparatus (Bio-Rad). Each well was washed three times with 400 µl of the binding buffer. Dried filters were exposed to a phosphoimager screen overnight. Images were scanned on a Storm Phosphorimager, and the retained radioactivity was quantified using ImageQuant software (Amersham). For each protein triplicate measurements of binding were made and standard deviation was calculated.


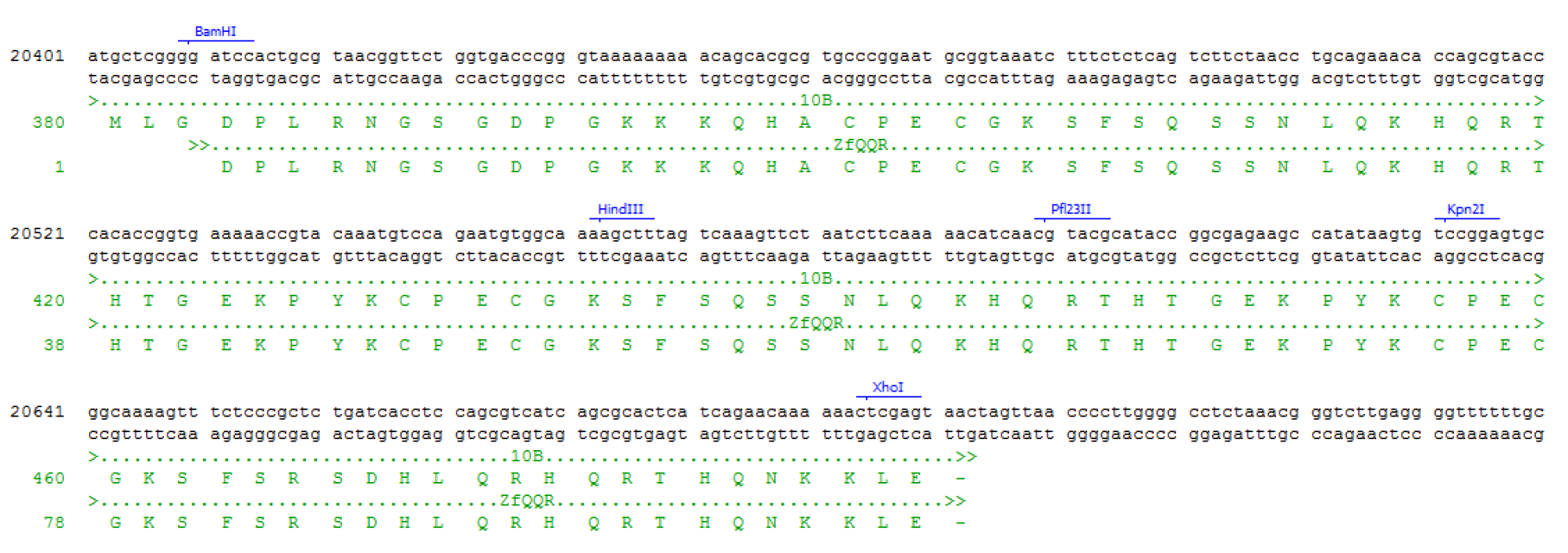


**Figure S1.** The nucleotide (small letters) and amino acid (capital letters) fragment sequence of the fusion of 10A capsid and *zfqqr* gene with relevant restriction sites used for cloning.

**Table S1**. Sequences of oligonucleotides and DNA fragments used in experiments. The ZfQQR recognition sequence is underlined. For oligonucleotides used for biopanning only the DNA strand is given and the three nucleotide sequence variation from the original ZfQQR recognition sequence is in bold NNN.

| **Name** | **Sequence** | | | | | | | | | |
| --- | --- | --- | --- | --- | --- | --- | --- | --- | --- | --- |
| Zfm2 cassette | CAAAAGCTTTAGTNNSAGTNNSNNSCTTCAANNSCATCAACGTACGCATA | | | | | | | | | |
| L5 cassette | CAACGTACGCATNNSNNSNNSNNSNNSTATAAGTGTCCGGAGCCG | | | | | | | | | |
| L6 cassette | CAACGTACGCATNNSNNSNNSNNSNNSNNSTATAAGTGTCCGGAGCCG | | | | | | | | | |
| Biacore hybrid | RNA strand | | Biotin-GAUCGAUUCUUCCCCUGAUCGA | | | | | | | |
|  | DNA strand | | TCGATCAGGGGAAGAATCGATC | | | | | | | |
| Zf-QQR hybrid | RNA strand | | GAAAGGAGGAACUAUAUCGAGGAUUCUUCUUCCCCAGUGA | | | | | | | |
|  | DNA strand | | TCACTGGGGAAGAAGAATCCTCGATATAGTTCCTCCTTTC | | | | | | | |
| dsDNA competitor | forward strand | | TCACTGGGGAAGAAGAATCCTCGATATAGTTCCTCCTTTC | | | | | | | |
|  | reverse strand | | GAAAGGAGGAACTATATCGAGGATTCTTCTTCCCCAGTGA | | | | | | | |
| DNA oligonucleotide for biopanning | TCGATCAGGG**NNN**GAATCGATC | | | | | | | | | |
|  | **NNN**= | GGG | | GTA | AAG | ATA | TTG | TAA | CCG | CAA |
|  |  | GGA | | GCA | AAA | ACA | TTA | TCA | CCA | CTA |
|  |  | GGT | | GAT | AAT | AGT | TTT | TGT | CCT | CGT |
|  |  | GGC | | GTT | AAC | ATT | TTC | TAT | CCC | CAT |
|  |  | GAG | | GCT | AGG | ACT | TGG | TCT | CGG | CTT |
|  |  | GTG | | GAC | ATG | AGC | TAG | TGC | CAG | CGC |
|  |  | GCG | | GTC | ACG | ATC | TCG | TAC | CTG | CAC |
|  |  | GAA | | GCC | AGA | ACC | TGA | TCC | CGA | CTC |

**Table S2**. Composition of buffers used in experiments.

| **Buffer name** | **Composition** |
| --- | --- |
| TBST | 50 mM Tris-Cl pH 7.6, 150 mM NaCl, 0.05% Tween 20 |
| Phage binding buffer | 20 mM Tris-HCl pH 8.0, 90 mM KCl, 1 mM MgSO_4_, 20 µM ZnSO_4_, 1 mM DTT, 5% glycerol, 0.05% Tween 20, 0.2 µg poli dI-dC/ml |
| Phage wash buffer | 20 mM Tris-HCl pH 8.0, 90 mM KCl, 1 mM MgSO_4_, 20 µM ZnSO_4_, 1 mM DTT, 5% glycerol, 0.05% Tween 20 |
| Elution buffer | 20 mM Tris-HCl pH 8.0, 4M NaCl, 1 mM MgSO_4_, 20 µM ZnSO_4_ |
| Zf binding buffer | 20 mM Tris-HCl pH 8.0, 100 mM KCl, 20 µM ZnSO_4_, 2 mM DTT, 10 µg/ml BSA |

**Bibliography**

1. [1] [Sulej AA, Tuszynska I, Skowronek KJ, Nowotny M, Bujnicki JM (2012) Sequence-specific cleavage of the RNA strand in DNA-RNA hybrids by the fusion of ribonuclease H with a zinc finger. Nucleic Acids Res 40:11563–11570](http://paperpile.com/b/sST4ml/BpymZ)

2. [2] [Crooks GE, Hon G, Chandonia J-M, Brenner SE (2004) WebLogo: a sequence logo generator. Genome Res 14:1188–1190](http://paperpile.com/b/sST4ml/YWEf)

3. [3] [Schneider TD, Stephens RM (1990) Sequence logos: a new way to display consensus sequences. Nucleic Acids Res 18:6097–6100](http://paperpile.com/b/sST4ml/vdZ5)
