# Supplementary material for: Improving selectivity of DNA–RNA binding zinc finger using directed evolution
Source: BMC Res Notes. 2019 Dec 4;12:792. doi: 10.1186/s13104-019-4833-8 (PMC6894256; doi:10.1186/s13104-019-4833-8)
Supplement: Supplementary file 2 — Additional file 2. The theoretical and observed frequencies of amino acid residues in input library Zfm2 (Table S1), L5 (Table S2) and L6 (Table S3). [file 13104_2019_4833_MOESM2_ESM.docx]

**Additional file 2**

**Table S1.** Frequencies of amino acid residues in input library Zfm2 (total 36722 reads passing filters). Most frequently occurring amino acid residues are marked in bold.

| **Amino acid** | **Theoretical frequency** | **Randomized position** | | | |
| --- | --- | --- | --- | --- | --- |
|  |  | **Q54** | **S58** | **N57** | **K62** |
| **A** | *6.3* | 2.9 | 8.8 | 4.7 | 0.4 |
| **C** | *3.1* | 2.8 | 1.3 | 2.5 | 2.7 |
| **D** | *3.1* | 2.2 | 1.6 | 0.9 | 0.1 |
| **E** | *3.1* | 0.7 | 2.7 | 0.8 | 0.1 |
| **F** | *3.1* | **13.0** | 4.9 | **9.6** | 7.3 |
| **G** | *6.3* | 0.6 | 7.1 | 3.3 | 0.2 |
| **H** | *3.1* | 5.8 | 2.3 | 3.1 | 2.6 |
| **I** | *3.1* | 2.8 | 1.0 | 1.9 | 1.5 |
| **K** | *3.1* | 1.1 | 1.1 | 0.1 | 3.3 |
| **L** | *9.4* | 9.4 | 11.2 | **13.5** | **16.3** |
| **M** | *3.1* | 0.8 | 1.2 | 2.0 | 2.1 |
| **N** | *3.1* | 6.4 | 2.7 | 2.4 | 3.5 |
| **P** | *6.3* | **28.0** | **24.6** | **22.9** | **20.9** |
| **Q** | *3.1* | 0.6 | 0.6 | 0.7 | 1.5 |
| **R** | *9.4* | 2.0 | 3.8 | 5.6 | 6.1 |
| **S** | *9.4* | 8.2 | 6.2 | 7.7 | 11.1 |
| **T** | *6.3* | 5.2 | 4.2 | 7.3 | 9.6 |
| **V** | *6.3* | 2.3 | **12.0** | 6.2 | 0.3 |
| **W** | *3.1* | 1.0 | 1.0 | 2.5 | 7.5 |
| **Y** | *3.1* | 4.1 | 1.0 | 1.4 | 1.4 |
| **(STOP)** | *3.1* | 0.1 | 0.7 | 0.9 | 1.6 |

**Table S2.** Frequencies of amino acid residues in input library L5 (55365 reads passing filters).

| **Amino acid** | **Theoretical distribution** | **Randomized amino acid residue** | | | | |
| --- | --- | --- | --- | --- | --- | --- |
|  |  | **T** | **G** | **E** | **K** | **P** |
| **A** | *6.3* | 4.1 | 4.5 | 6.3 | 5.8 | 5.9 |
| **C** | *3.1* | 3.0 | 2.6 | 2.4 | 2.5 | 2.5 |
| **D** | *3.1* | 3.0 | 3.4 | 3.6 | 3.4 | 3.9 |
| **E** | *3.1* | 3.1 | 3.3 | 3.1 | 2.9 | 3.8 |
| **F** | *3.1* | 3.1 | 2.8 | 2.5 | 2.4 | 2.0 |
| **G** | *6.3* | 4.6 | 4.4 | 5.3 | 5.0 | 6.7 |
| **H** | *3.1* | 3.5 | 3.7 | 3.5 | 3.9 | 3.4 |
| **I** | *3.1* | 3.8 | 3.6 | 3.7 | 3.5 | 3.2 |
| **K** | *3.1* | 4.7 | 4.6 | 3.9 | 3.6 | 4.3 |
| **L** | *9.4* | 7.9 | 8.3 | 7.9 | 8.5 | 6.8 |
| **M** | *3.1* | 3.7 | 3.7 | 3.8 | 3.5 | 3.4 |
| **N** | *3.1* | 5.0 | 4.7 | 4.4 | 4.2 | 4.4 |
| **P** | *6.3* | 5.1 | 5.4 | 6.3 | 7.6 | 5.8 |
| **Q** | *3.1* | 2.9 | 3.3 | 3.0 | 3.4 | 3.0 |
| **R** | *9.4* | 8.4 | 8.3 | 8.4 | 8.3 | 9.7 |
| **S** | *9.4* | 9.7 | 9.4 | 8.8 | 8.8 | 8.8 |
| **T** | *6.3* | 7.7 | 8.1 | 8.4 | 8.5 | 7.8 |
| **V** | *6.3* | 5.3 | 4.7 | 6.0 | 5.5 | 6.1 |
| **W** | *3.1* | 2.9 | 3.0 | 2.4 | 2.6 | 2.5 |
| **Y** | *3.1* | 4.6 | 4.2 | 3.4 | 3.3 | 3.3 |
| **(STOP)** | *3.1* | 3.9 | 4.0 | 2.9 | 2.9 | 2.8 |

**Table S3**. Frequencies of amino acid residues in input library L6 (42472 reads passing filters).

| **Amino acid** | **Theoretical distribution** | **Randomized position in linker** | | | | | |
| --- | --- | --- | --- | --- | --- | --- | --- |
|  |  | **1st** | **2nd** | **3rd** | **4th** | **5th** | **6th** |
| **A** | *6.3* | 4.0 | 4.5 | 5.5 | 5.0 | 5.6 | 5.8 |
| **C** | *3.1* | 2.5 | 3.0 | 2.5 | 3.2 | 2.8 | 3.4 |
| **D** | *3.1* | 2.9 | 3.5 | 3.8 | 3.7 | 4.4 | 3.9 |
| **E** | *3.1* | 3.2 | 3.2 | 3.5 | 3.2 | 3.5 | 3.3 |
| **F** | *3.1* | 2.7 | 2.8 | 2.3 | 2.4 | 2.2 | 2.2 |
| **G** | *6.3* | 4.9 | 5.0 | 5.5 | 5.8 | 6.5 | 7.1 |
| **H** | *3.1* | 3.6 | 3.4 | 3.6 | 3.8 | 3.8 | 3.8 |
| **I** | *3.1* | 3.9 | 3.4 | 3.4 | 3.2 | 3.6 | 3.0 |
| **K** | *3.1* | 5.5 | 4.5 | 4.2 | 4.3 | 4.1 | 3.4 |
| **L** | *9.4* | 7.6 | 7.3 | 7.8 | 7.0 | 7.2 | 6.6 |
| **M** | *3.1* | 3.8 | 3.4 | 3.7 | 3.0 | 3.4 | 2.4 |
| **N** | *3.1* | 4.8 | 5.0 | 4.8 | 4.7 | 5.1 | 4.5 |
| **P** | *6.3* | 5.6 | 5.0 | 6.3 | 5.6 | 5.6 | 6.4 |
| **Q** | *3.1* | 3.3 | 3.3 | 3.3 | 3.3 | 3.2 | 3.0 |
| **R** | *9.4* | 9.4 | 9.0 | 9.1 | 9.6 | 9.7 | 10.8 |
| **S** | *9.4* | 9.1 | 9.7 | 8.7 | 9.7 | 8.3 | 9.3 |
| **T** | *6.3* | 8.2 | 7.2 | 7.6 | 7.4 | 7.1 | 7.2 |
| **V** | *6.3* | 5.1 | 4.6 | 5.4 | 4.5 | 5.7 | 4.9 |
| **W** | *3.1* | 2.8 | 3.6 | 2.4 | 3.1 | 2.3 | 2.7 |
| **Y** | *3.1* | 3.5 | 4.3 | 3.5 | 3.9 | 3.2 | 3.9 |
| **(STOP)** | *3.1* | 3.4 | 4.1 | 3.0 | 3.4 | 2.6 | 2.5 |
