# Supplementary material for: Improving selectivity of DNA–RNA binding zinc finger using directed evolution
Source: BMC Res Notes. 2019 Dec 4;12:792. doi: 10.1186/s13104-019-4833-8 (PMC6894256; doi:10.1186/s13104-019-4833-8)
Supplement: Supplementary file 3 — Additional file 3. The number of reads passing filters and frequency sequence logos of randomized regions in the selected variants from Zfm2 library after biopanning on a panel of substrates (Table S1), and from the L5 and L6 library (Table S2). [file 13104_2019_4833_MOESM3_ESM.docx]

**Additional file 3**

**Table S1**. Sequencing results of selected variants from the biopanning of the T7ZfQQR expressing the Zfm2 library on a panel of substrates. The frequency logo was generated using all extracted amino acid residue sequences, where first, second, third and fourth column is the frequency obtained for randomized positions Q56, S58, N59 and K62, respectively.

| **Sample number** | **Nucleotide sequence used for selection of second module** | **Number of reads passing filters** | **Frequency logo of amino acid residues from randomized region** |
| --- | --- | --- | --- |
| 1 | GGG | 44832 | 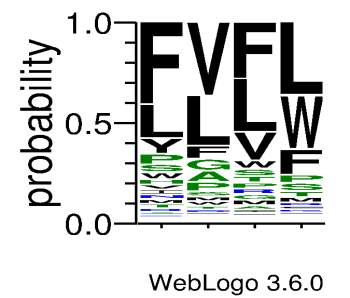 |
| 2 | GGA | 21417 | 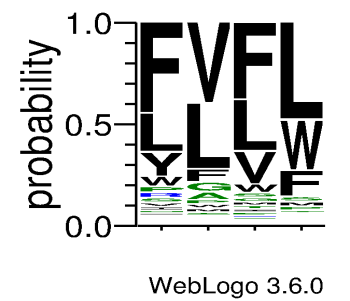 |
| 3 | GGT | 163949 | 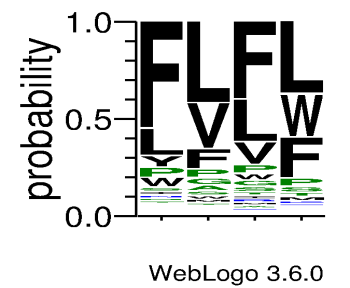 |
| 4 | GGC | 111596 | 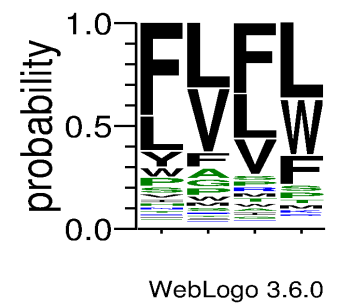 |
| 5 | GAG | 149445 | 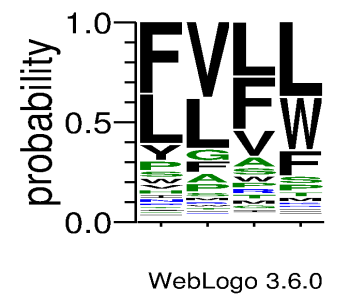 |
| 6 | GTG | 87366 | 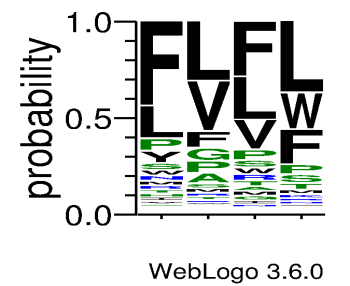 |
| 7 | GCG | 63335 | 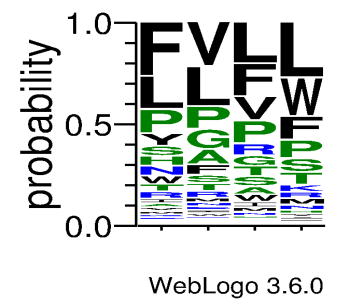 |
| 8 | GAA | 60898 | 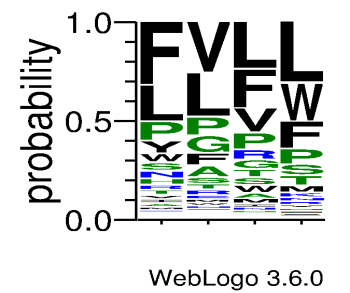 |
| 9 | GTA | 53852 | 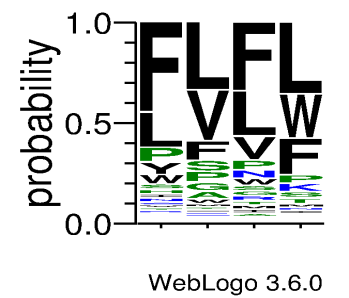 |
| 10 | GCA | 50223 | 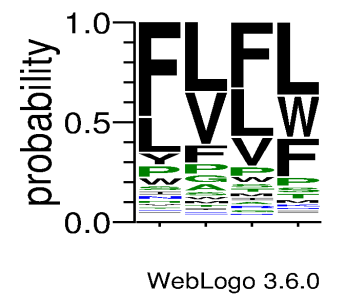 |
| 11 | GAT | 53432 | 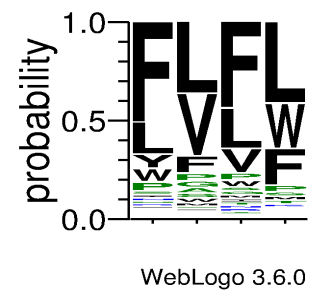 |
| 12 | GTT | 50531 | 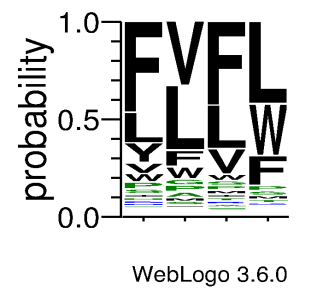 |
| 13 | GCT | 54399 | 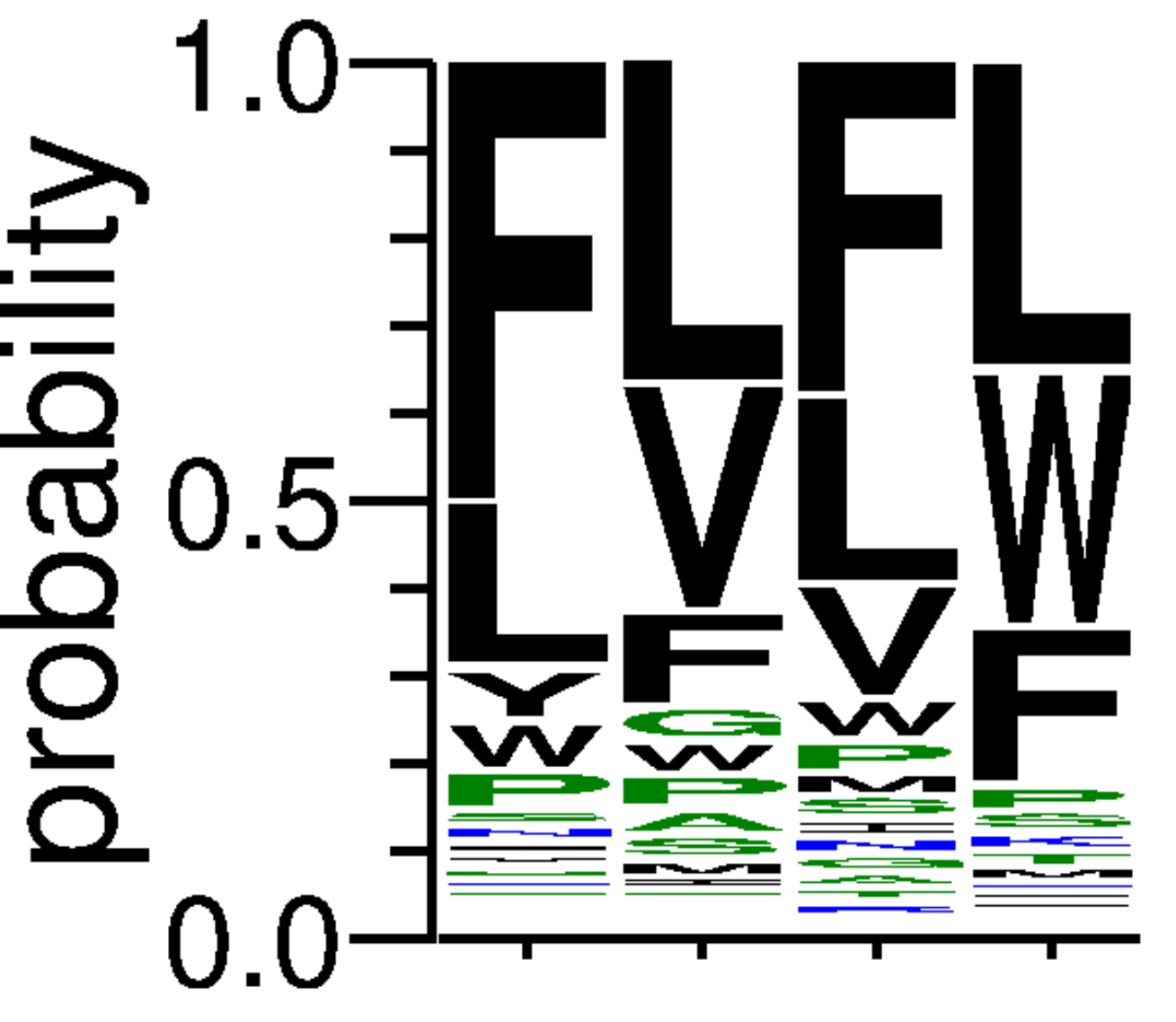 |
| 14 | GAC | 22991 | 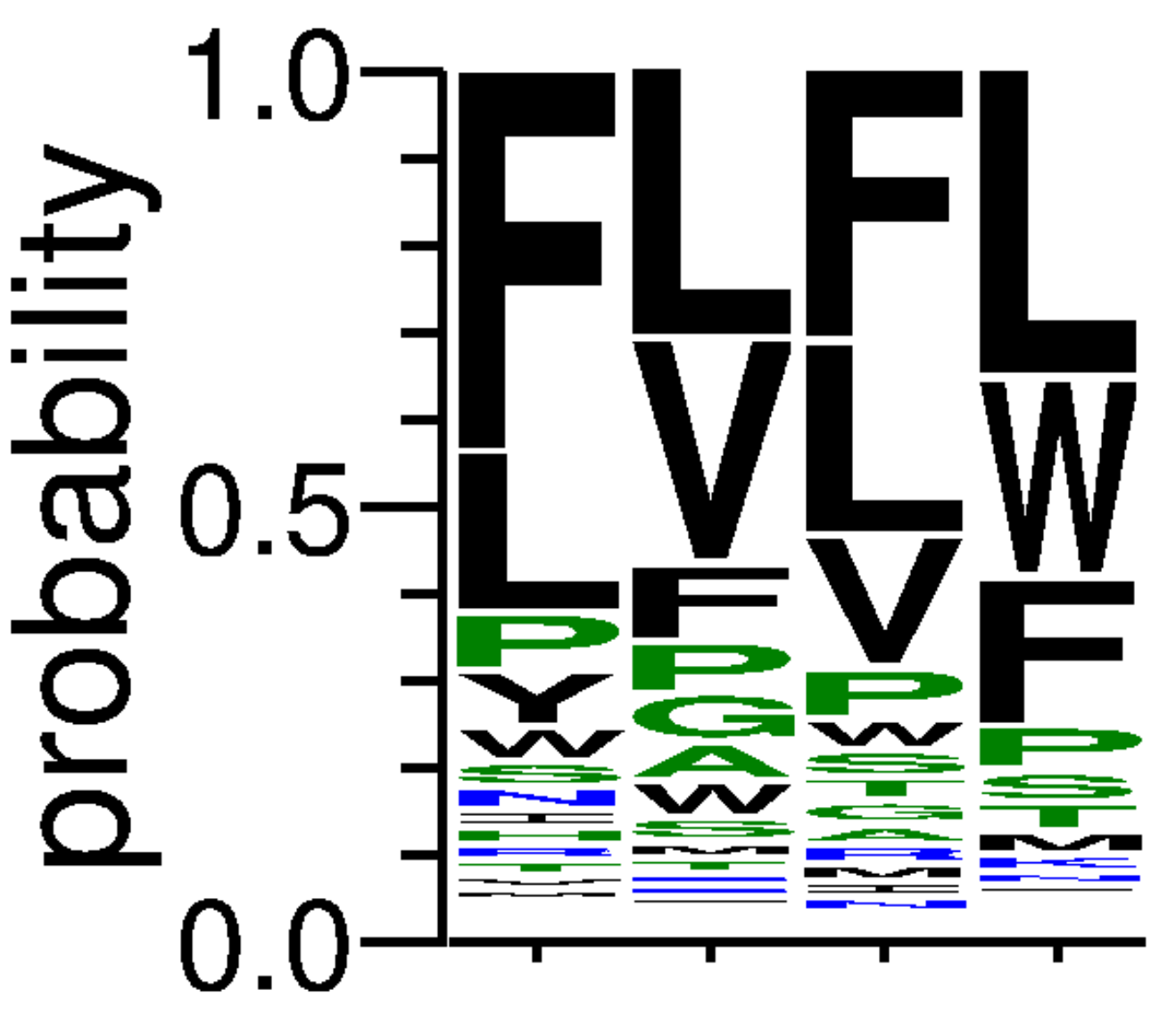 |
| 15 | GTC | 44998 | 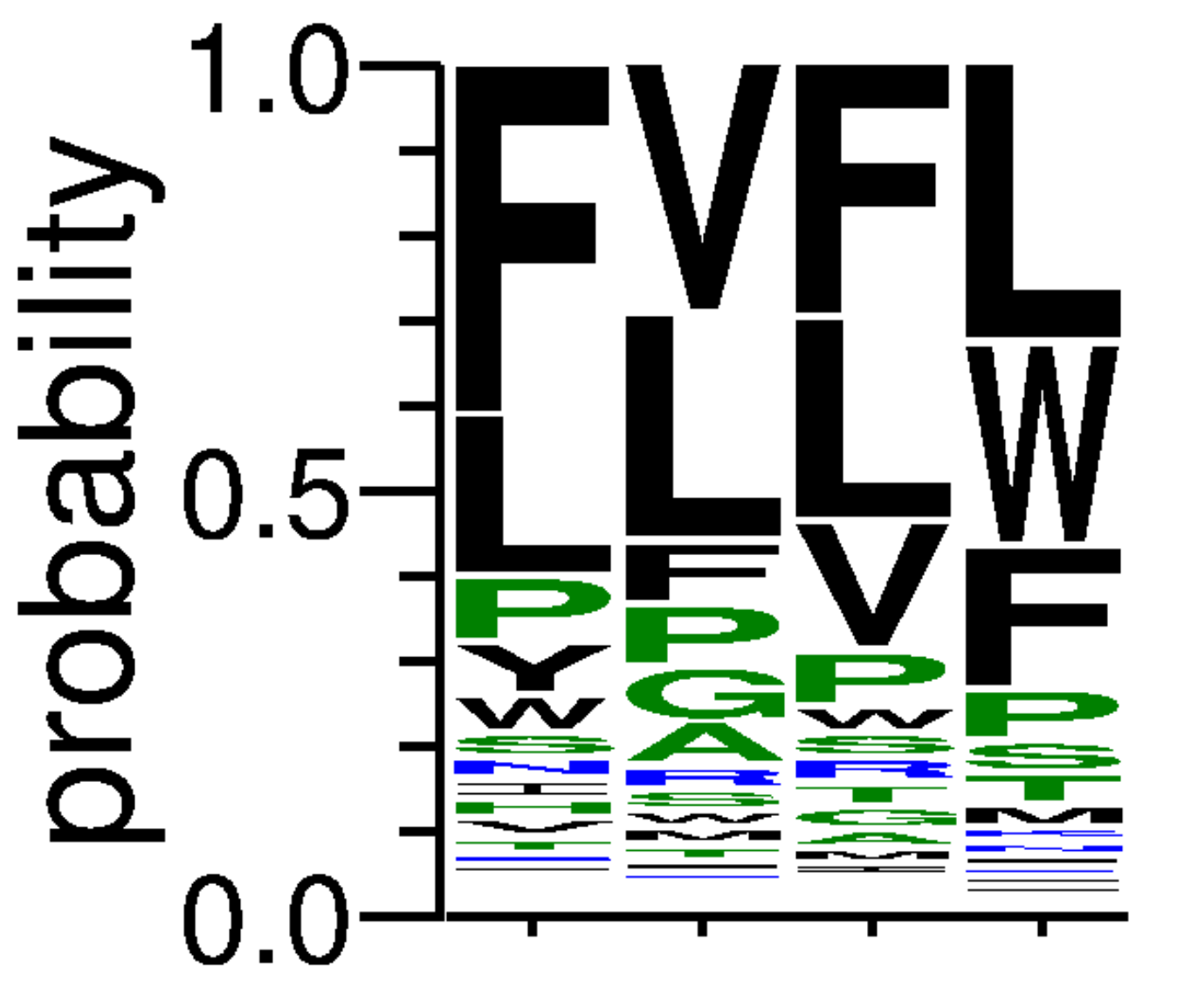 |
| 16 | GCC | 25933 | 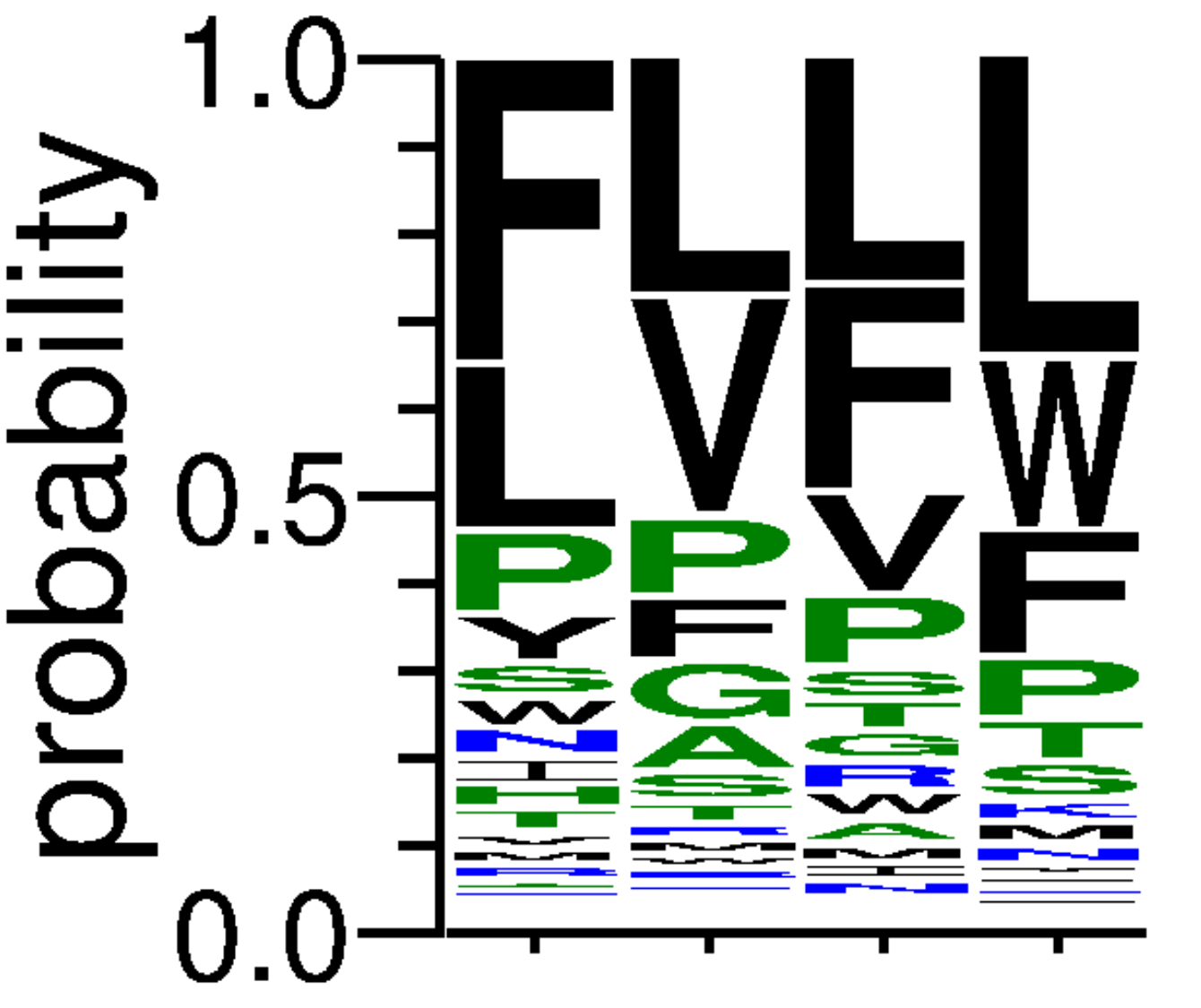 |
| 17 | AAG | 39927 | 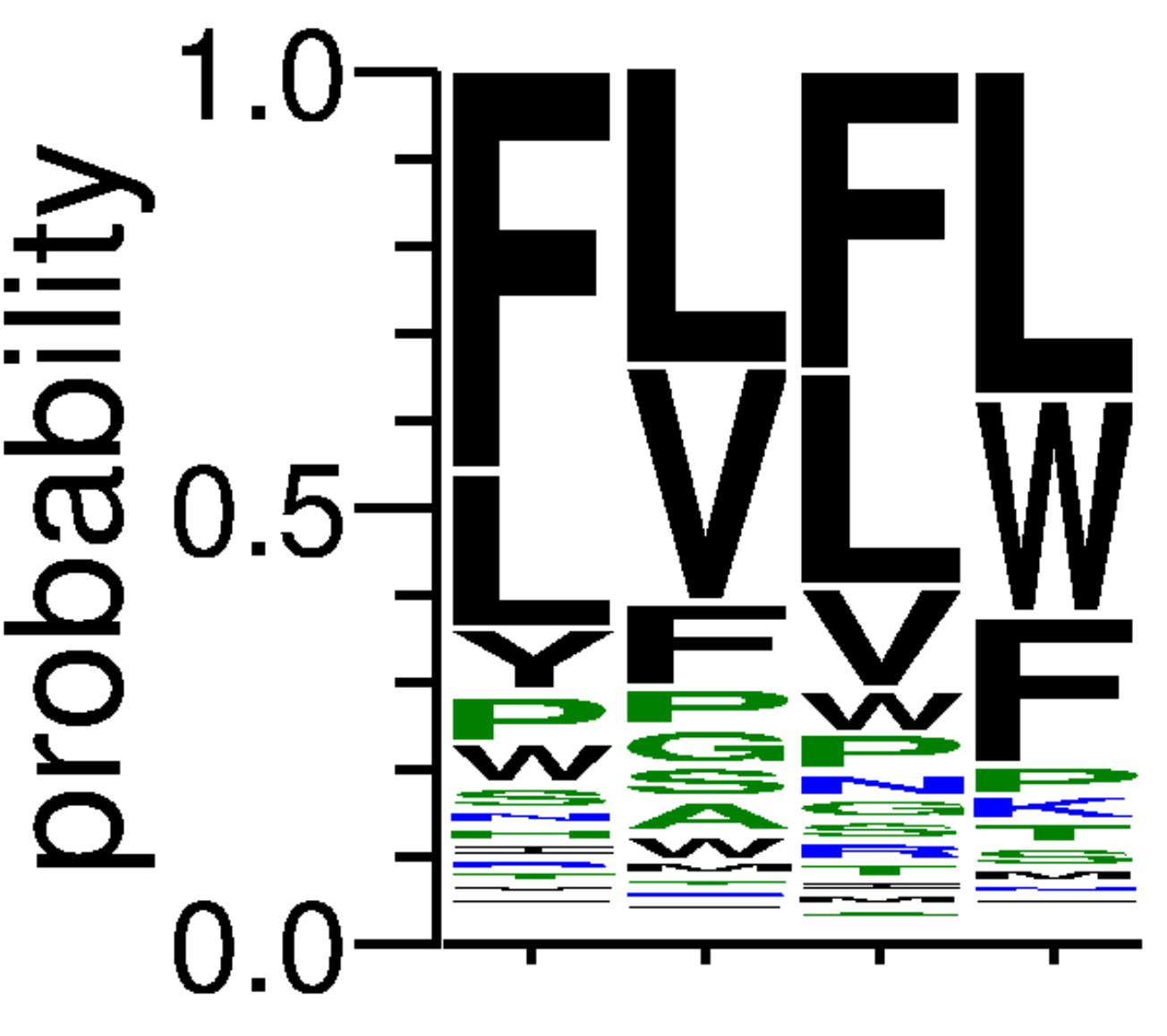 |
| 18 | AAA | 56184 | 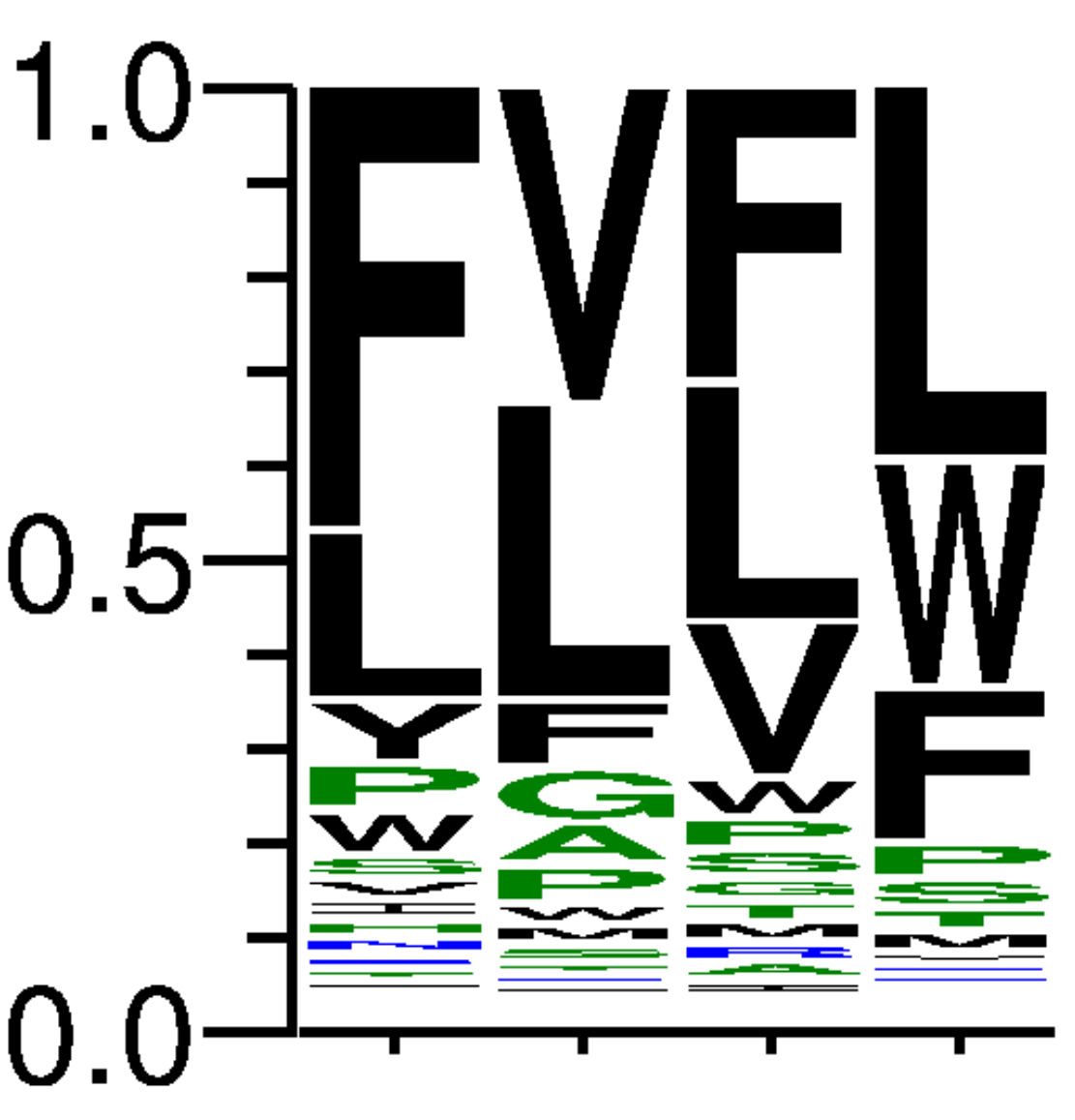 |
| 19 | AAT | 68649 | 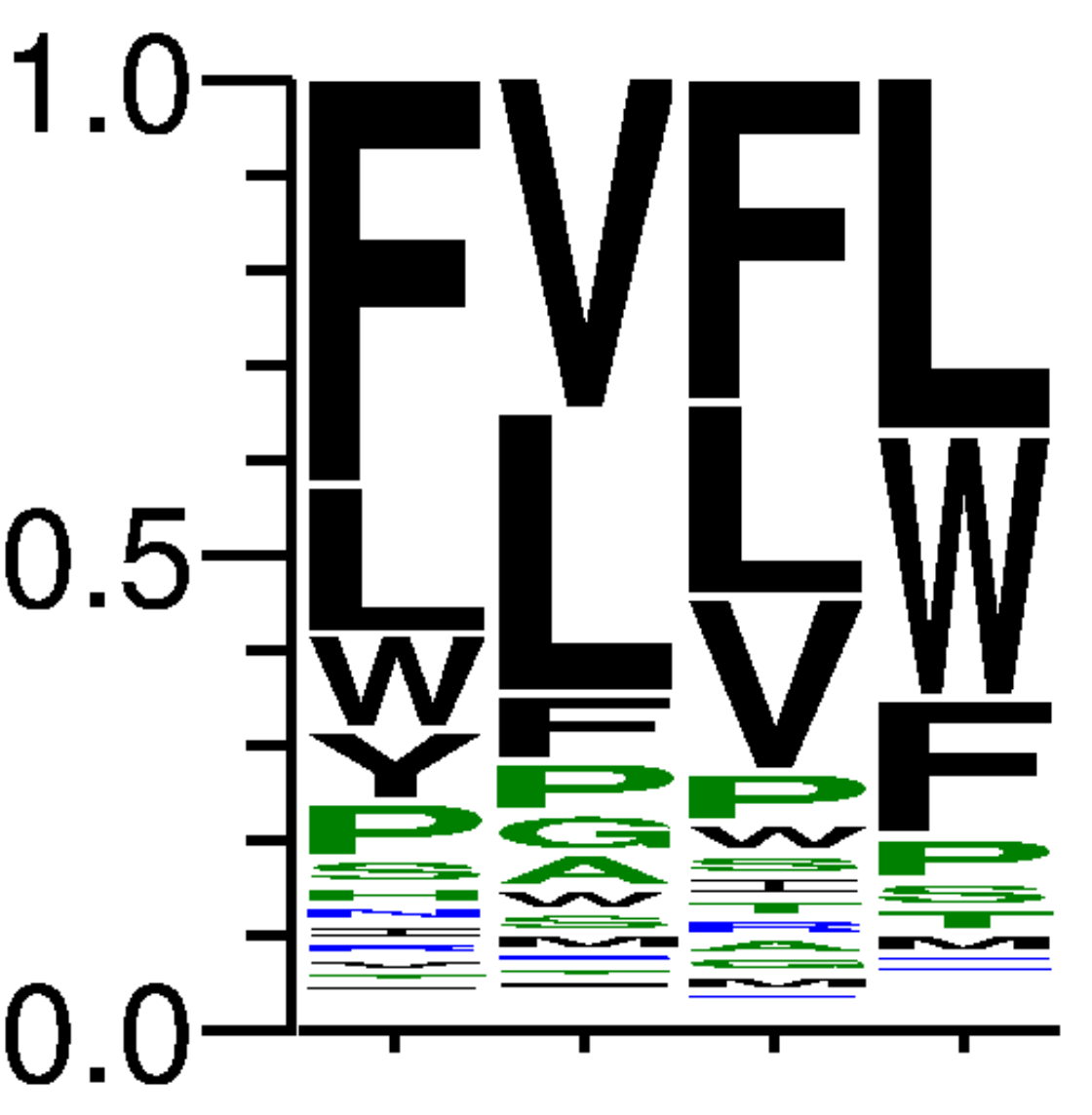 |
| 20 | AAC | 41015 | 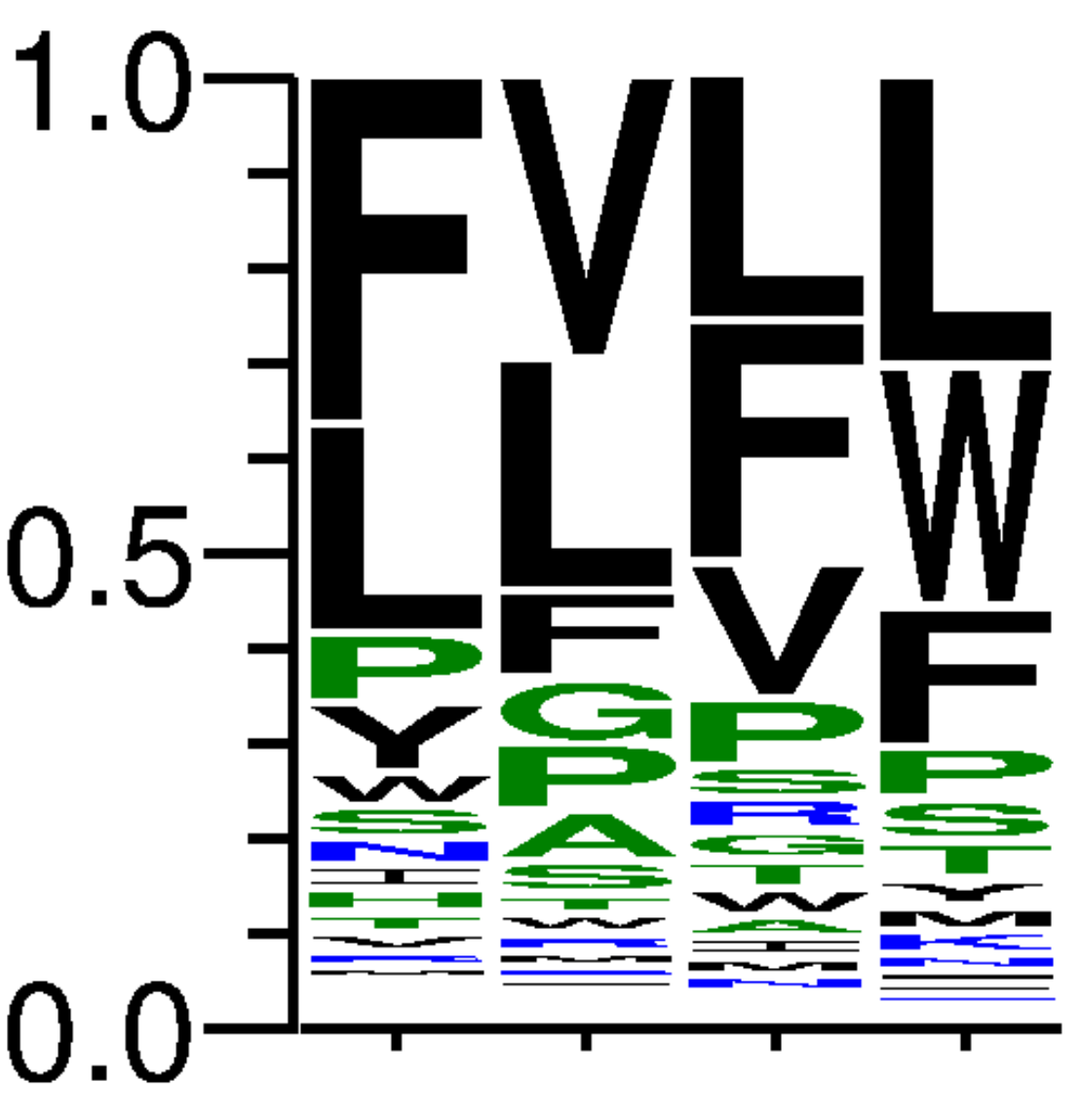 |
| 21 | AGG | 46739 | 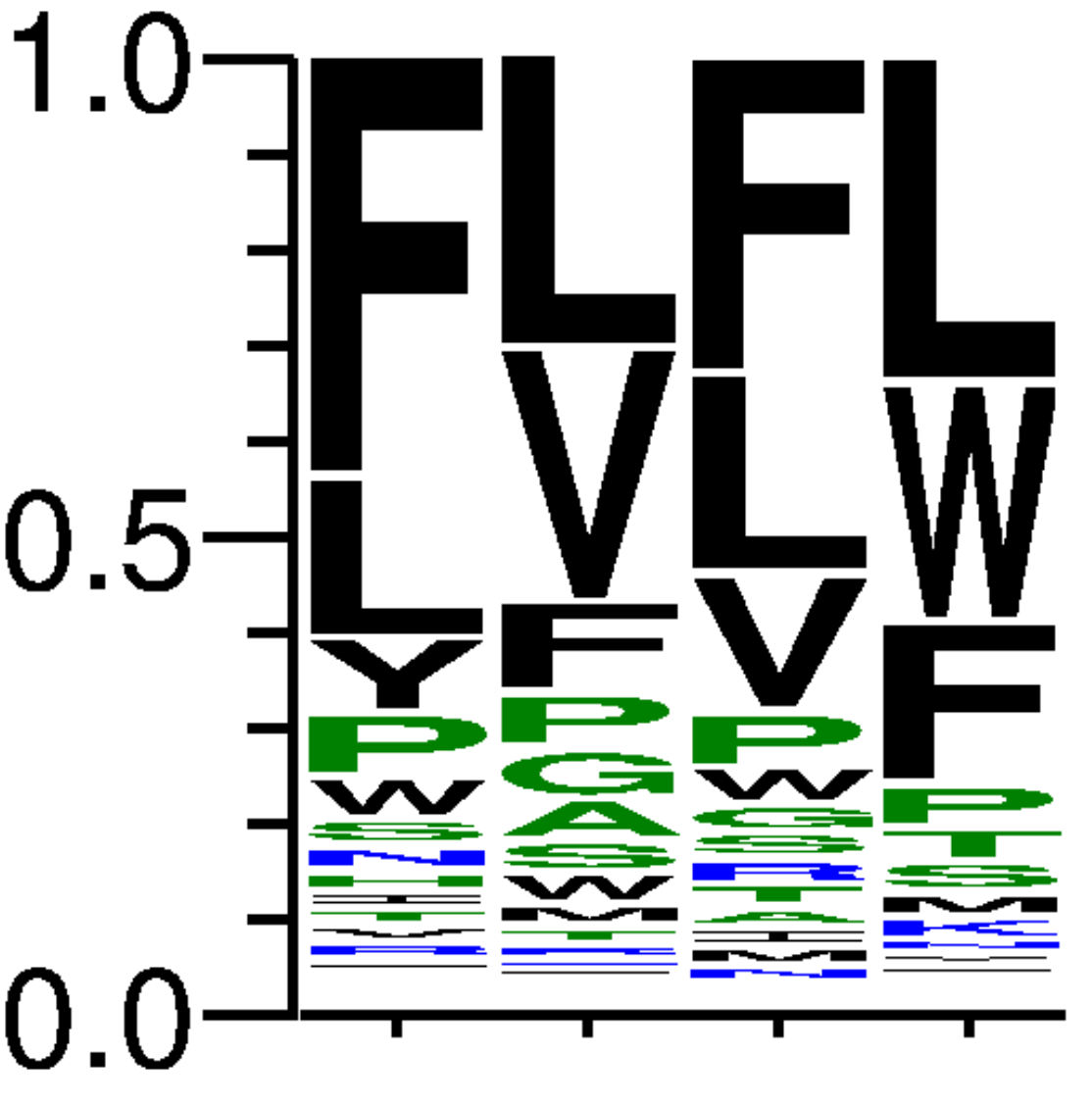 |
| 22 | ATG | 72661 | 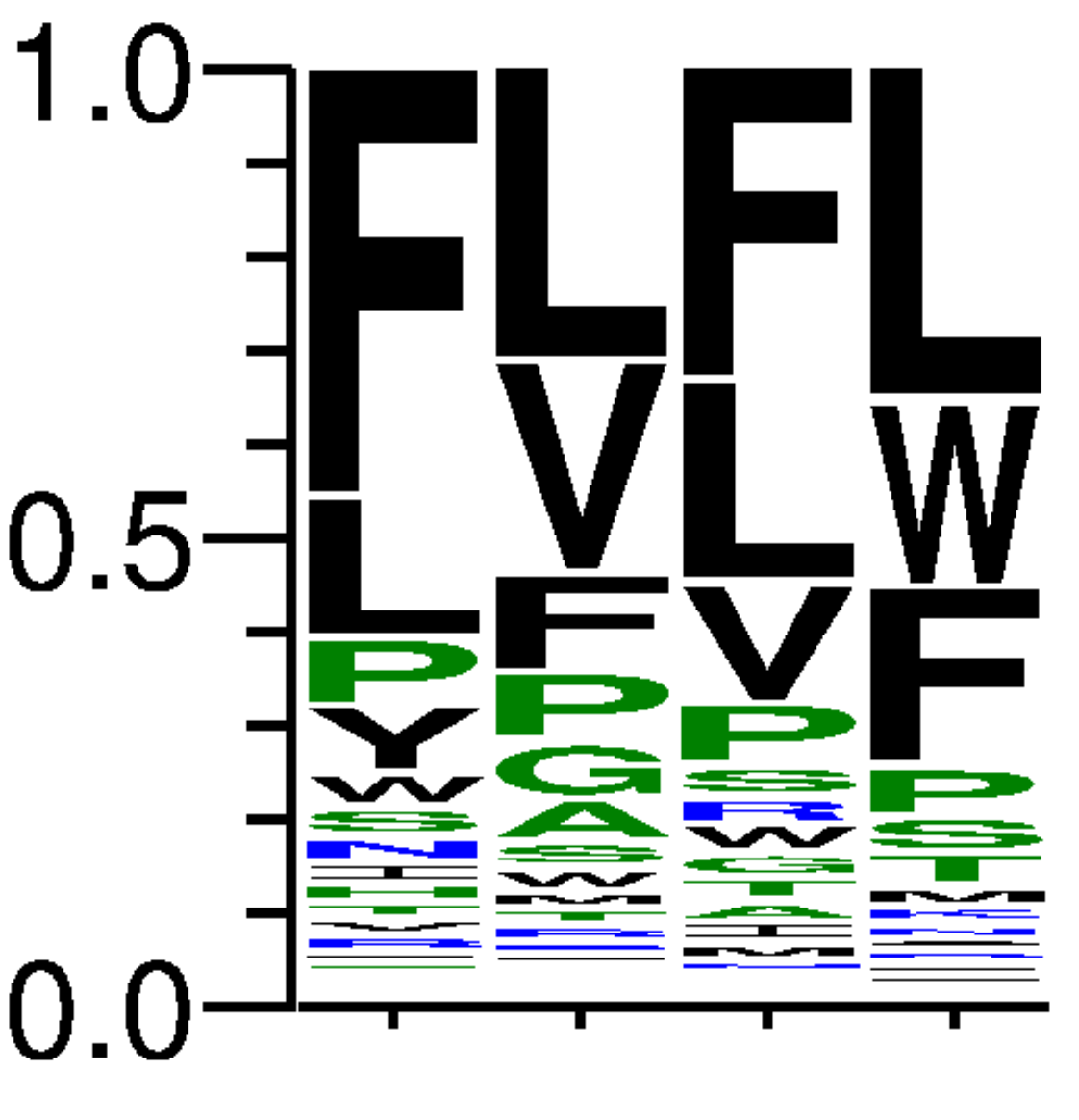 |
| 23 | ACG | 38390 | 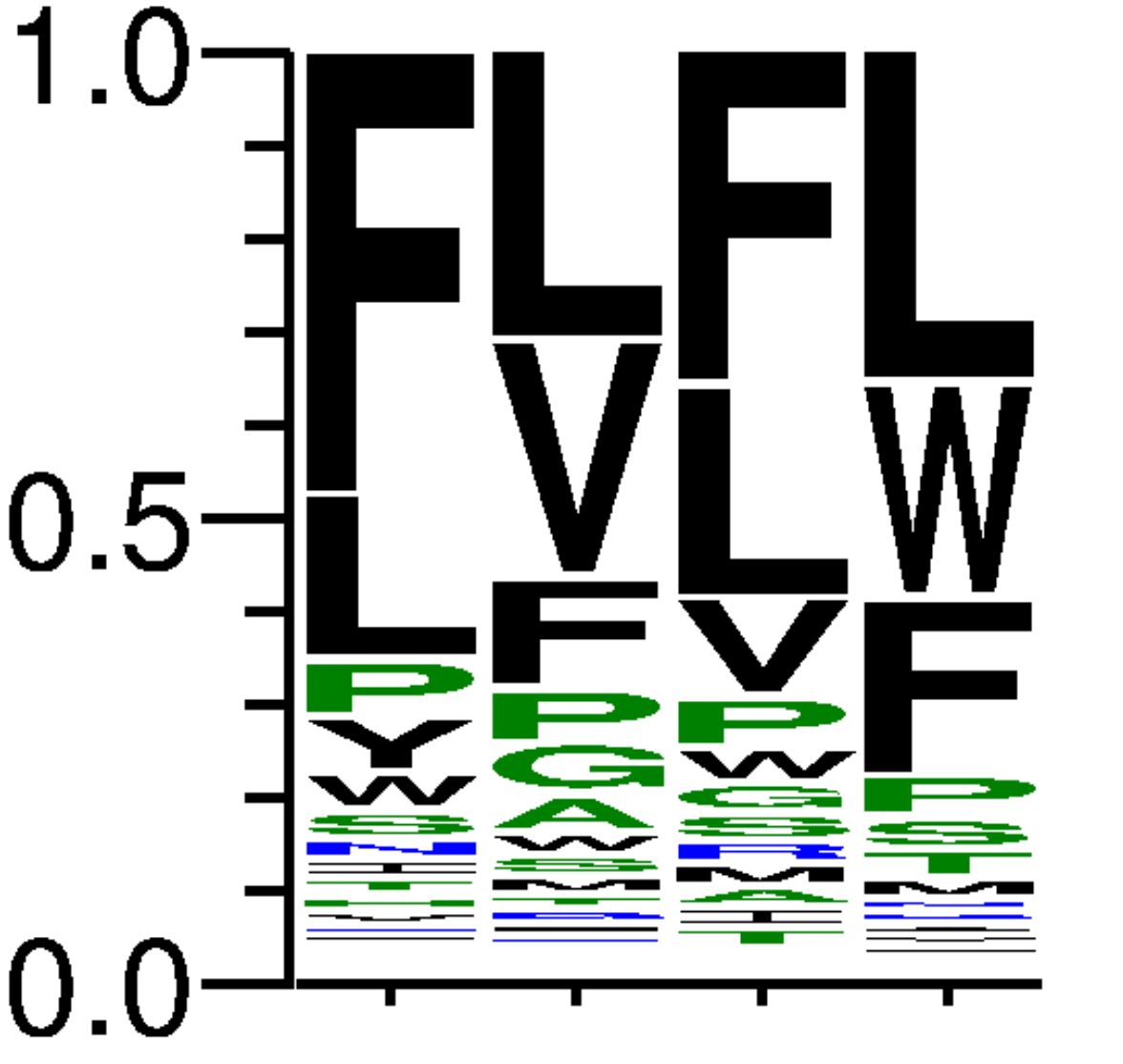 |
| 24 | AGA | 34345 | 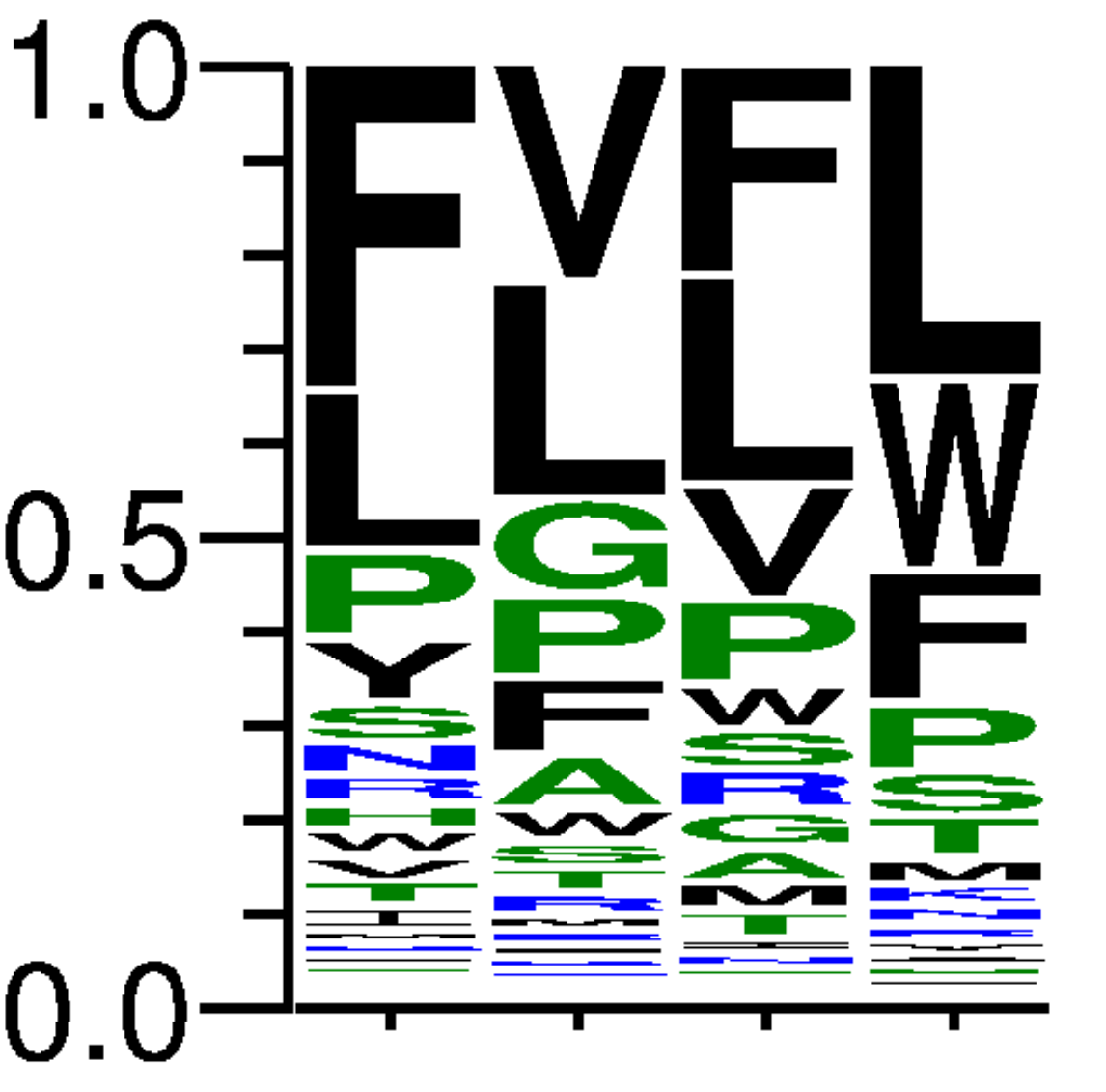 |
| 25 | ATA | 52693 | 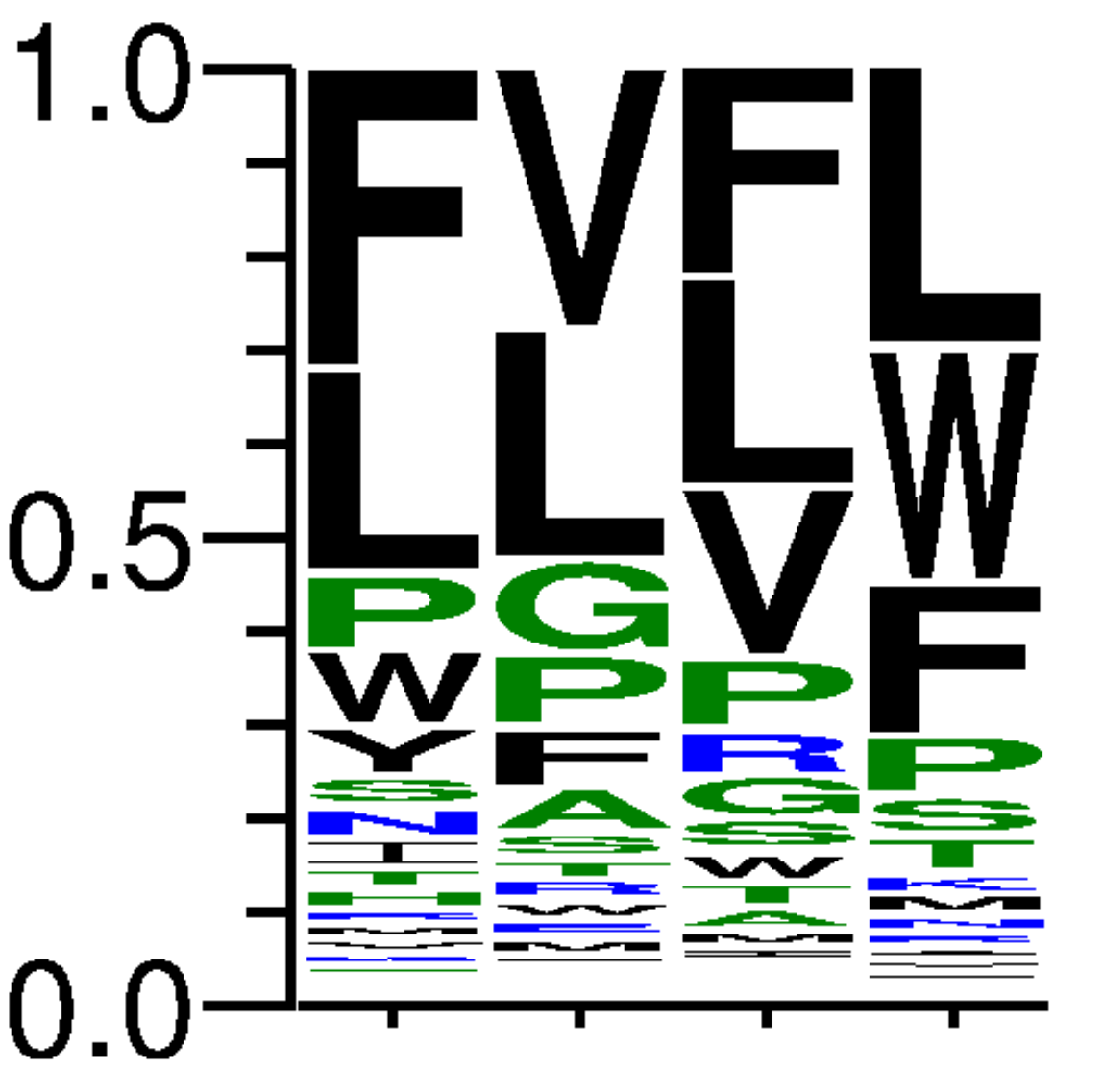 |
| 26 | ACA | 63623 | 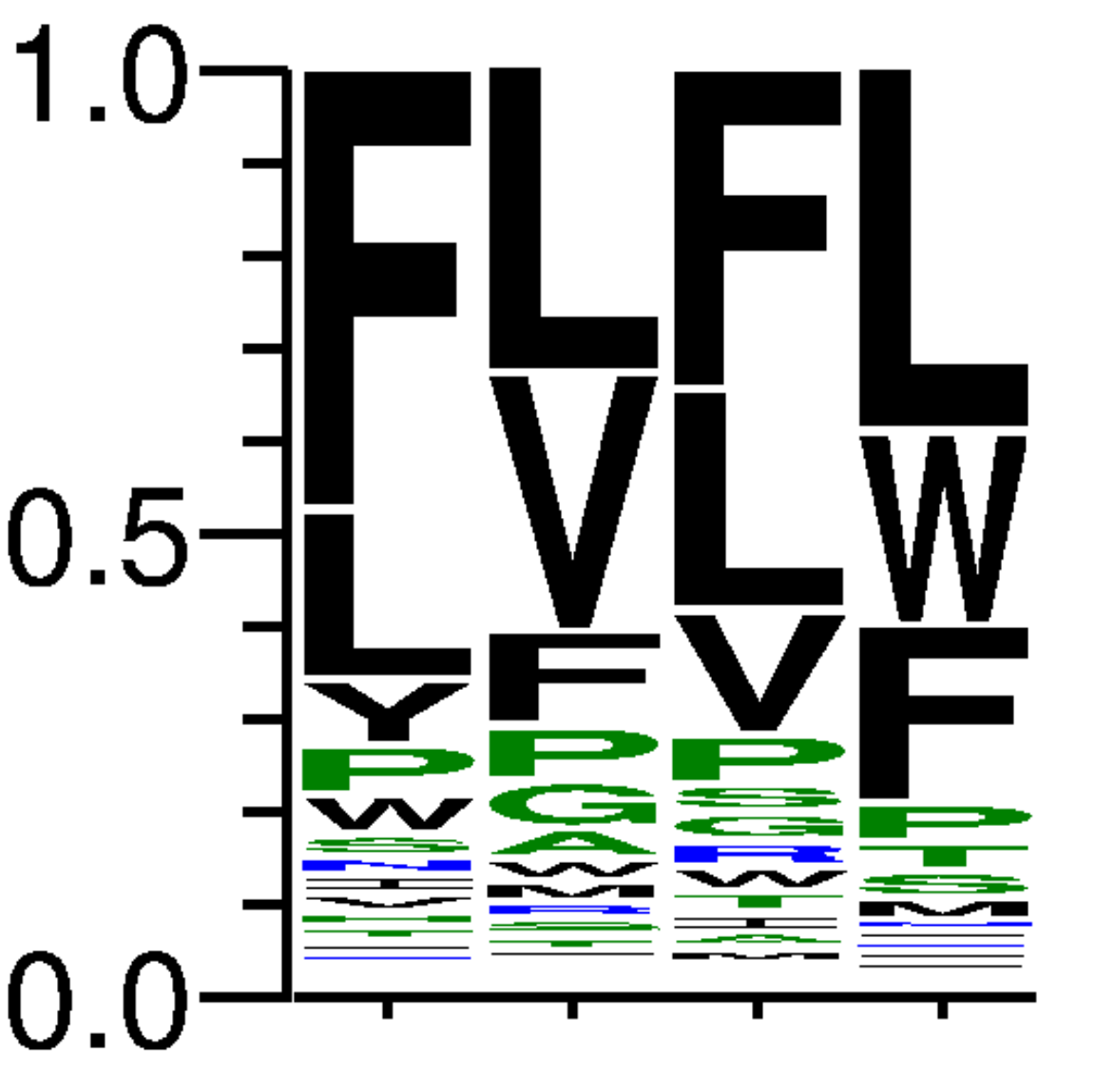 |
| 27 | AGT | 47149 | 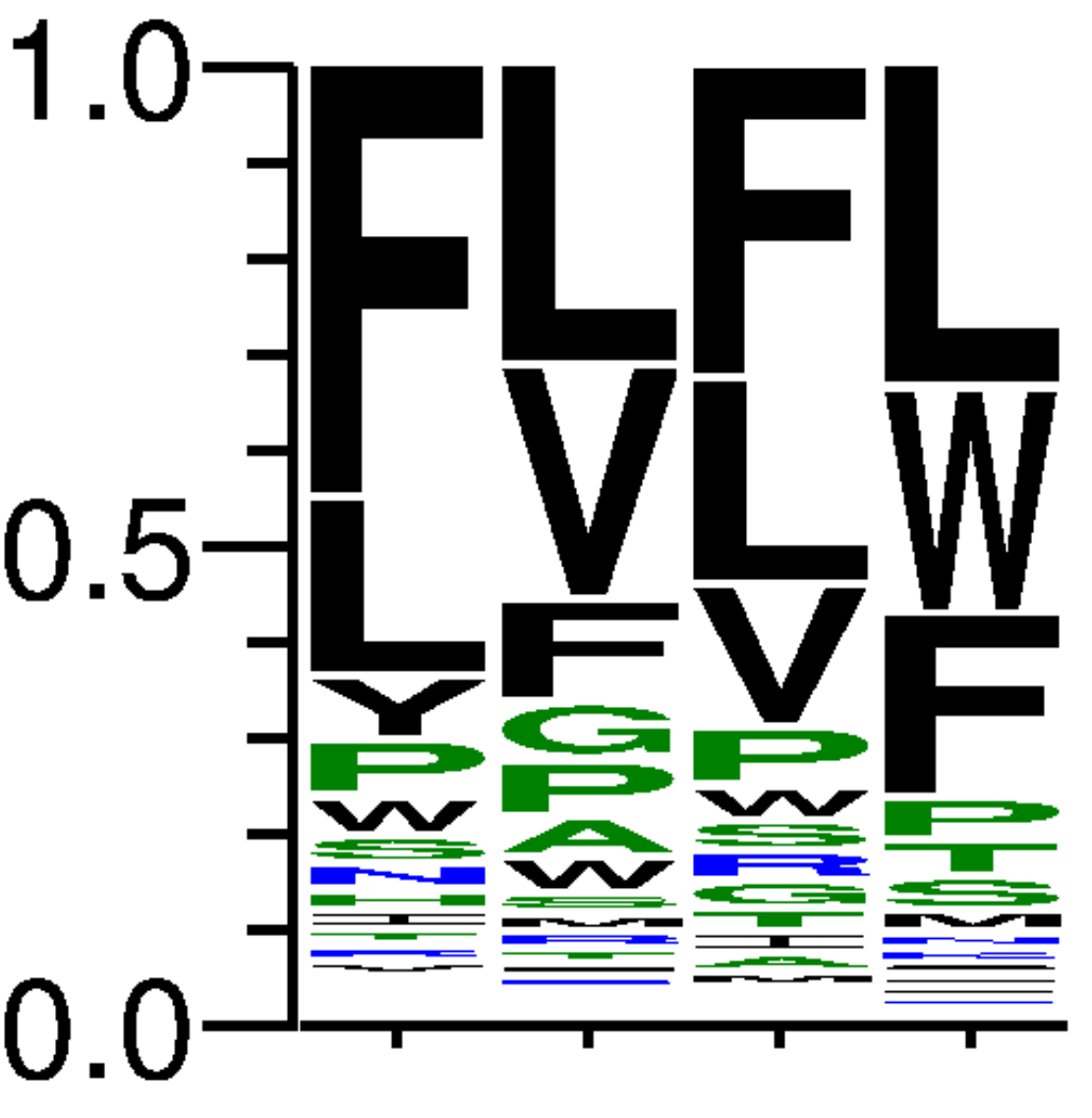 |
| 28 | ATT | 68079 | 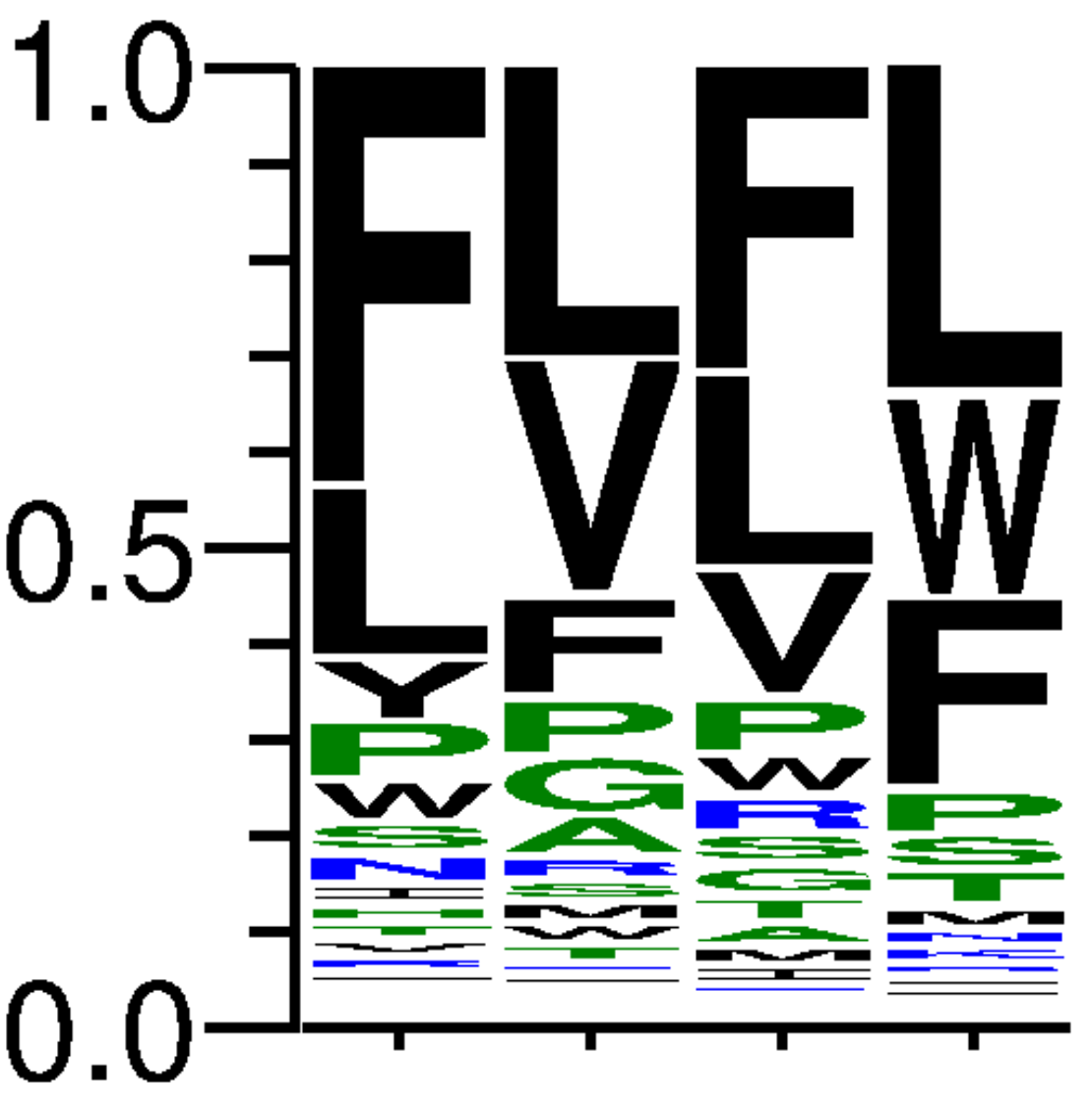 |
| 29 | ACT | 59210 | 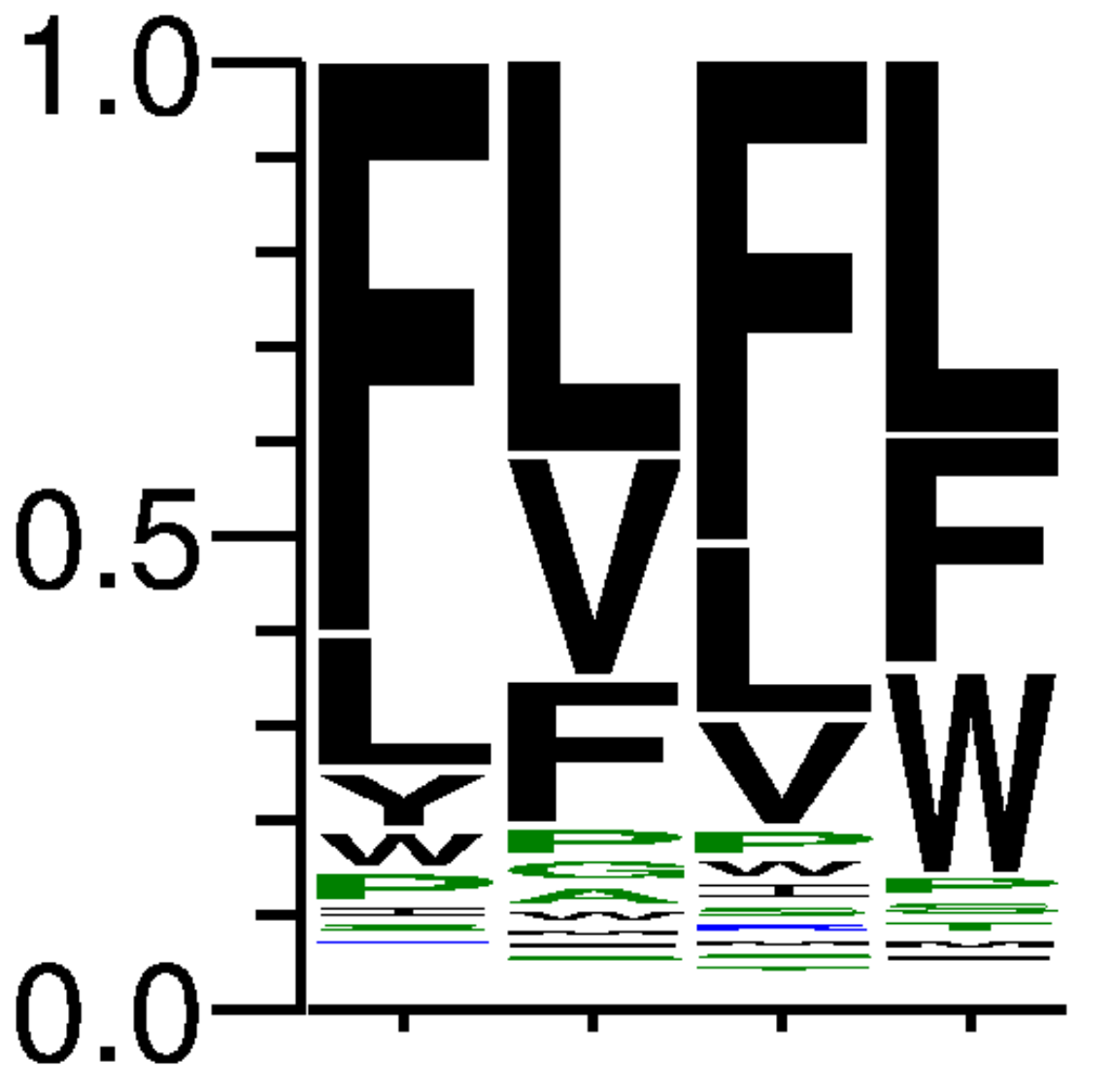 |
| 30 | AGC | 78613 | 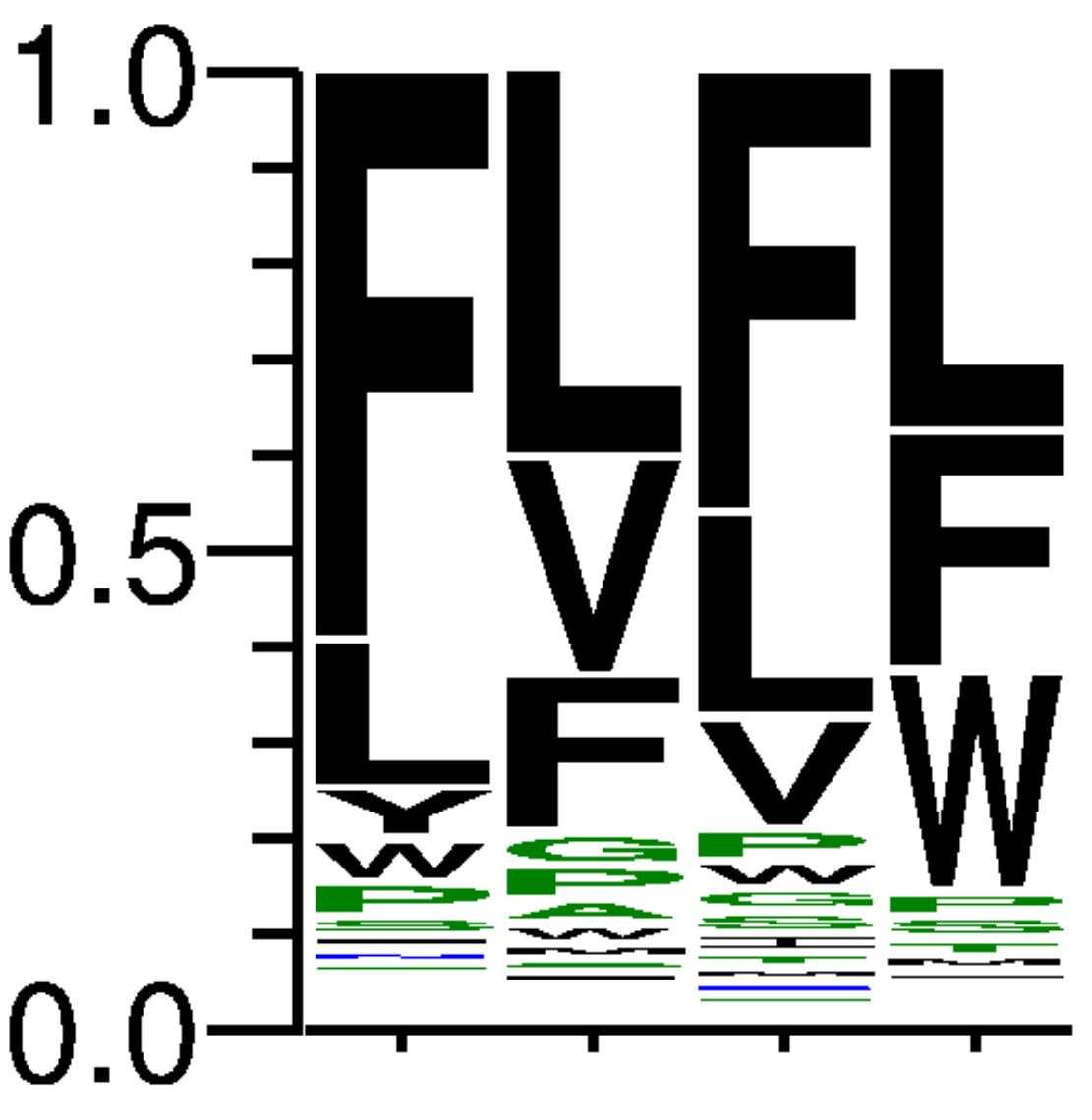 |
| 31 | ATC | 55096 | 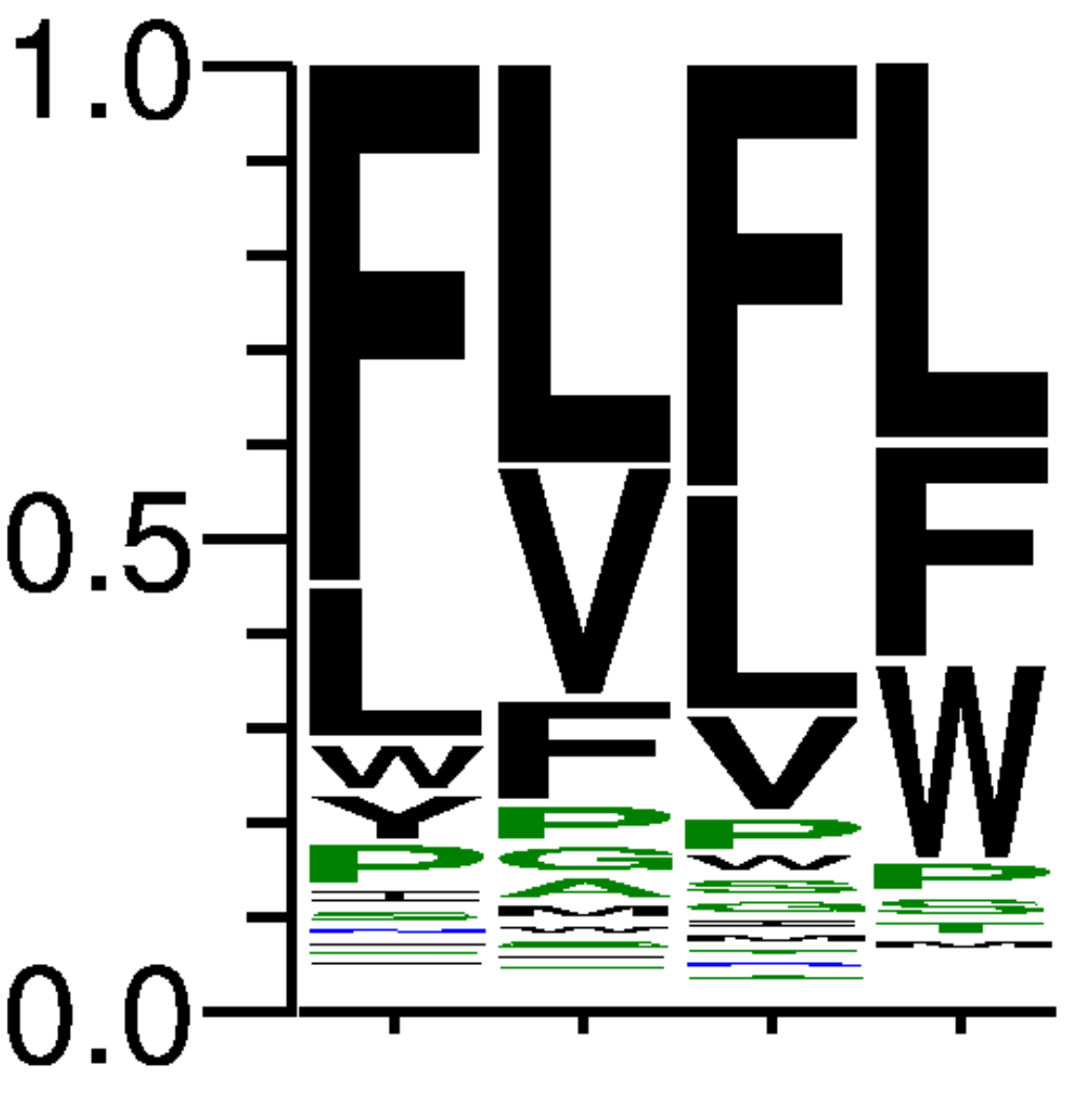 |
| 32 | ACC | 45545 | 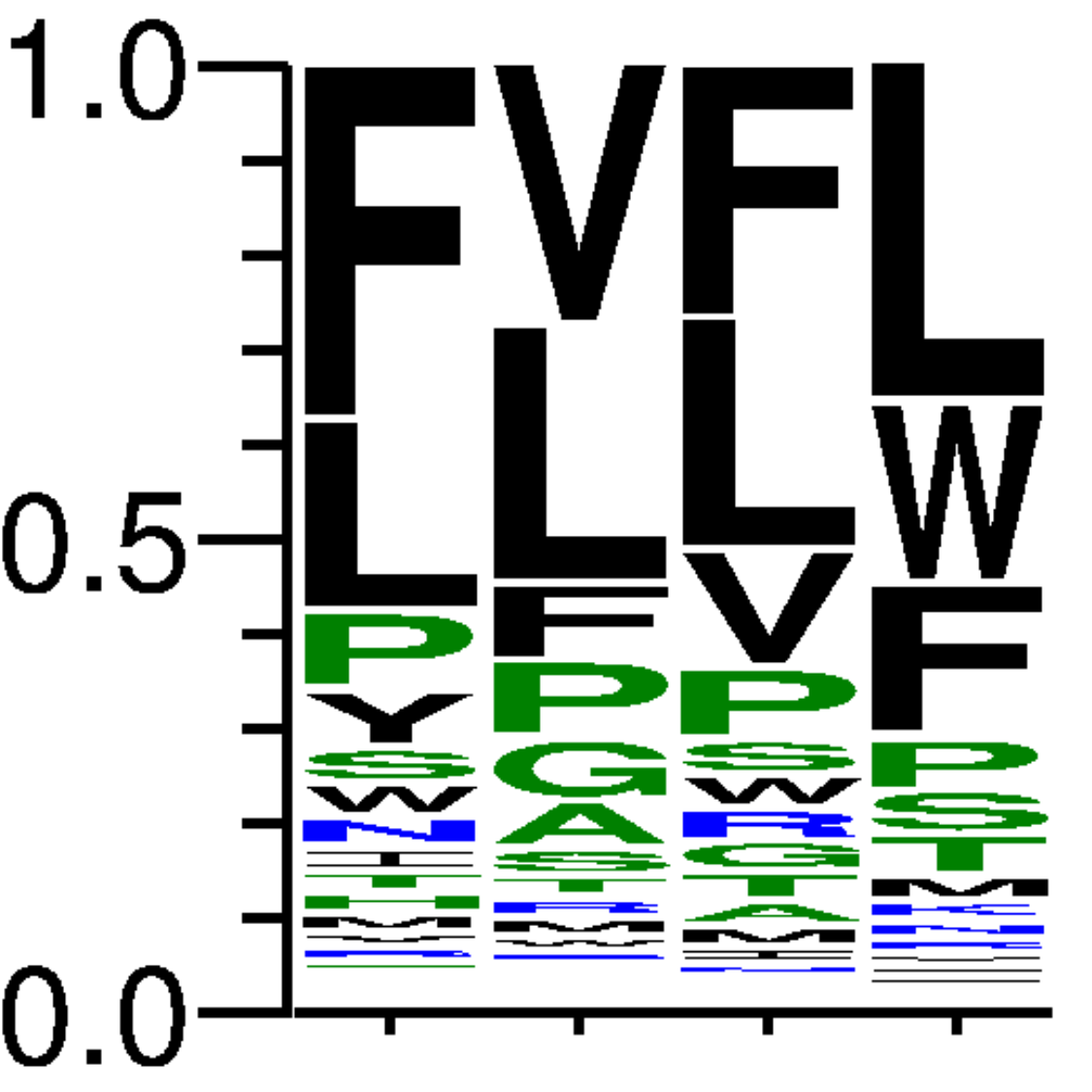 |
| 33 | TTG | 41691 | 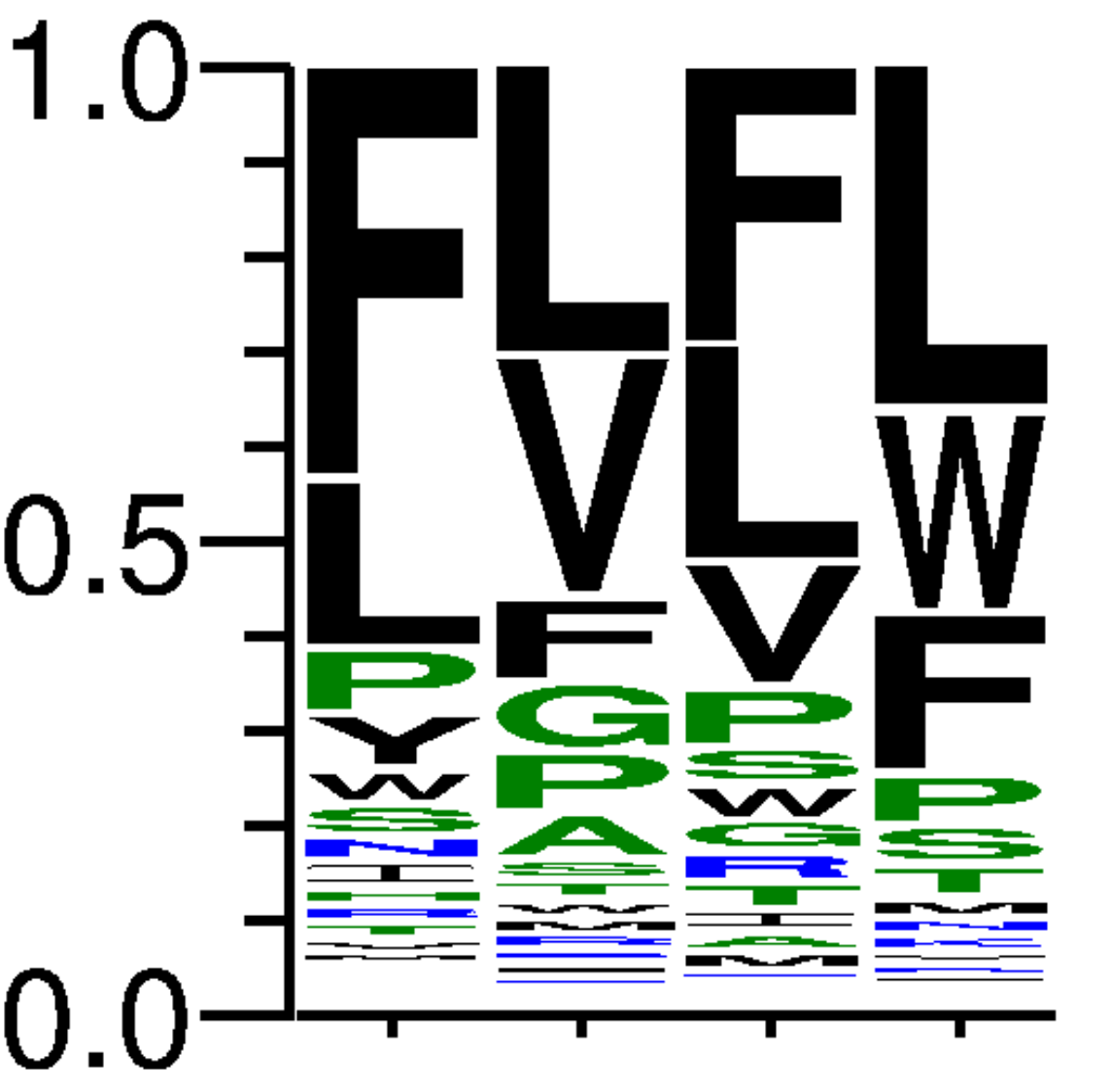 |
| 34 | TTA | 43464 | 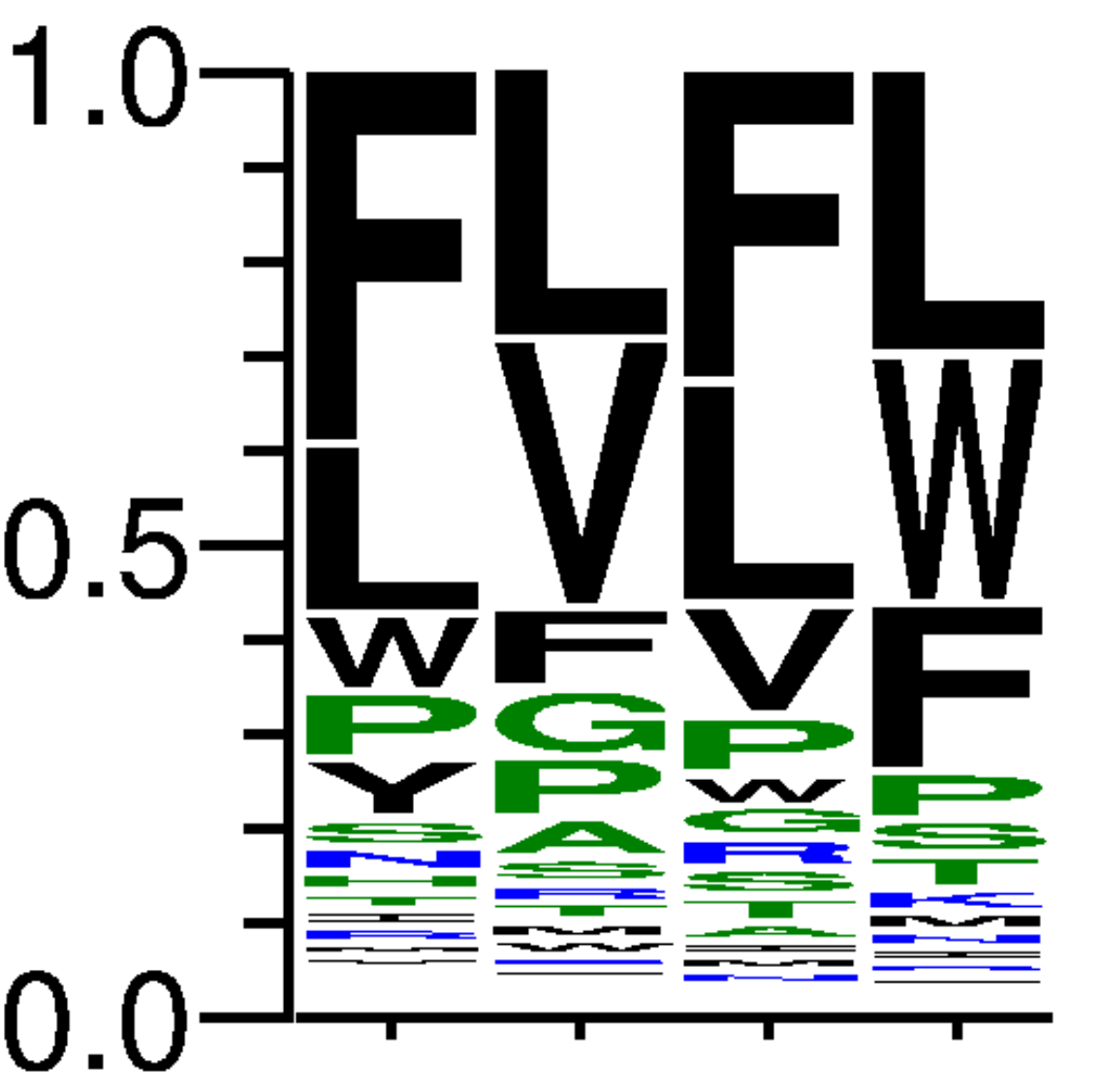 |
| 35 | TTT | 69425 | 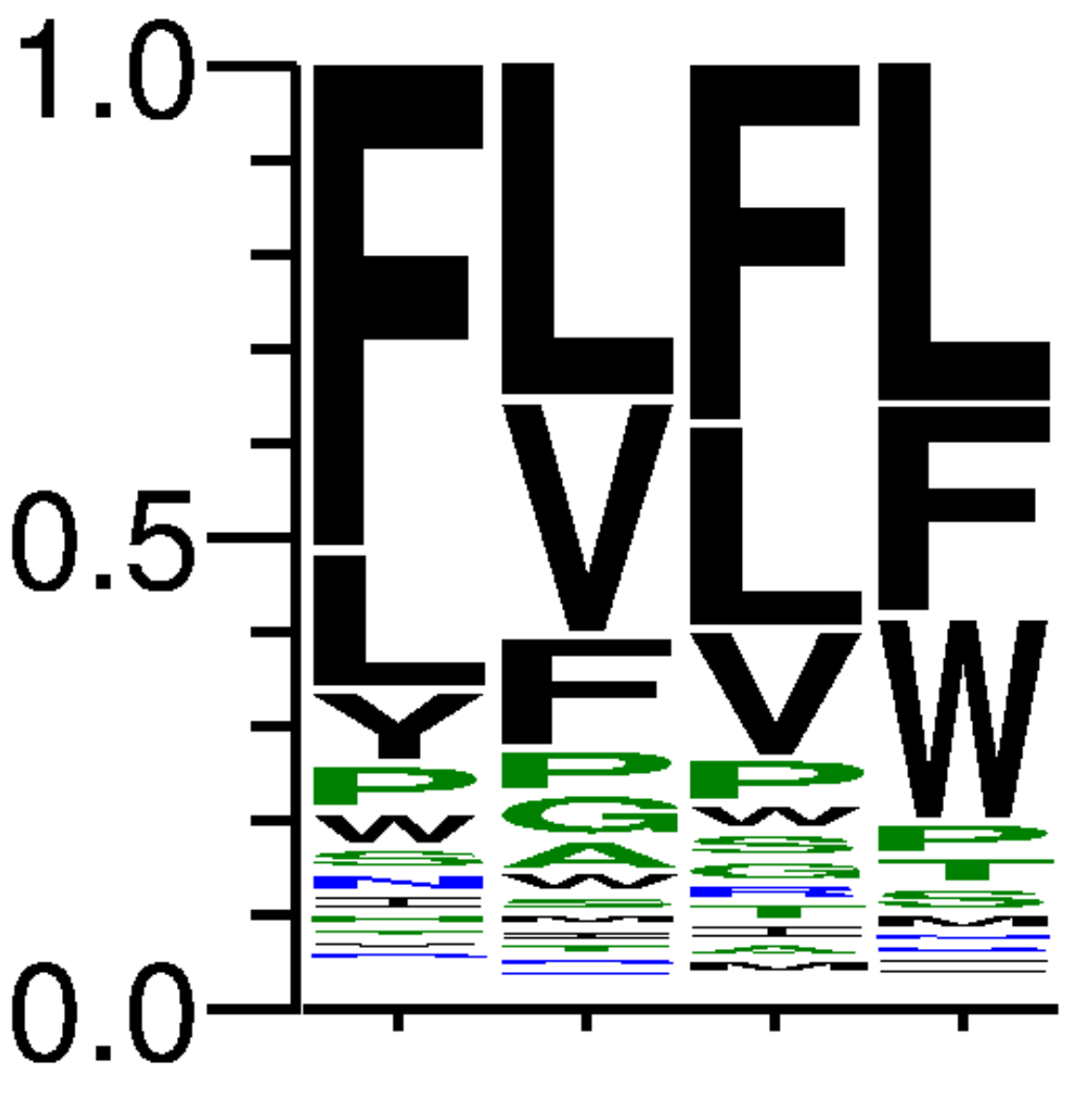 |
| 36 | TTC | 46208 | 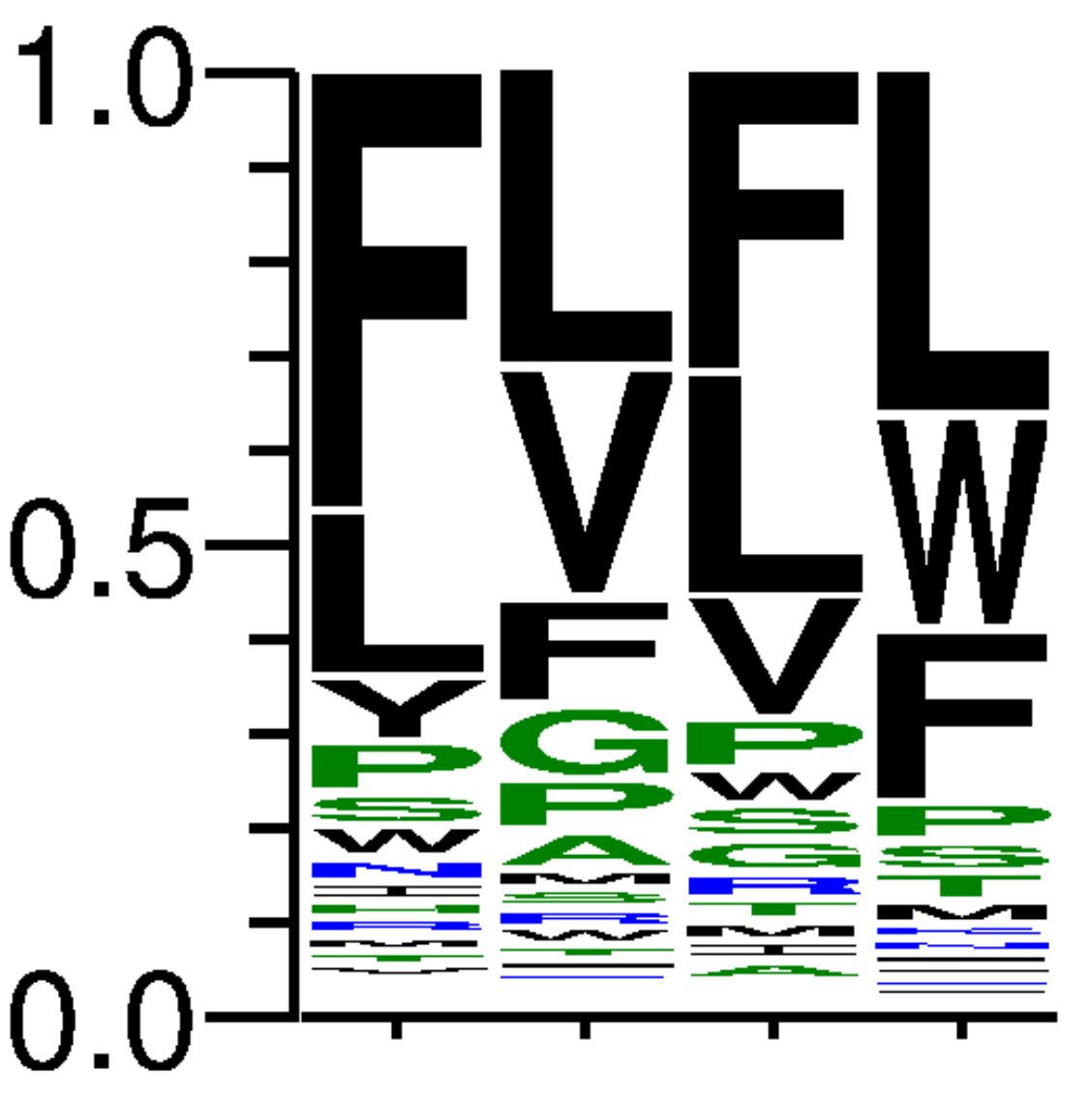 |
| 37 | TGG | 152133 | 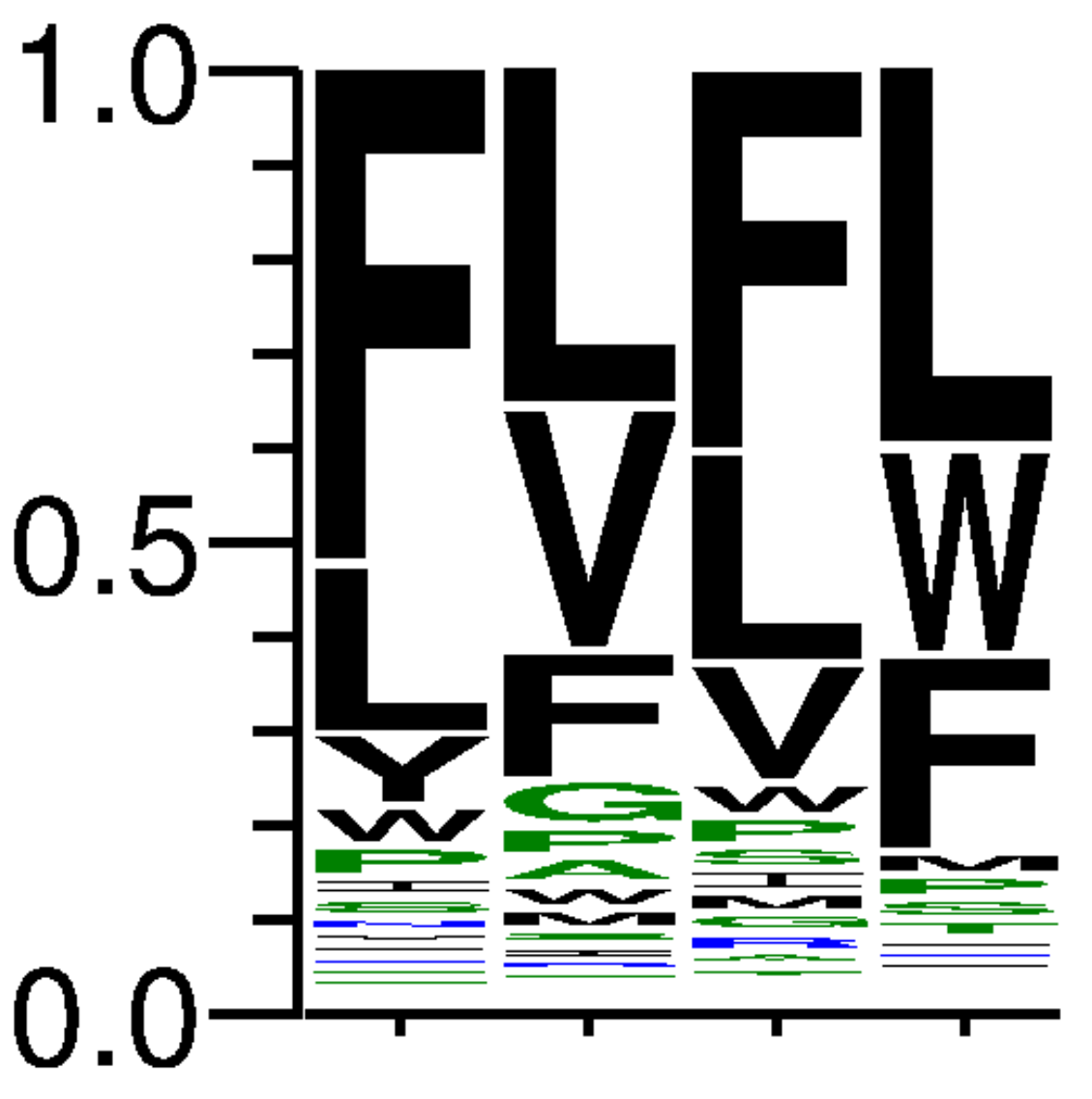 |
| 38 | TAG | 55822 | 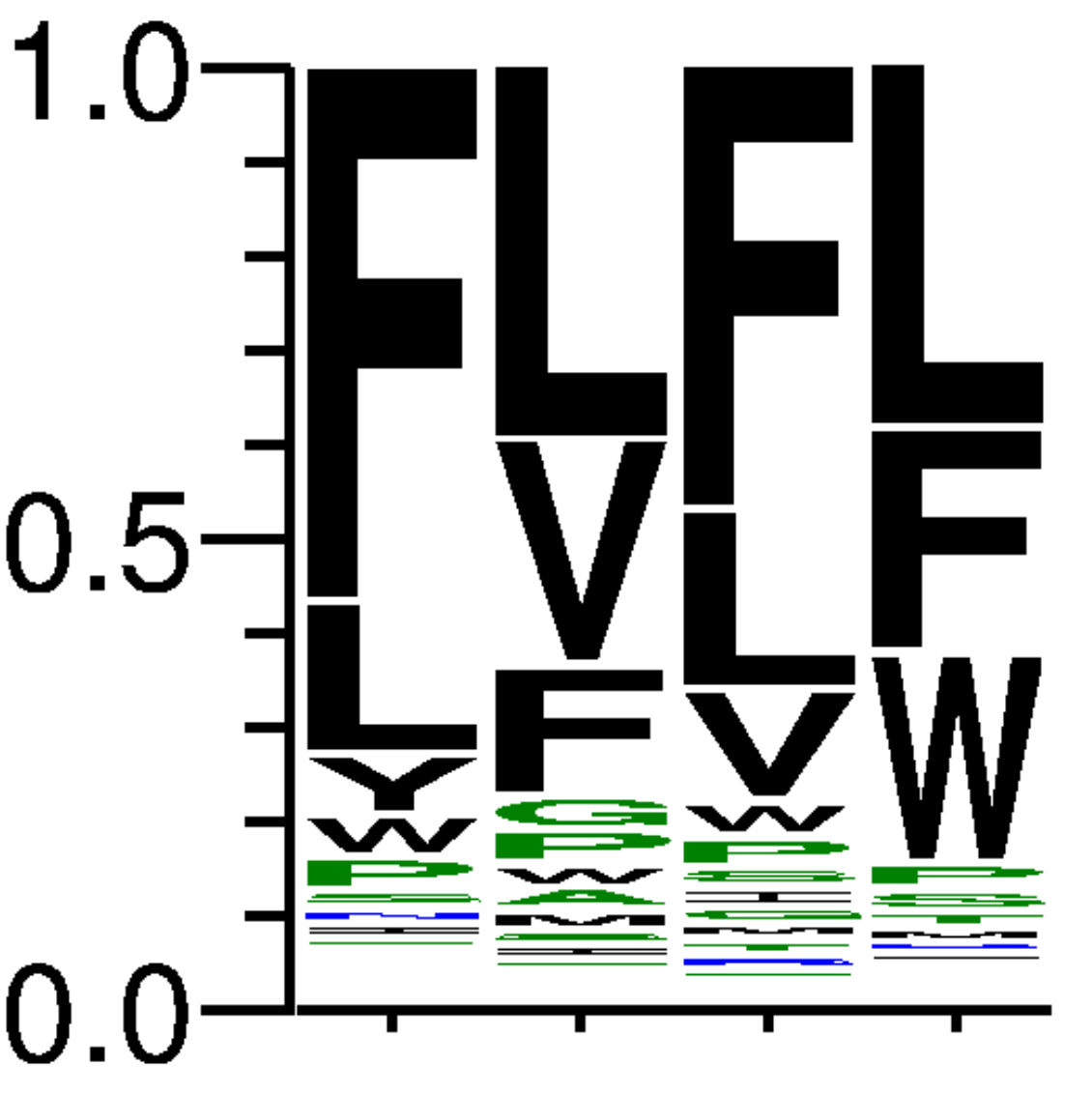 |
| 39 | TCG | 40287 | 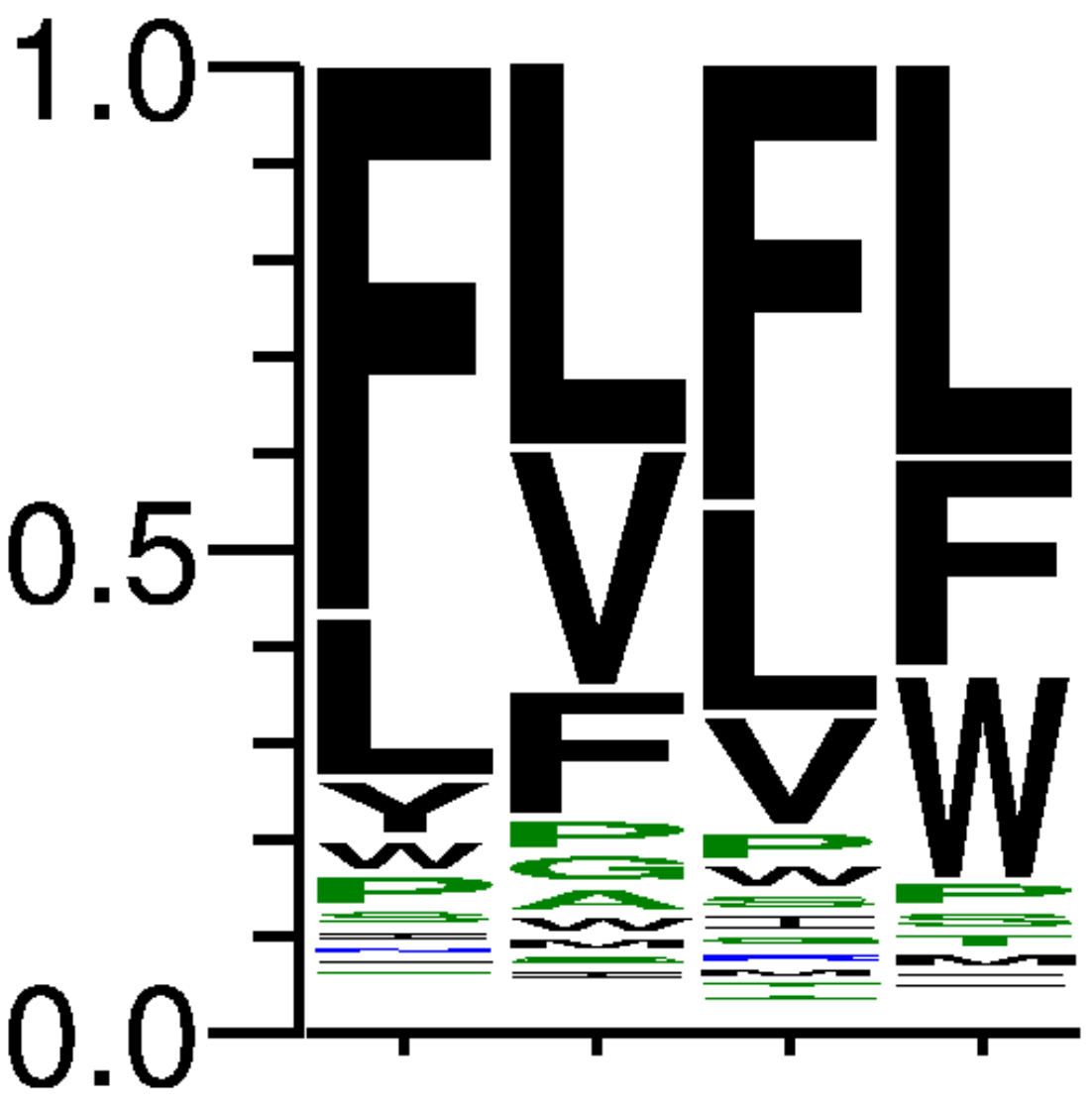 |
| 40 | TGA | 100686 | 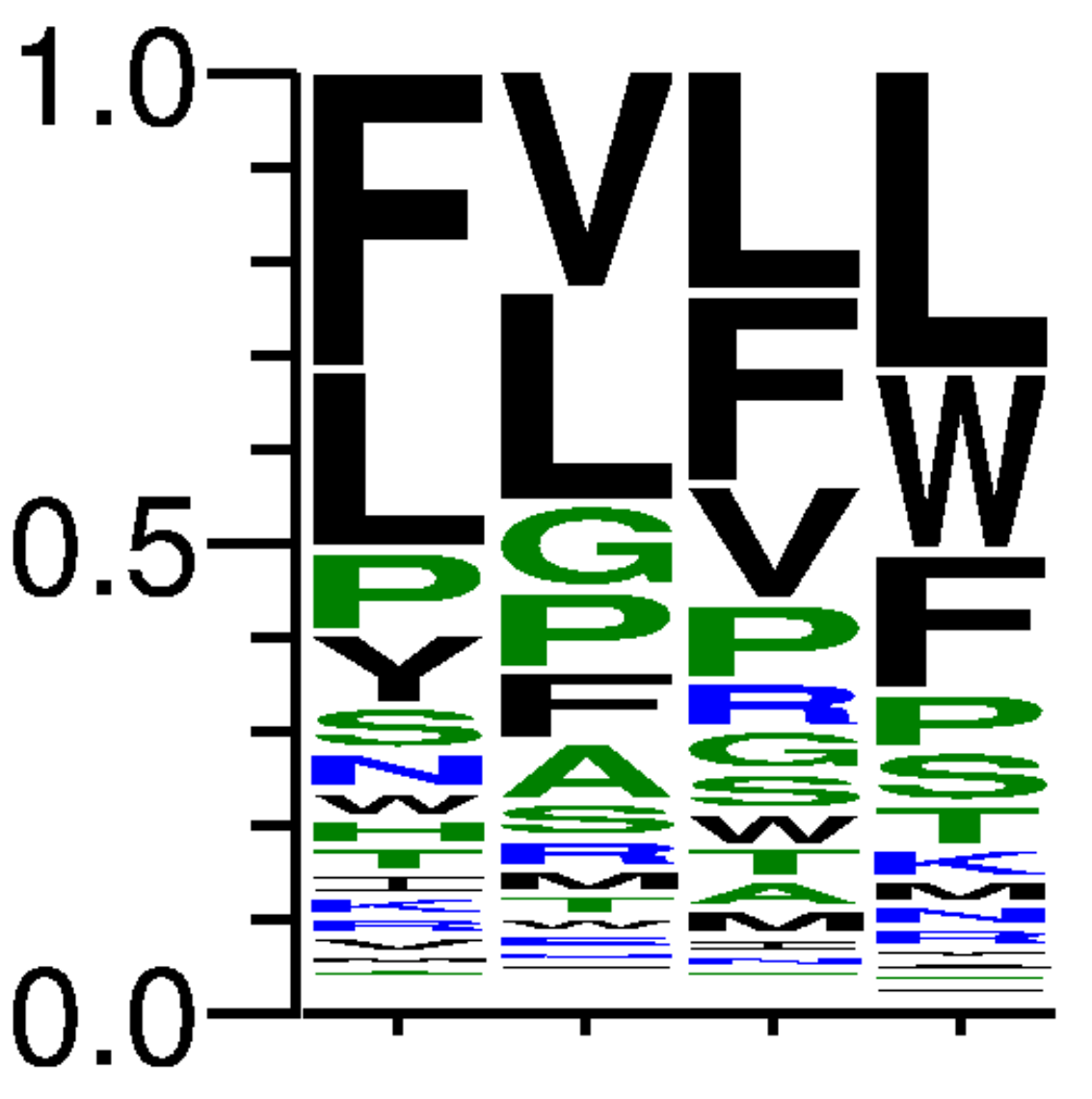 |
| 41 | TAA | 87310 | 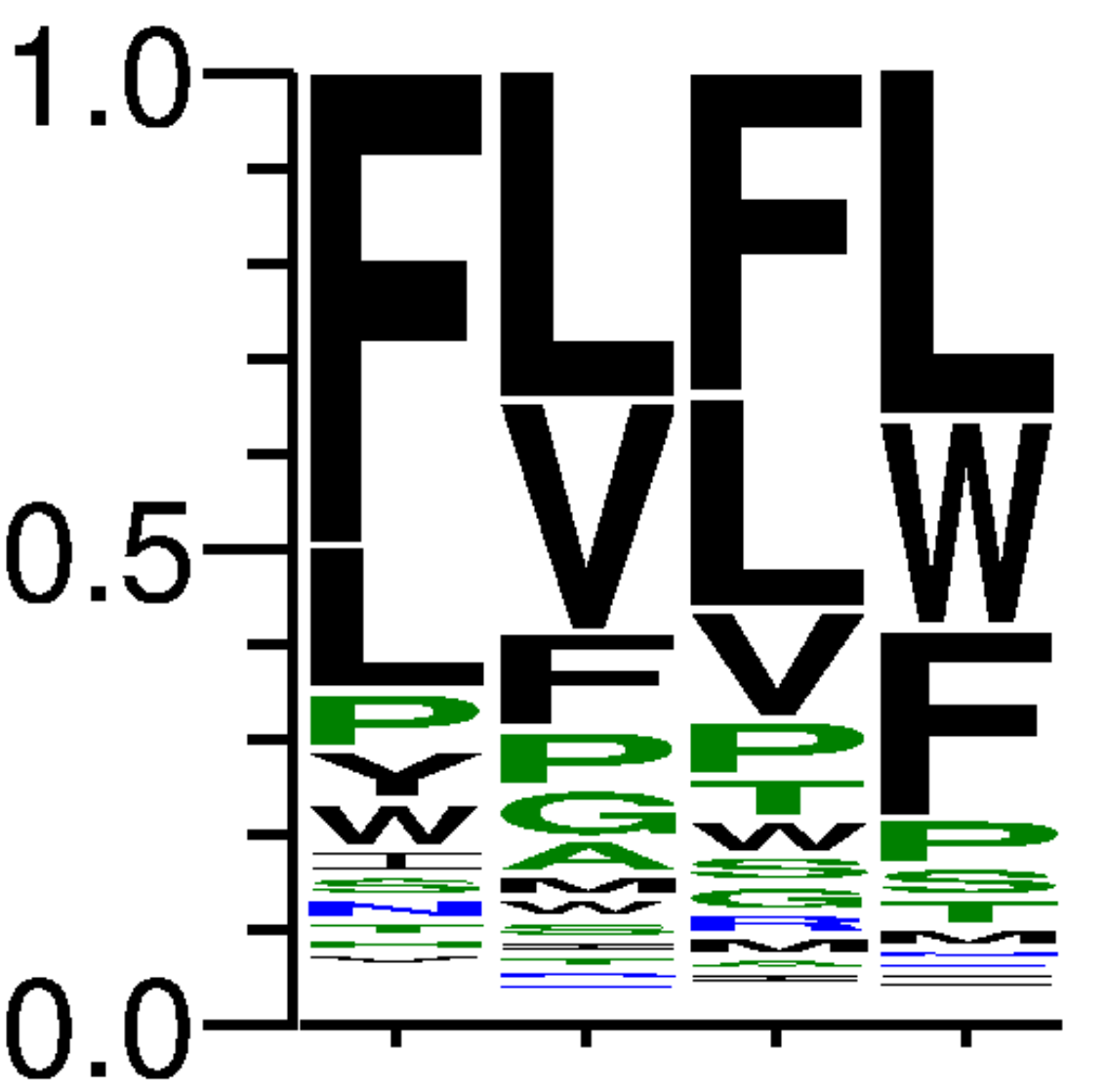 |
| 42 | TCA | 38444 | 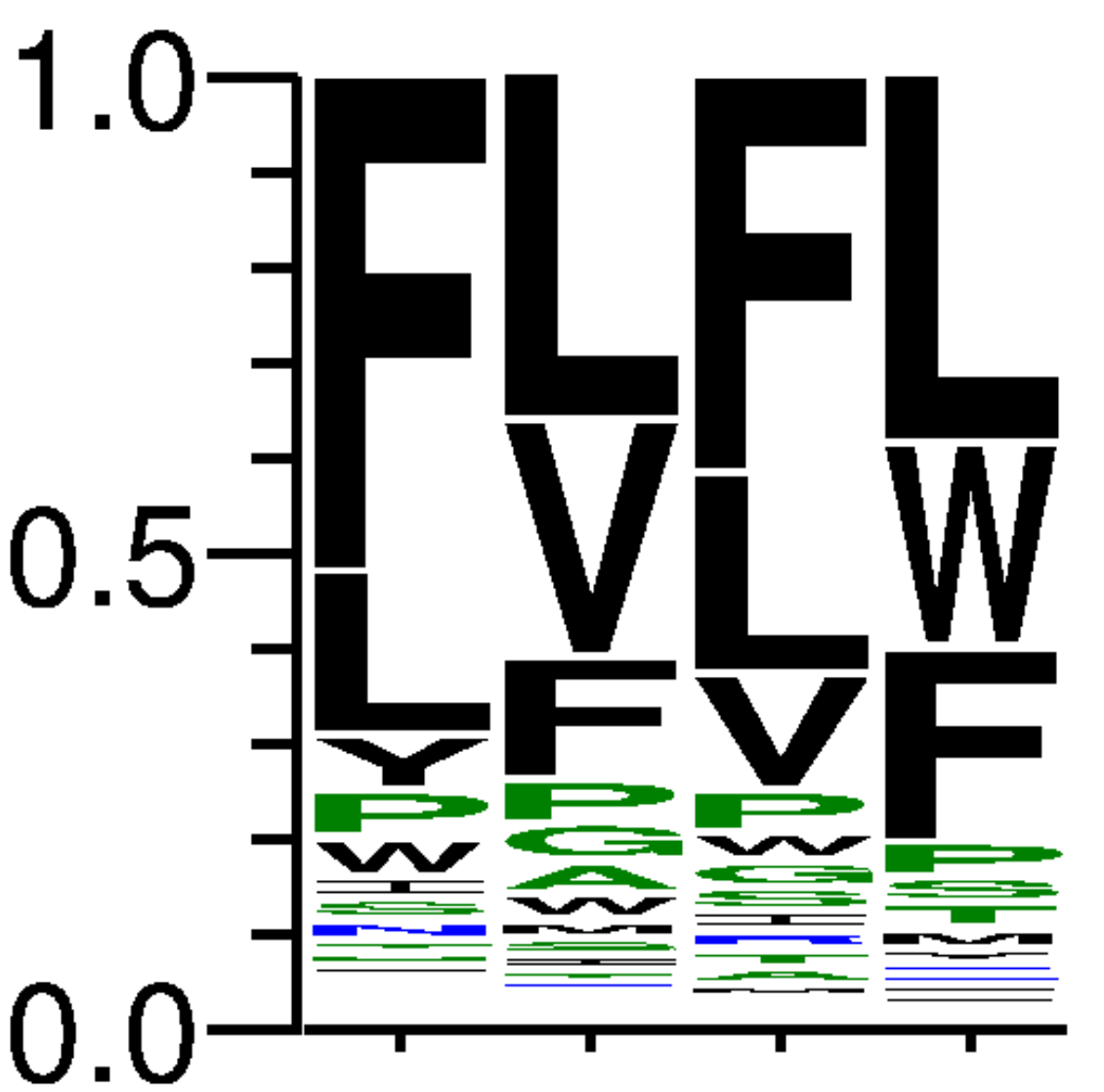 |
| 43 | TGT | 100767 | 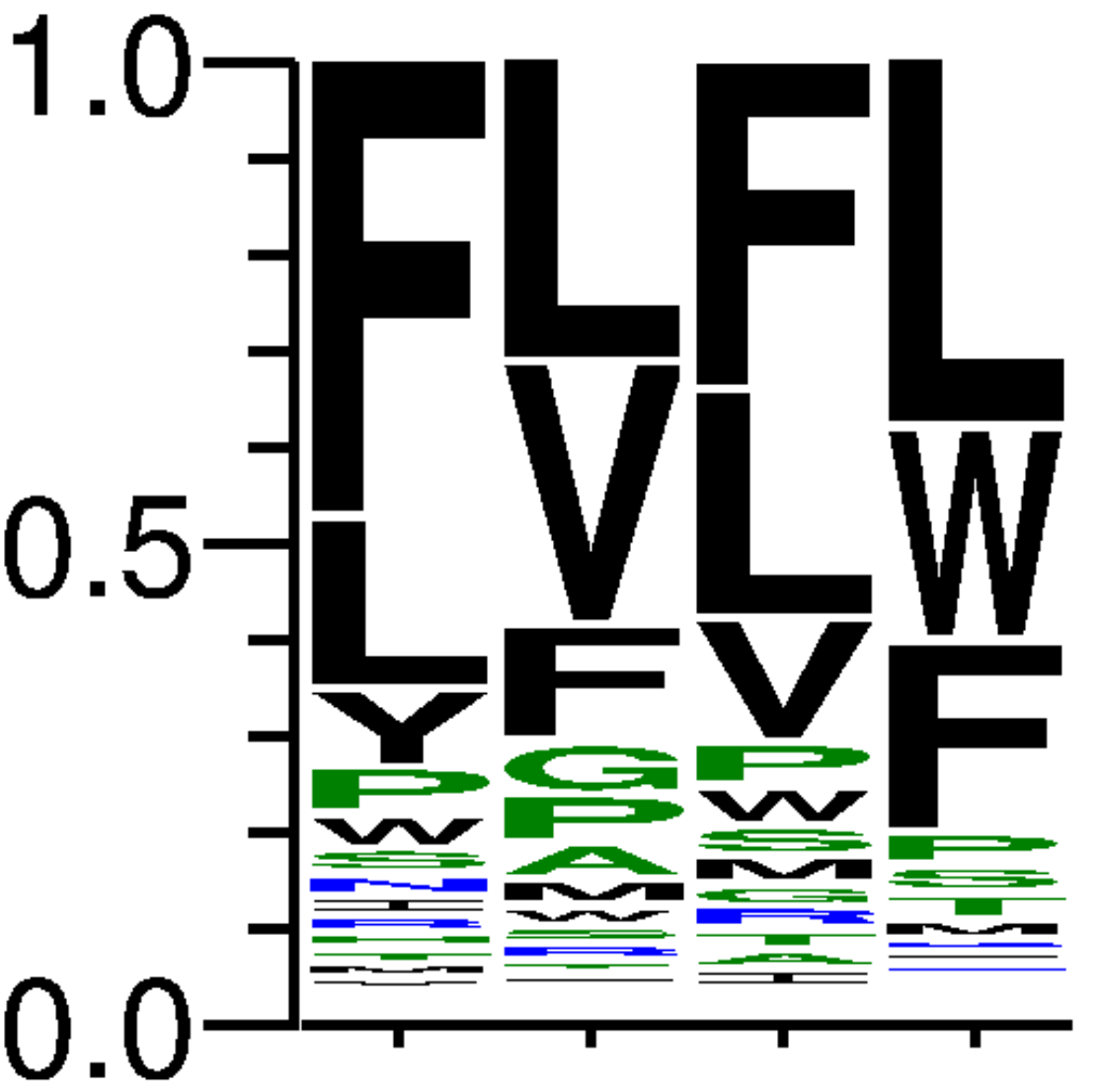 |
| 44 | TAT | 74930 | 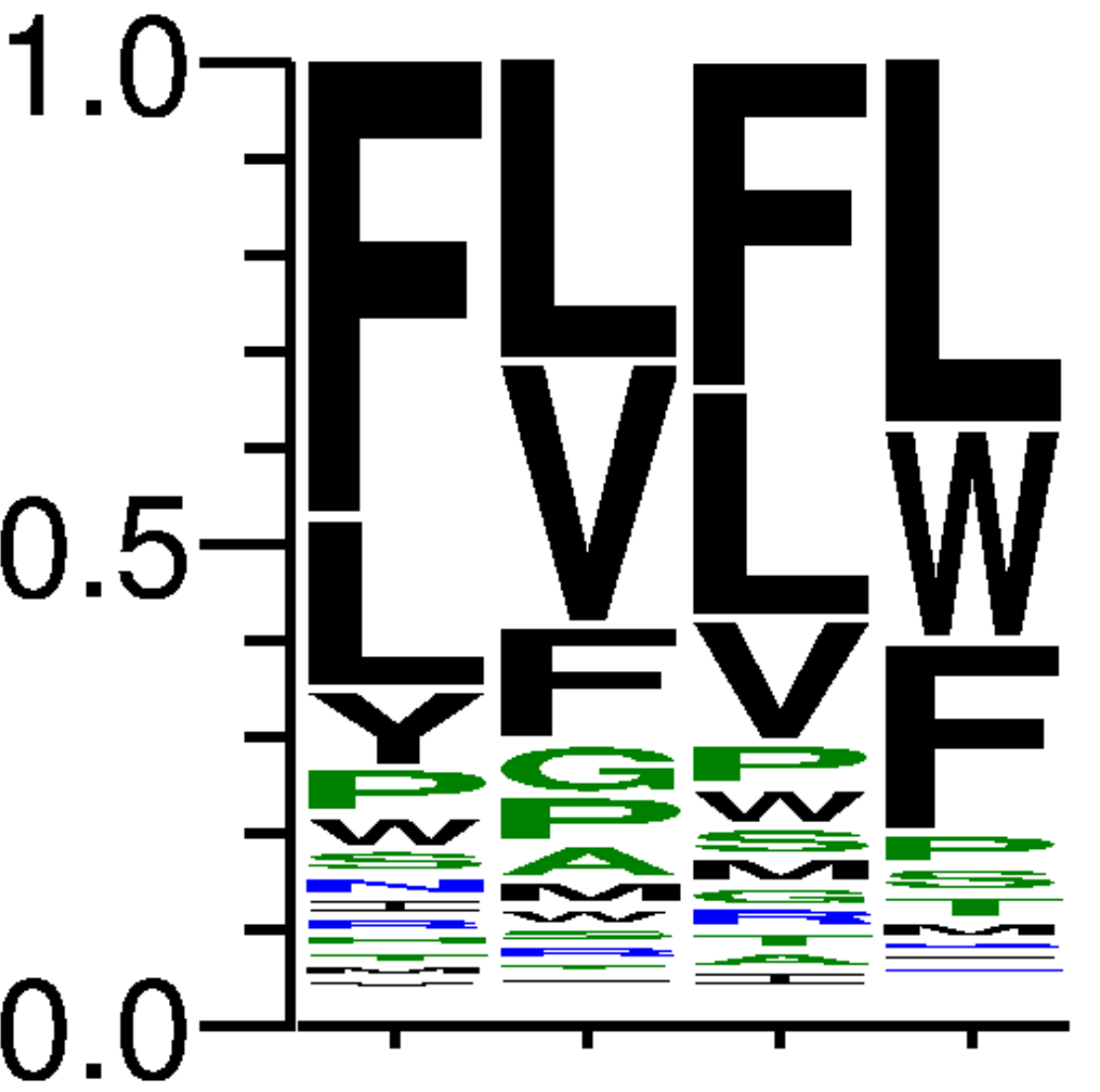 |
| 45 | TCT | 59807 | 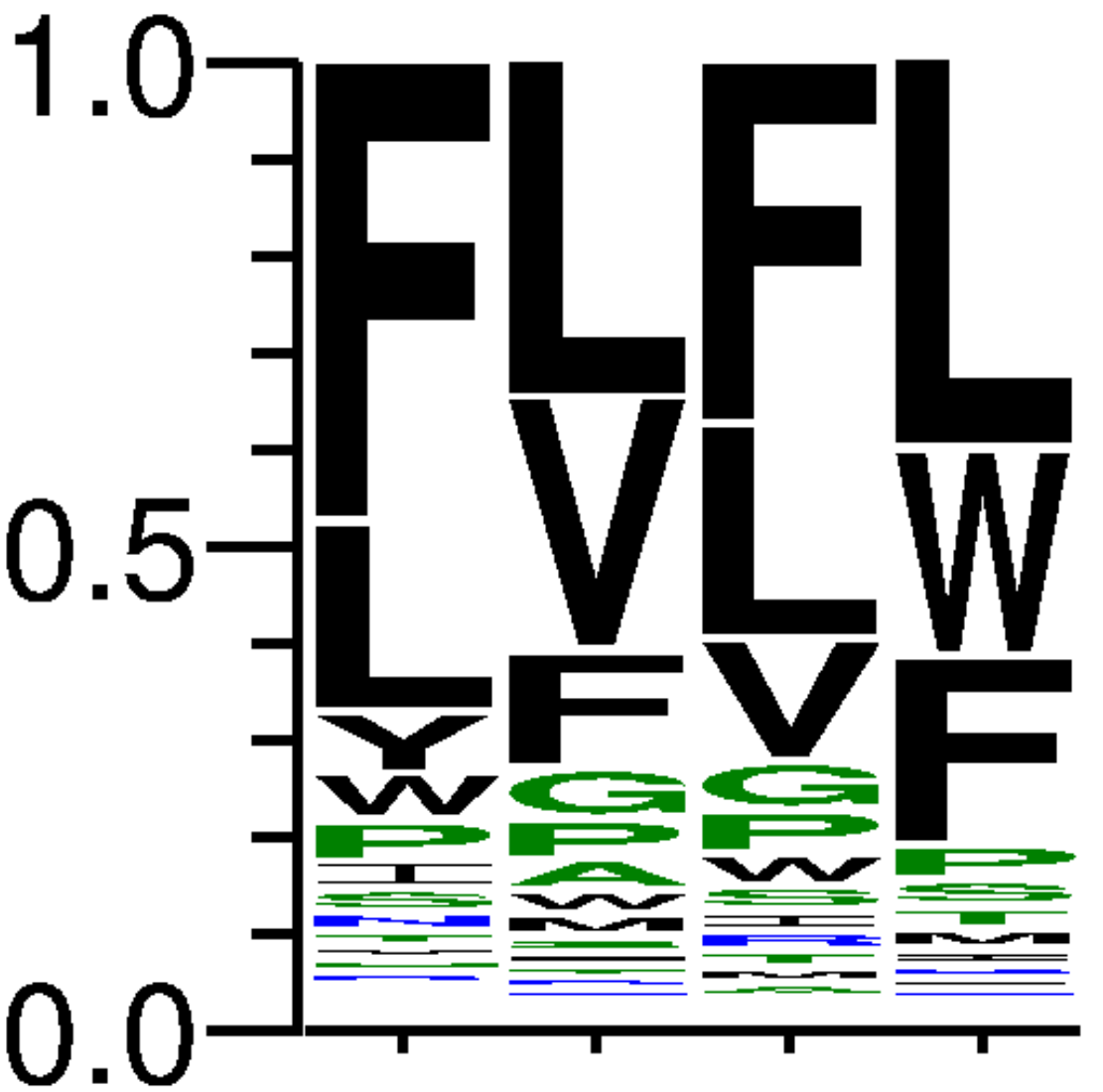 |
| 46 | TGC | 76625 | 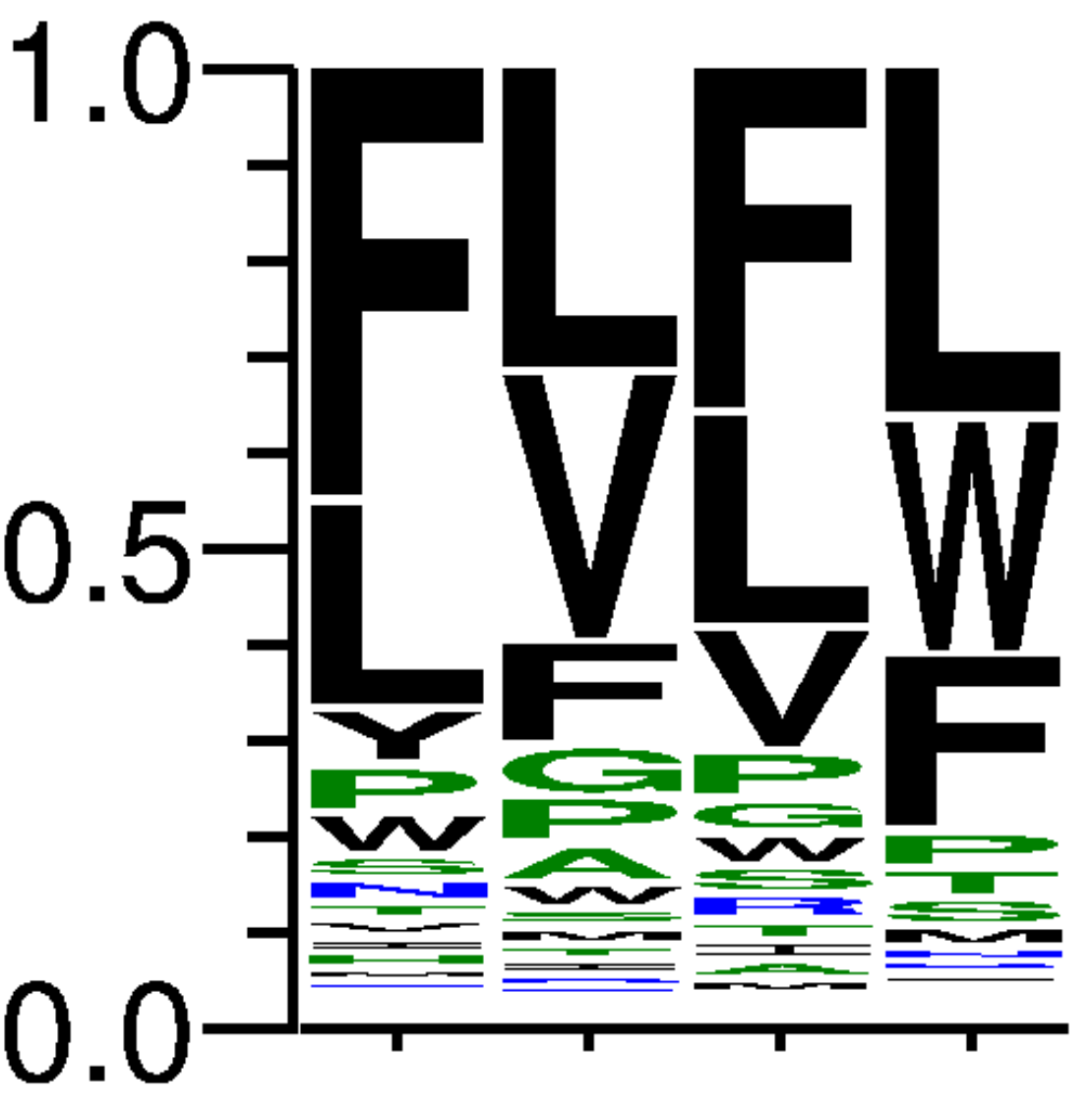 |
| 47 | TAC | 63637 | 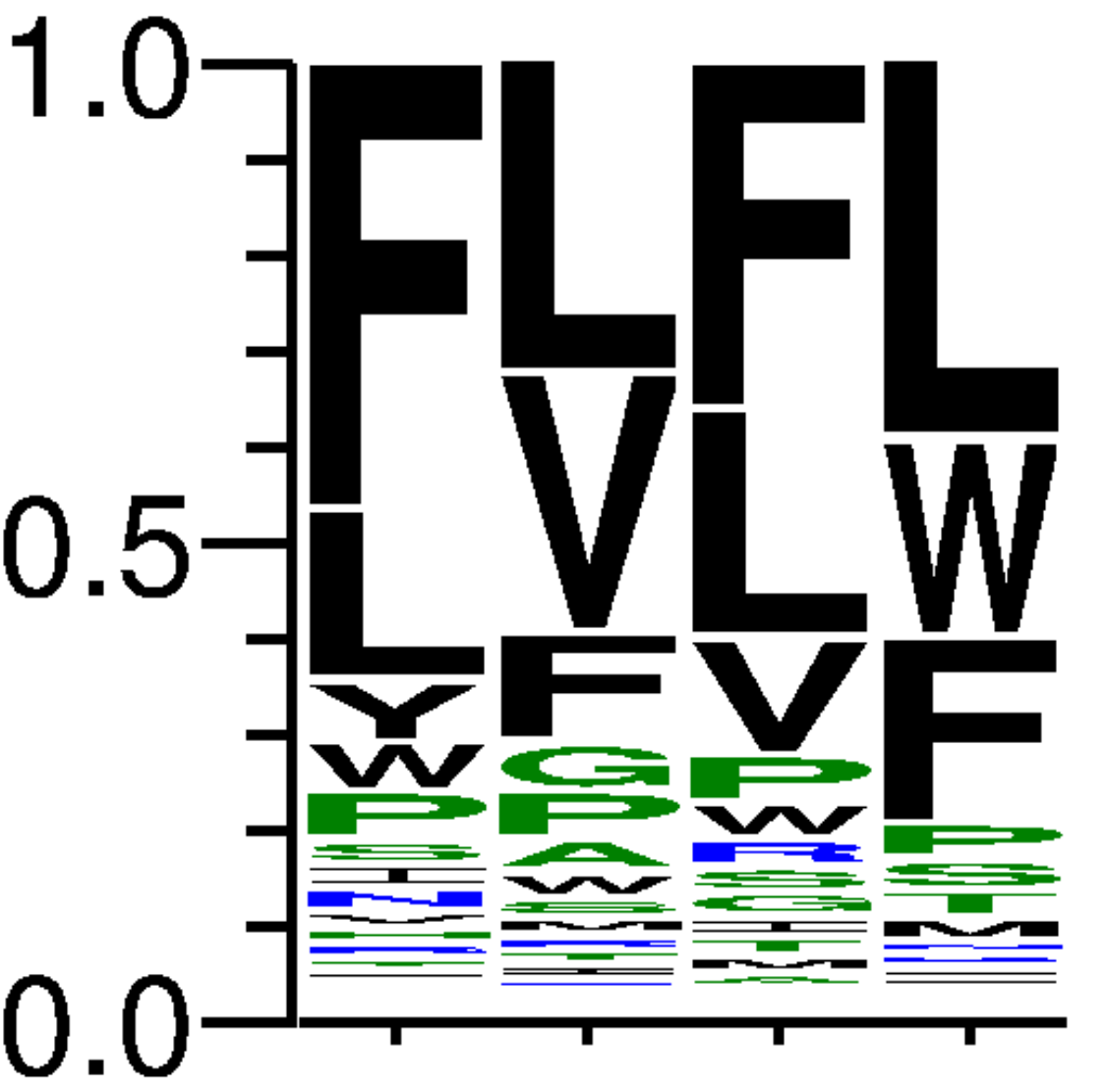 |
| 48 | TCC | 32919 | 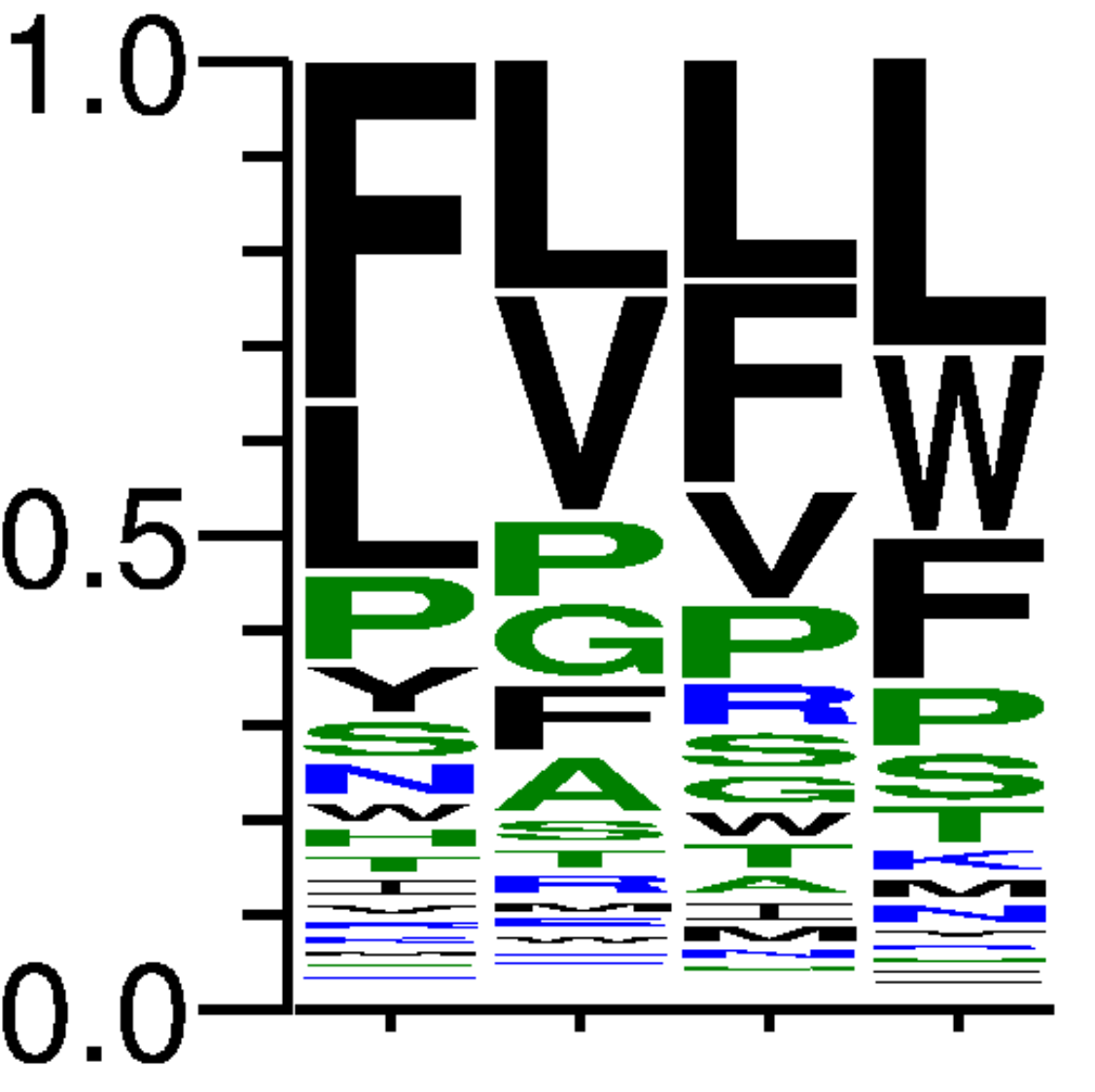 |
| 49 | CCG | 105741 | 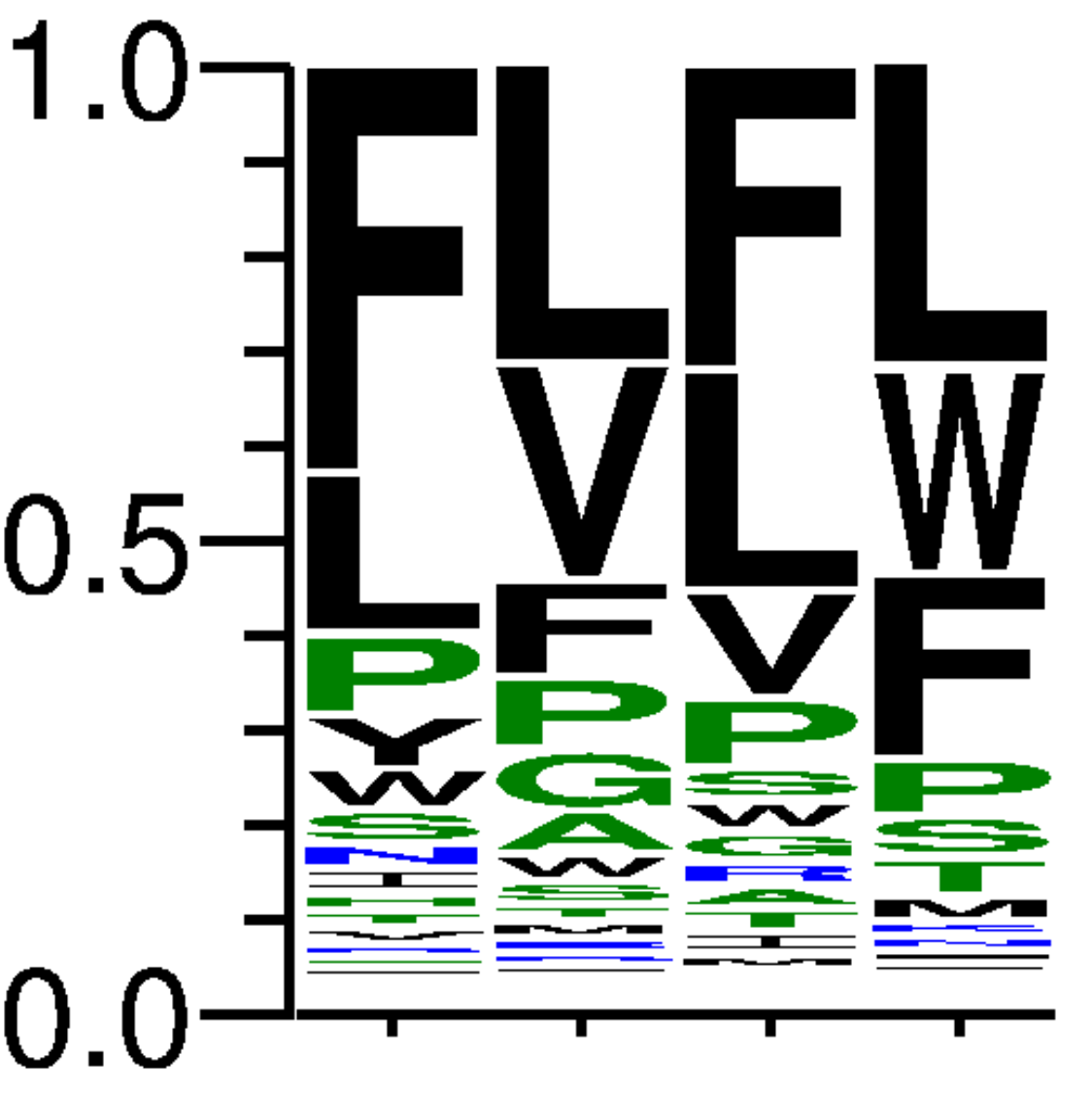 |
| 50 | CCA | 144997 | 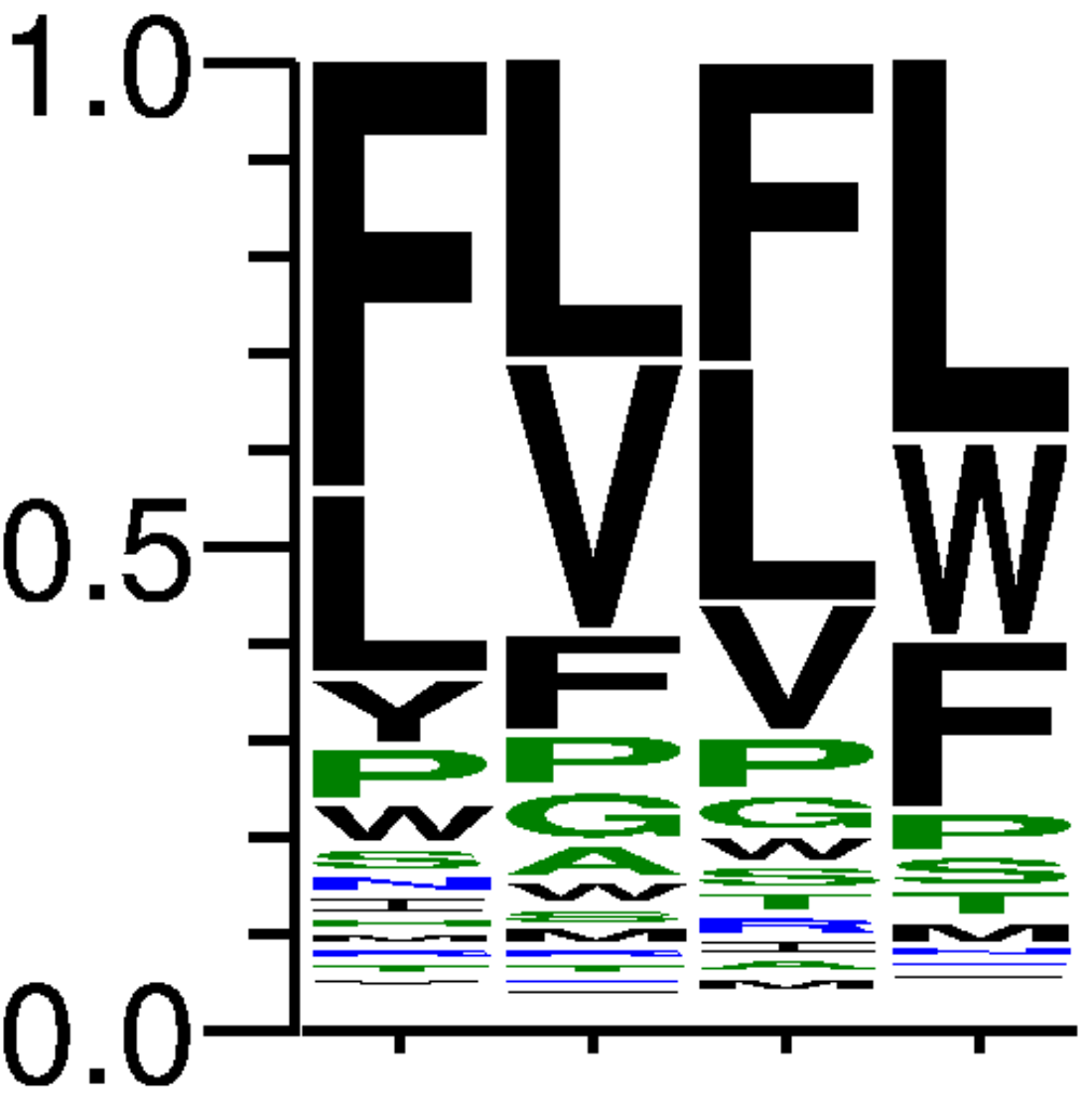 |
| 51 | CCT | 89487 | 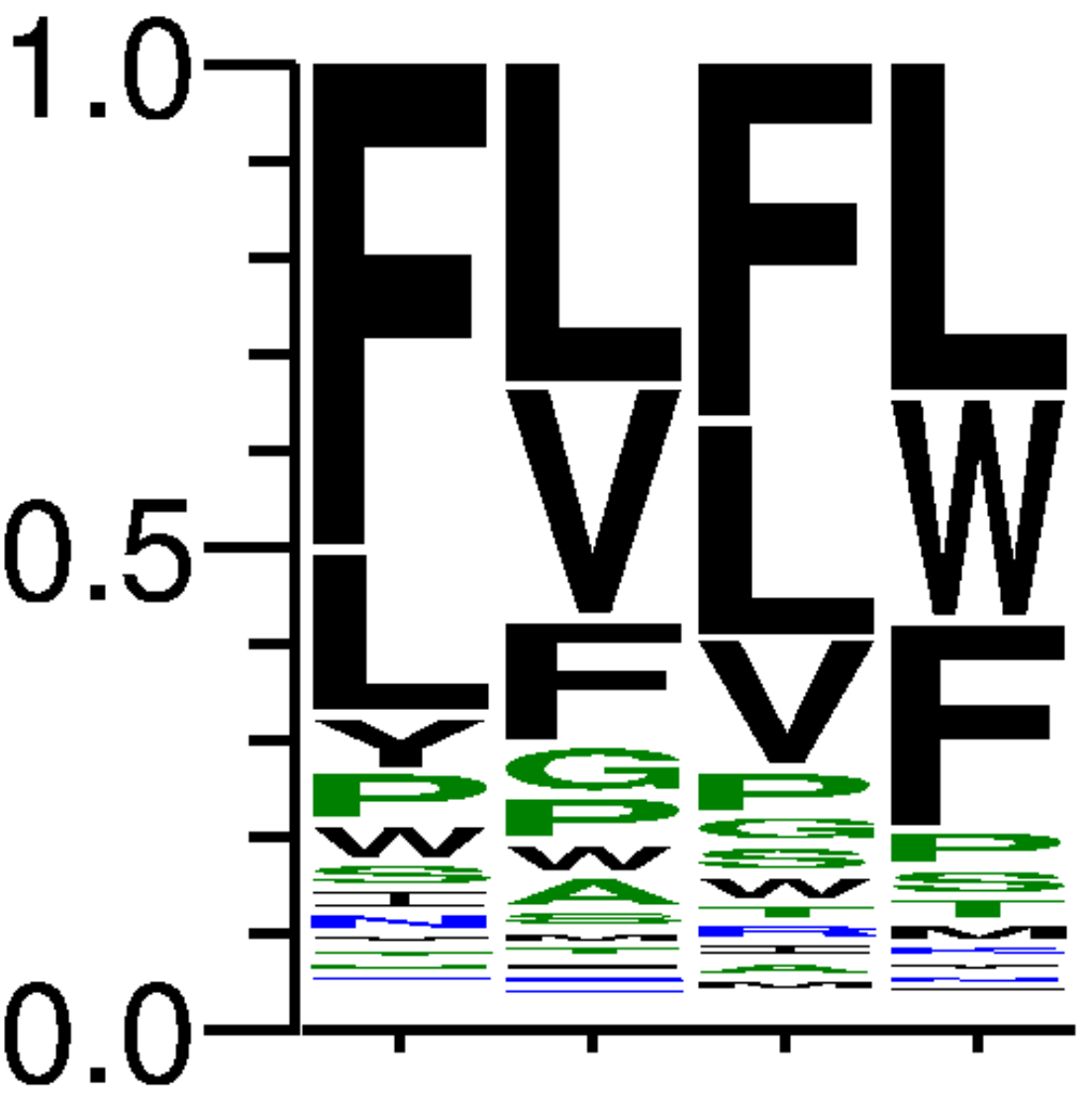 |
| 52 | CCC | 62703 | 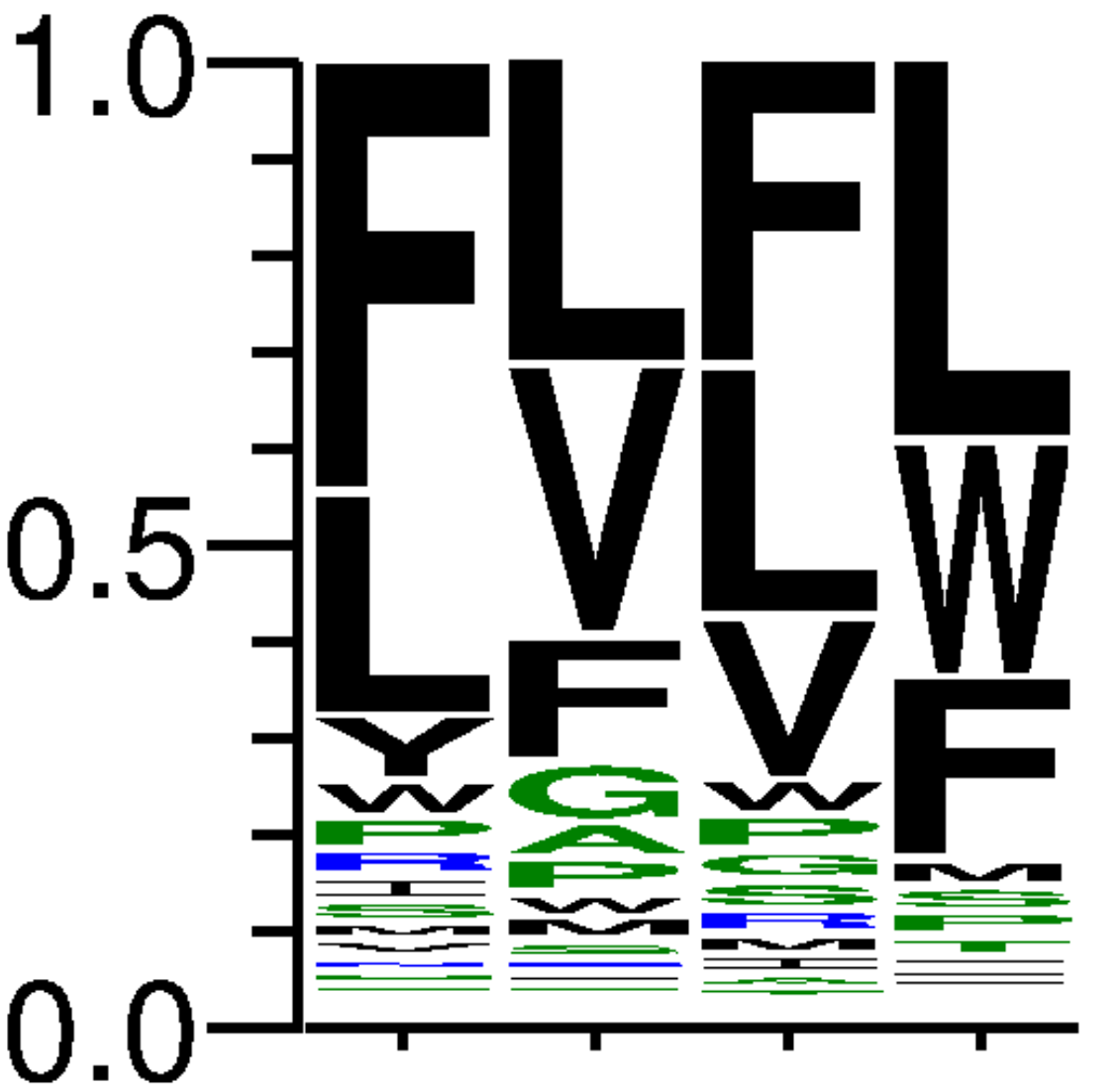 |
| 53 | CGG | 65713 | 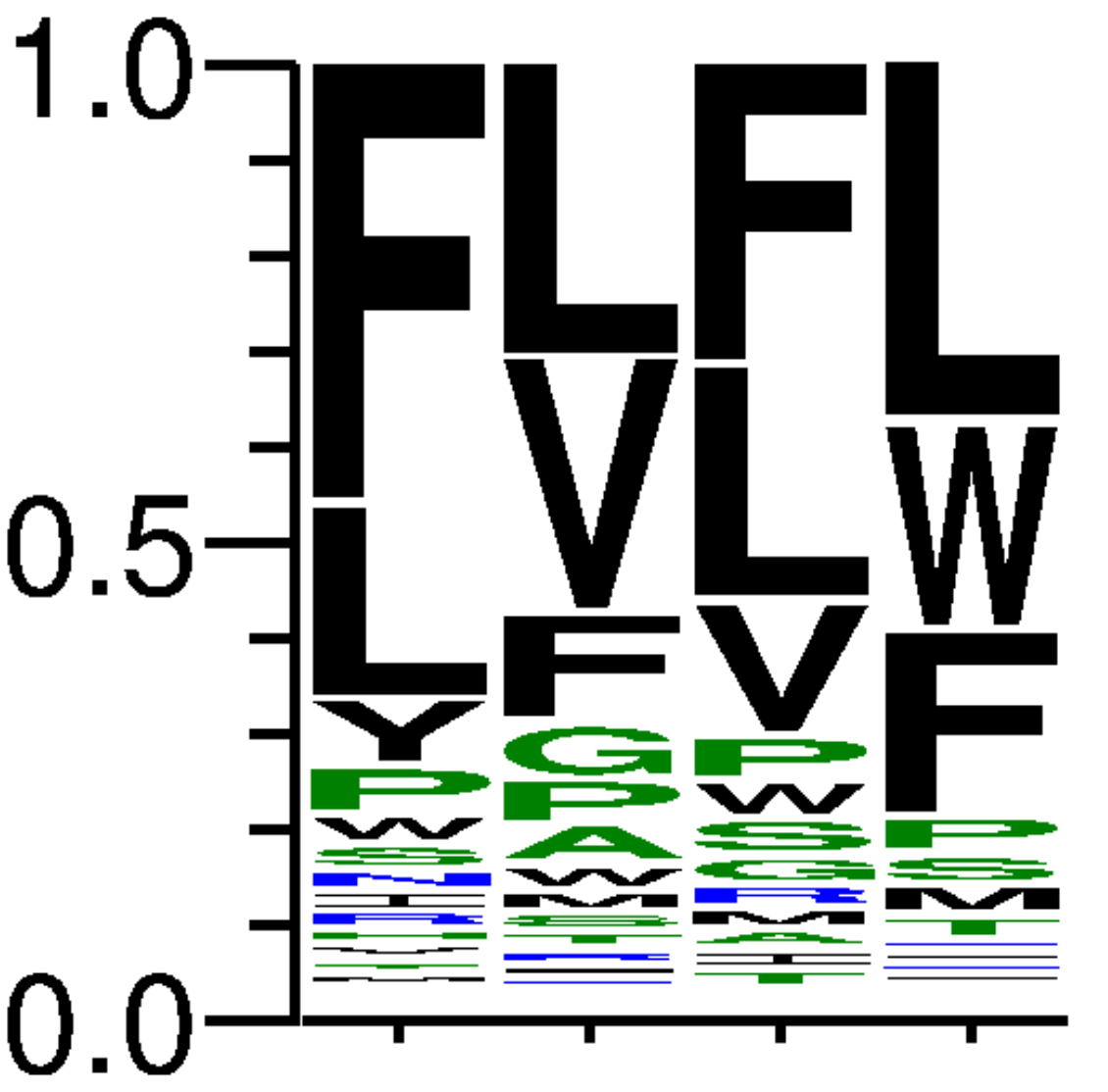 |
| 54 | CAG | 46101 | 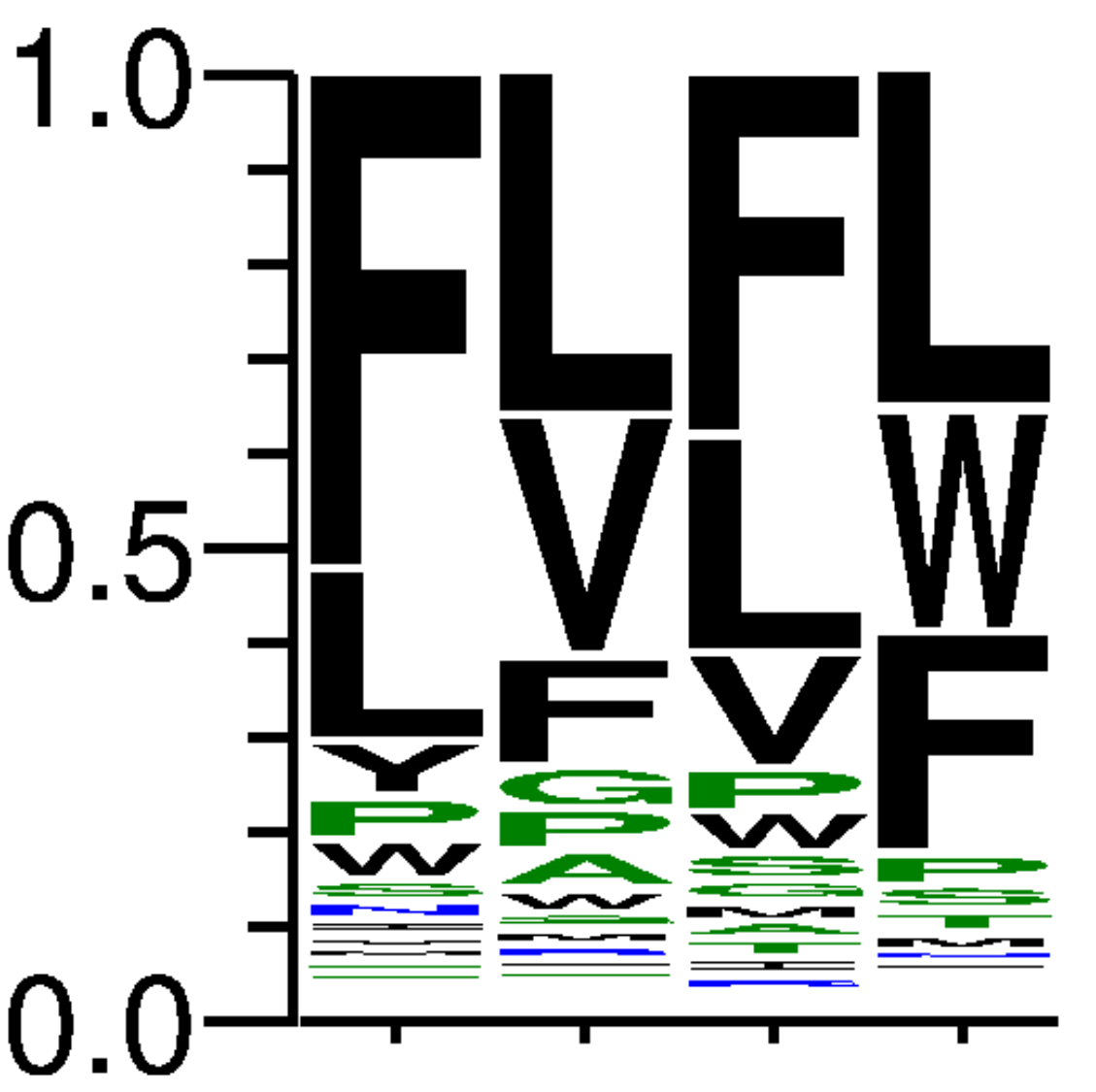 |
| 55 | CTG | 233659 | 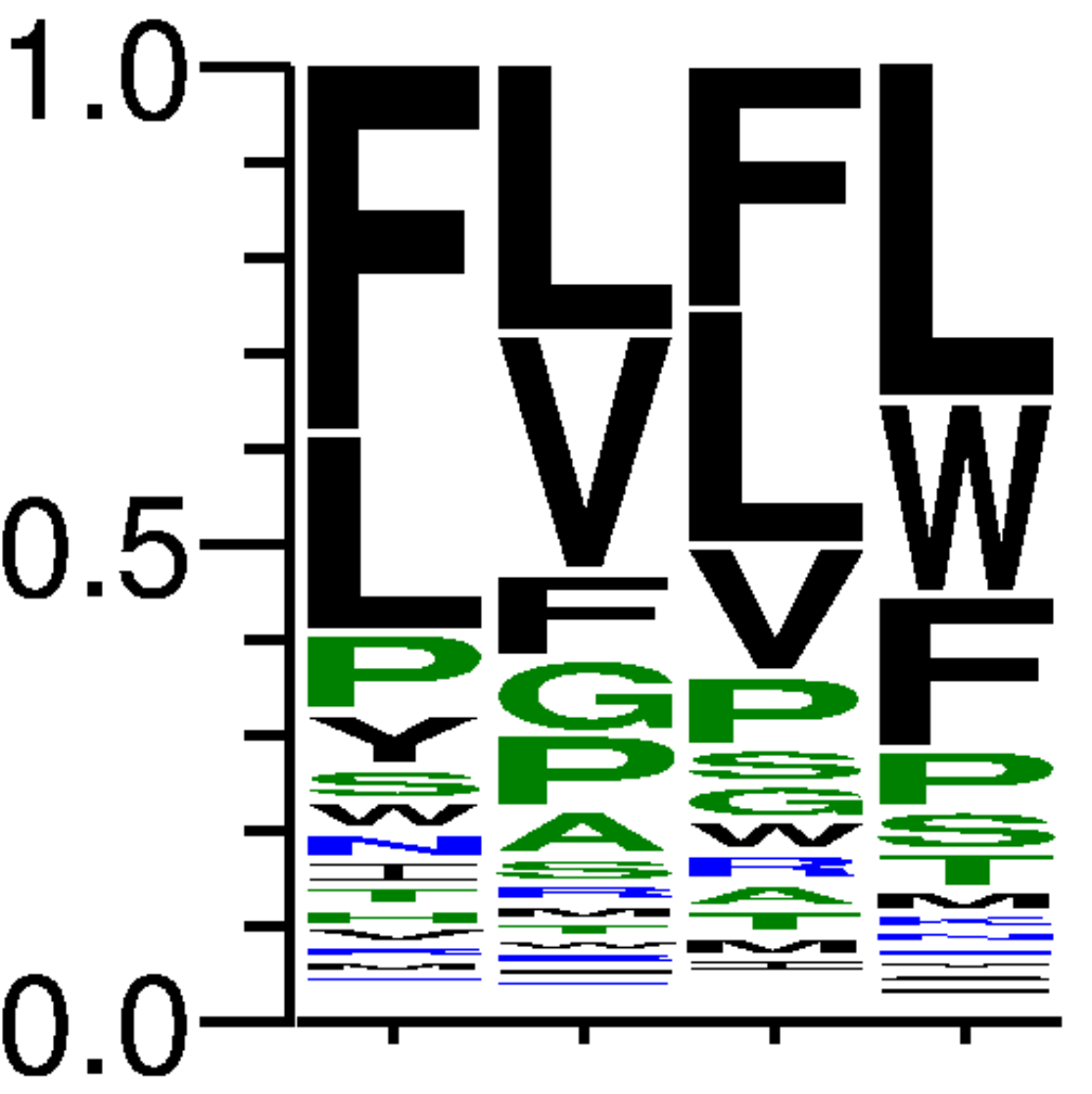 |
| 56 | CGA | 159945 | 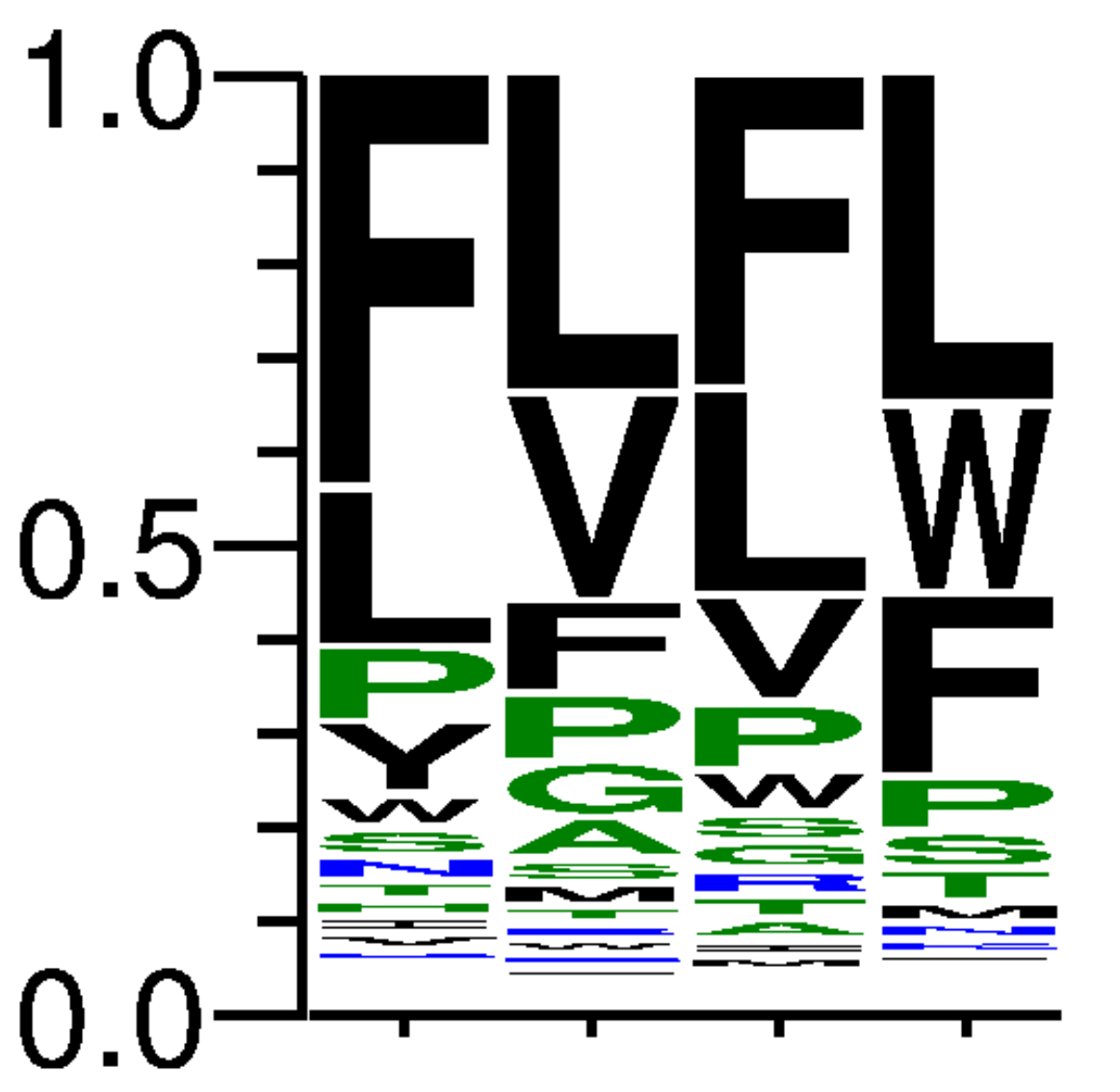 |
| 57 | CAA | 146246 | 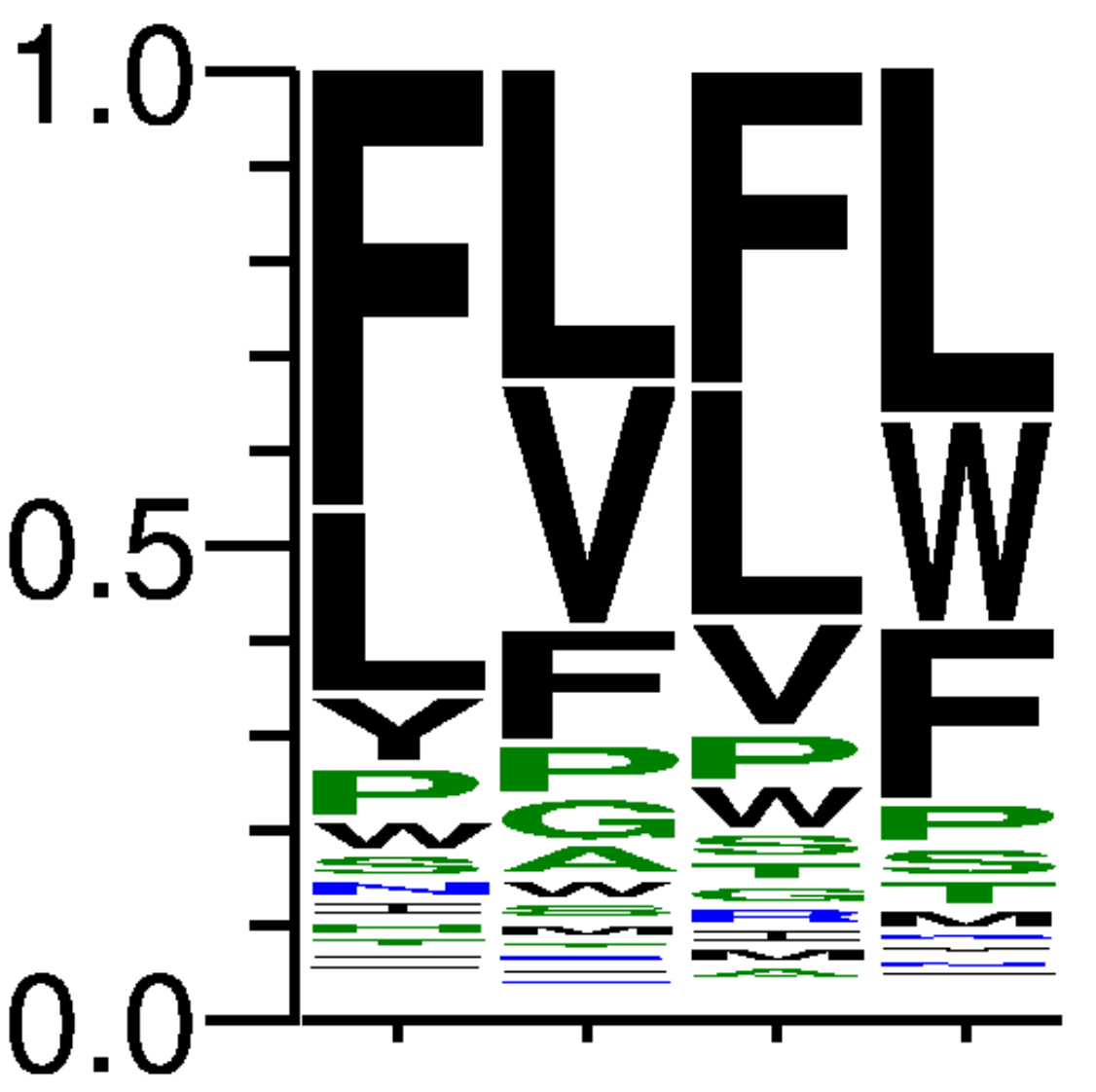 |
| 58 | CTA | 63761 | 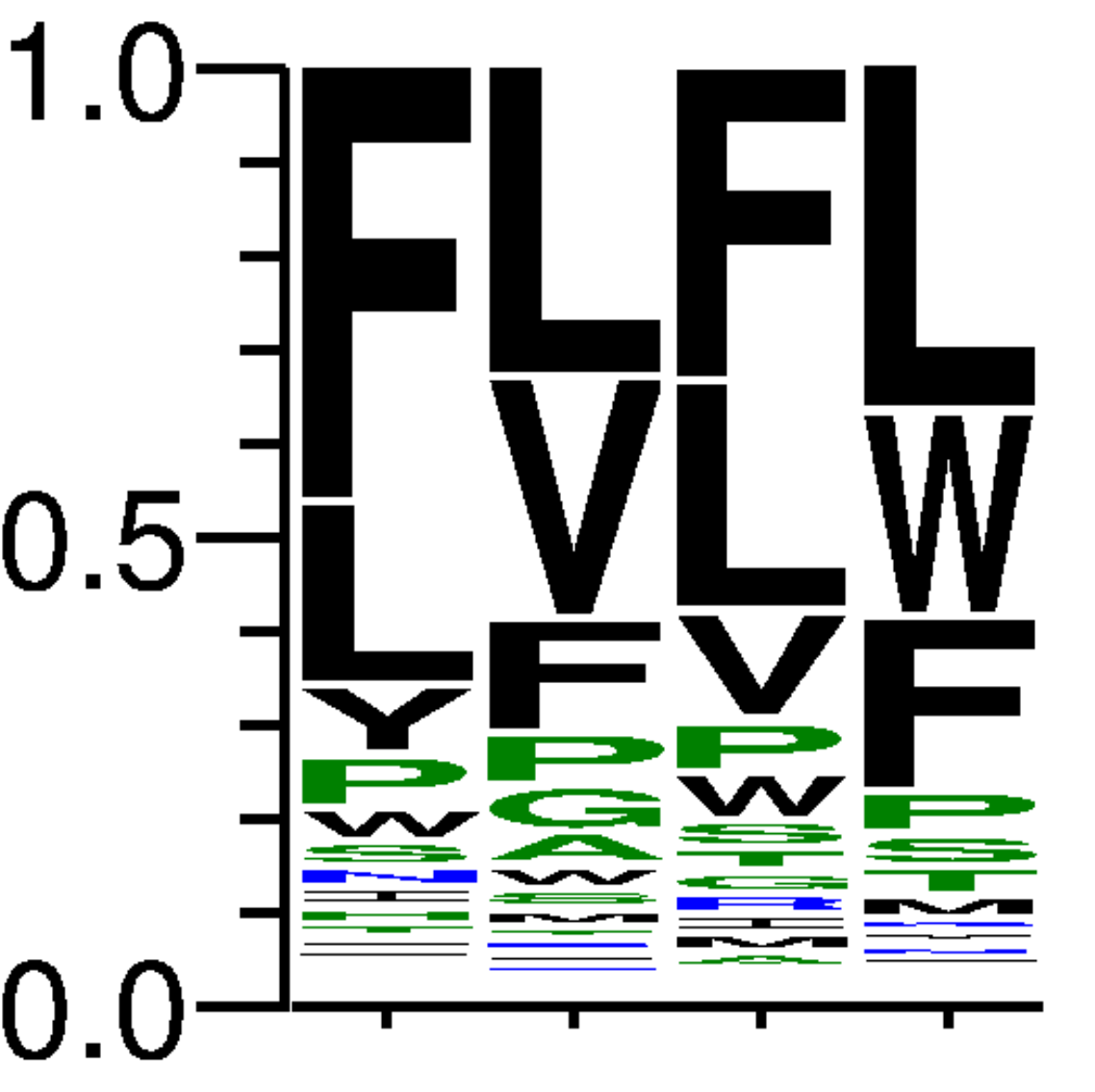 |
| 59 | CGT | 52372 | 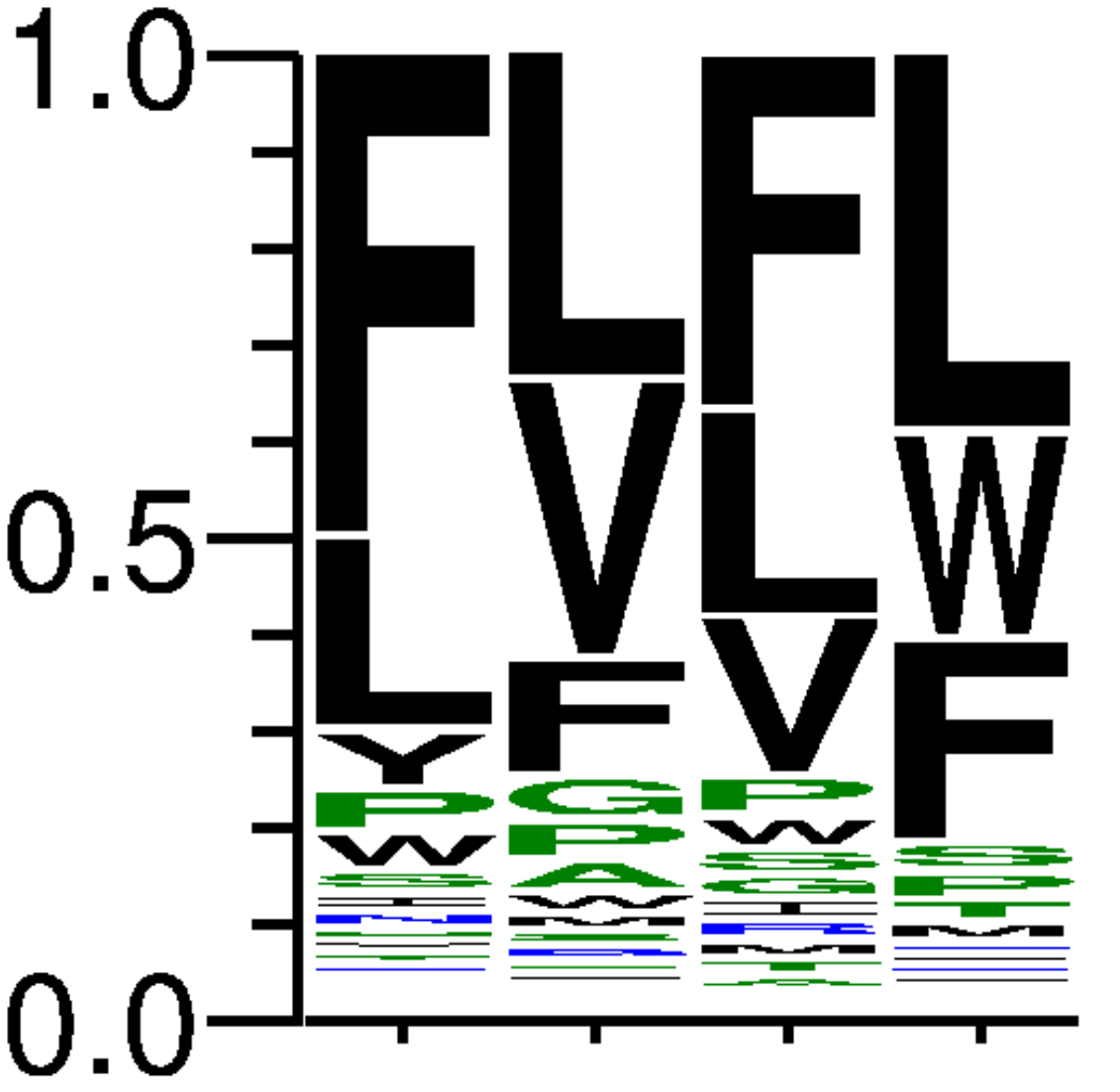 |
| 60 | CAT | 119039 | 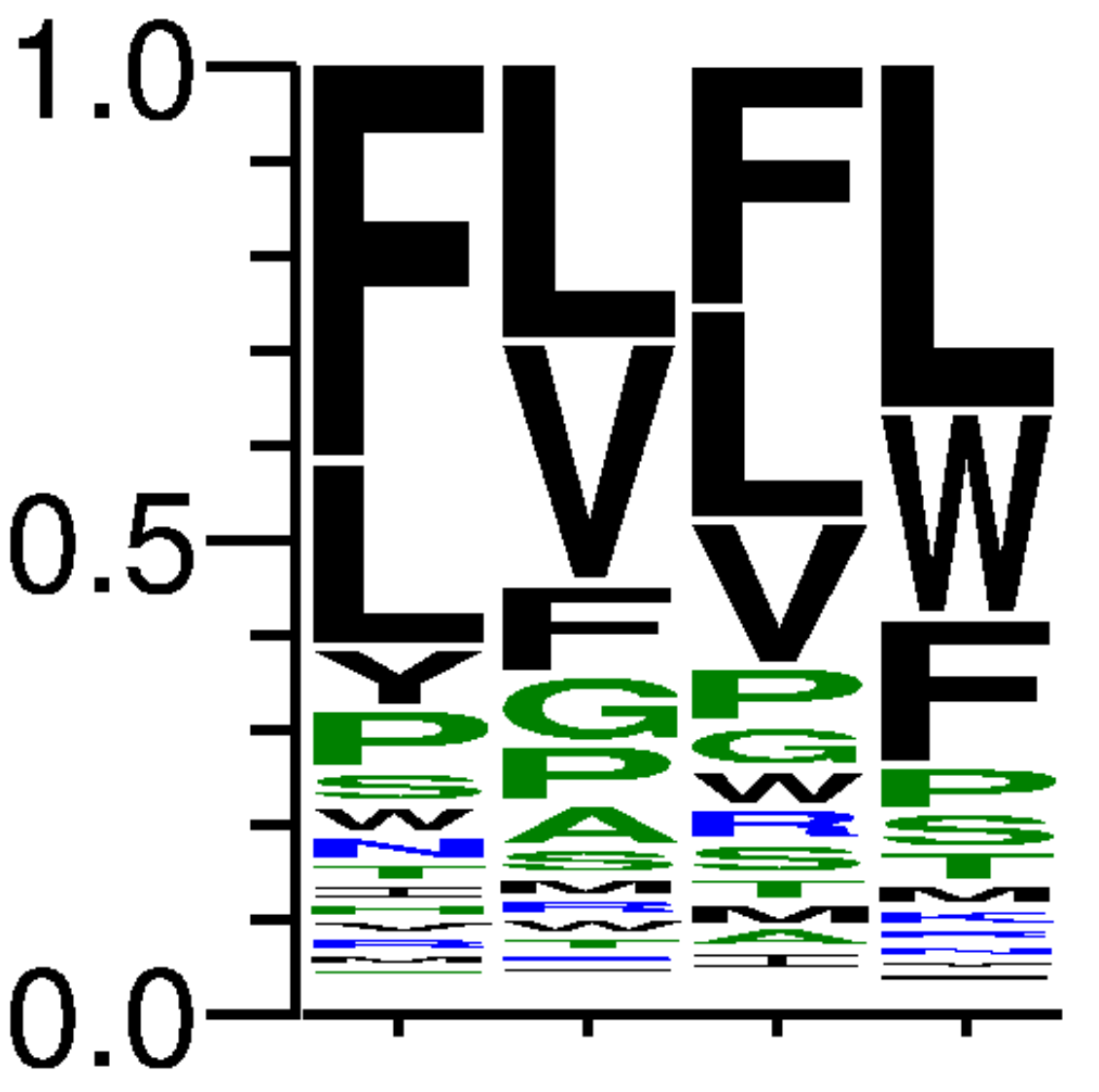 |
| 61 | CTT | 42718 | 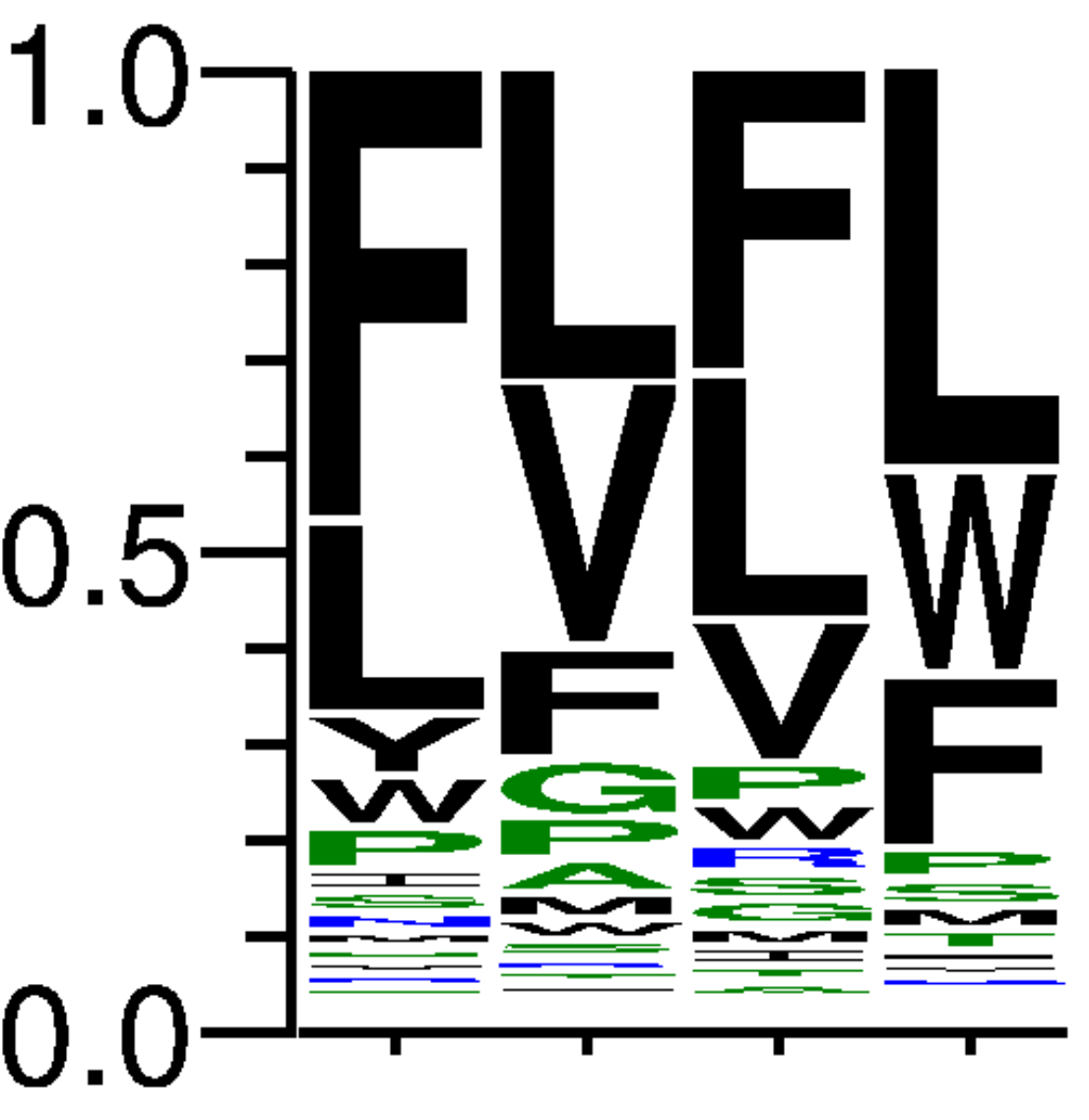 |
| 62 | CGC | 44639 | 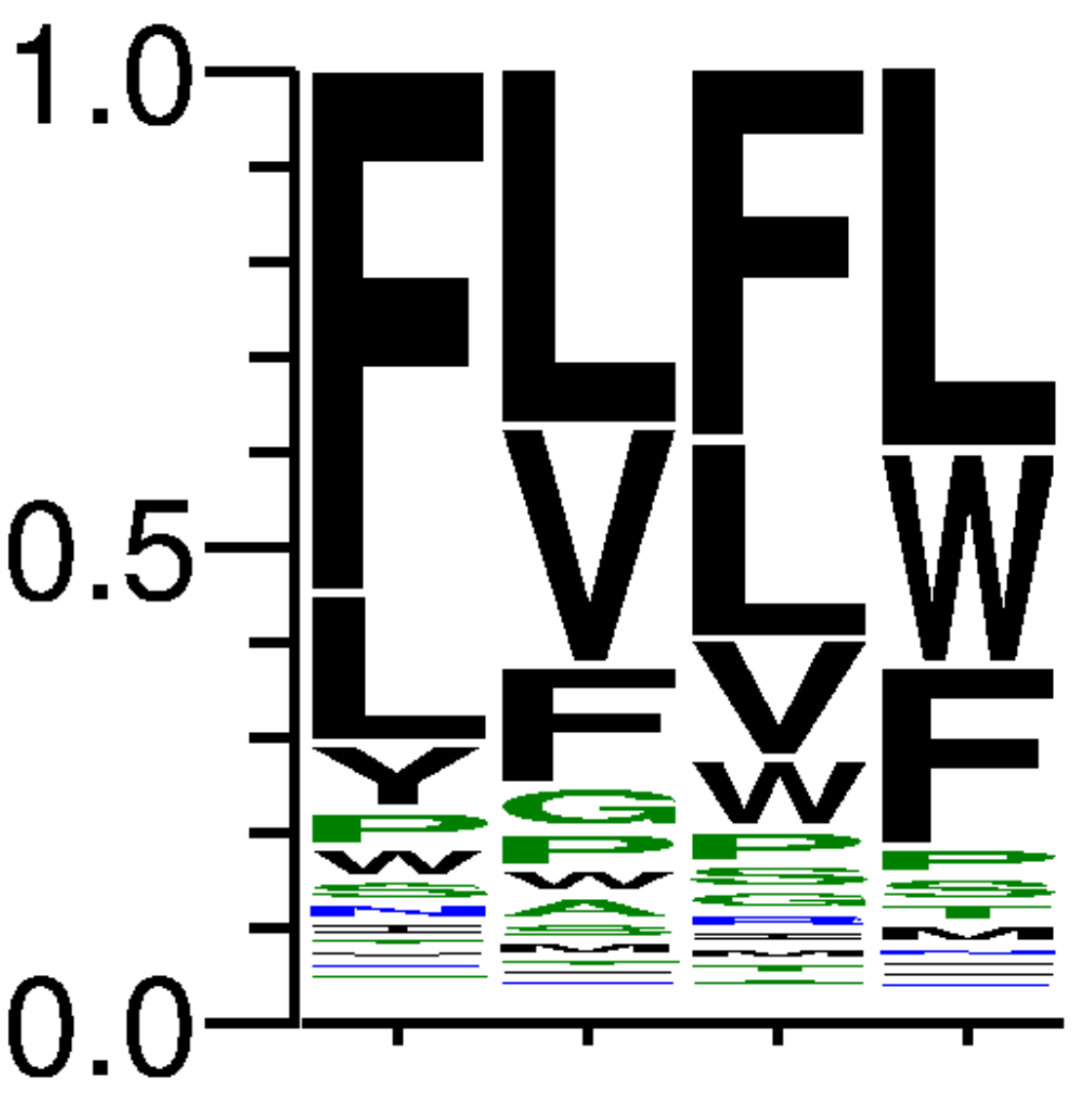 |
| 63 | CAC | 39525 | 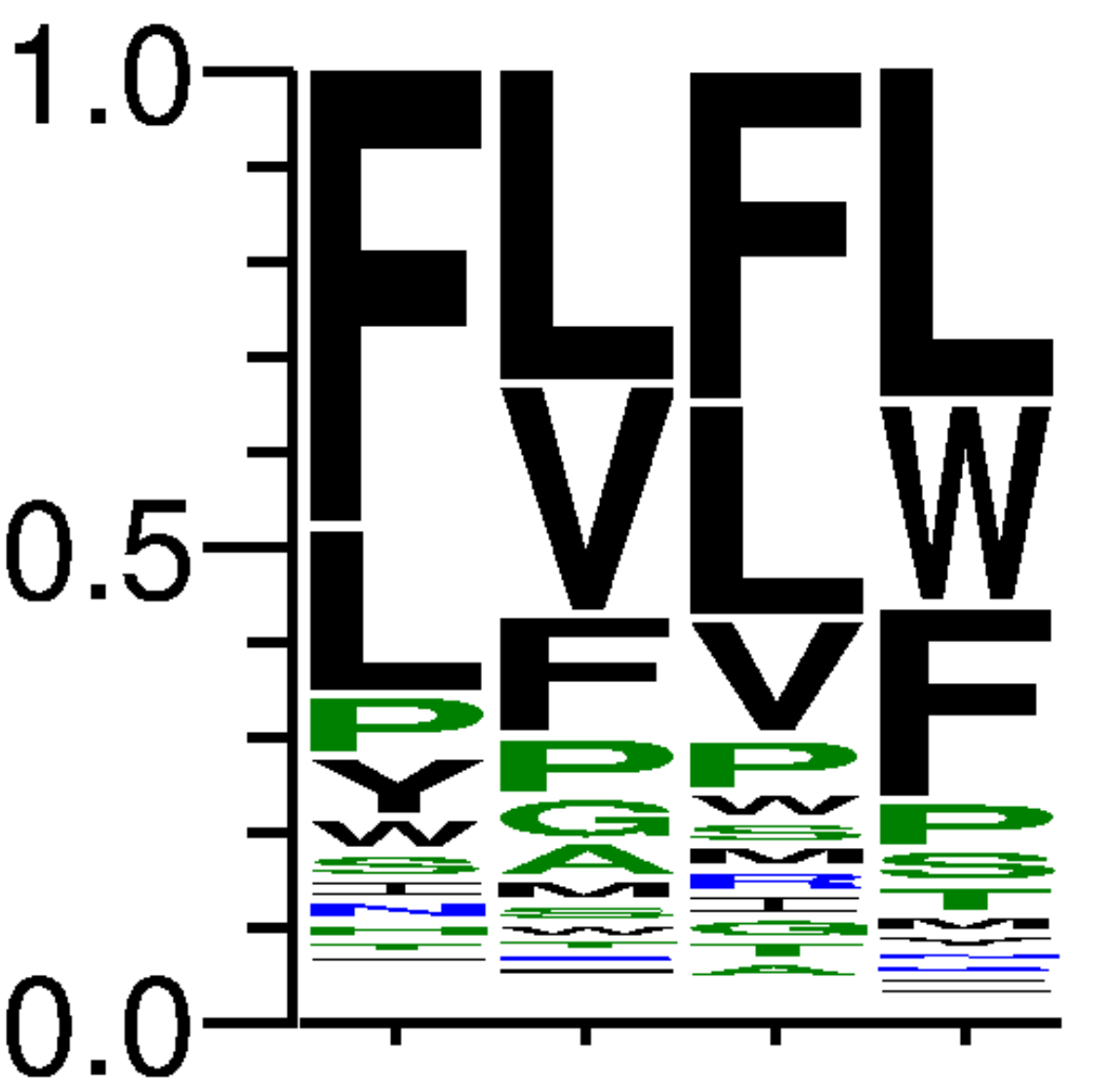 |
| 64 | CTC | 44527 | 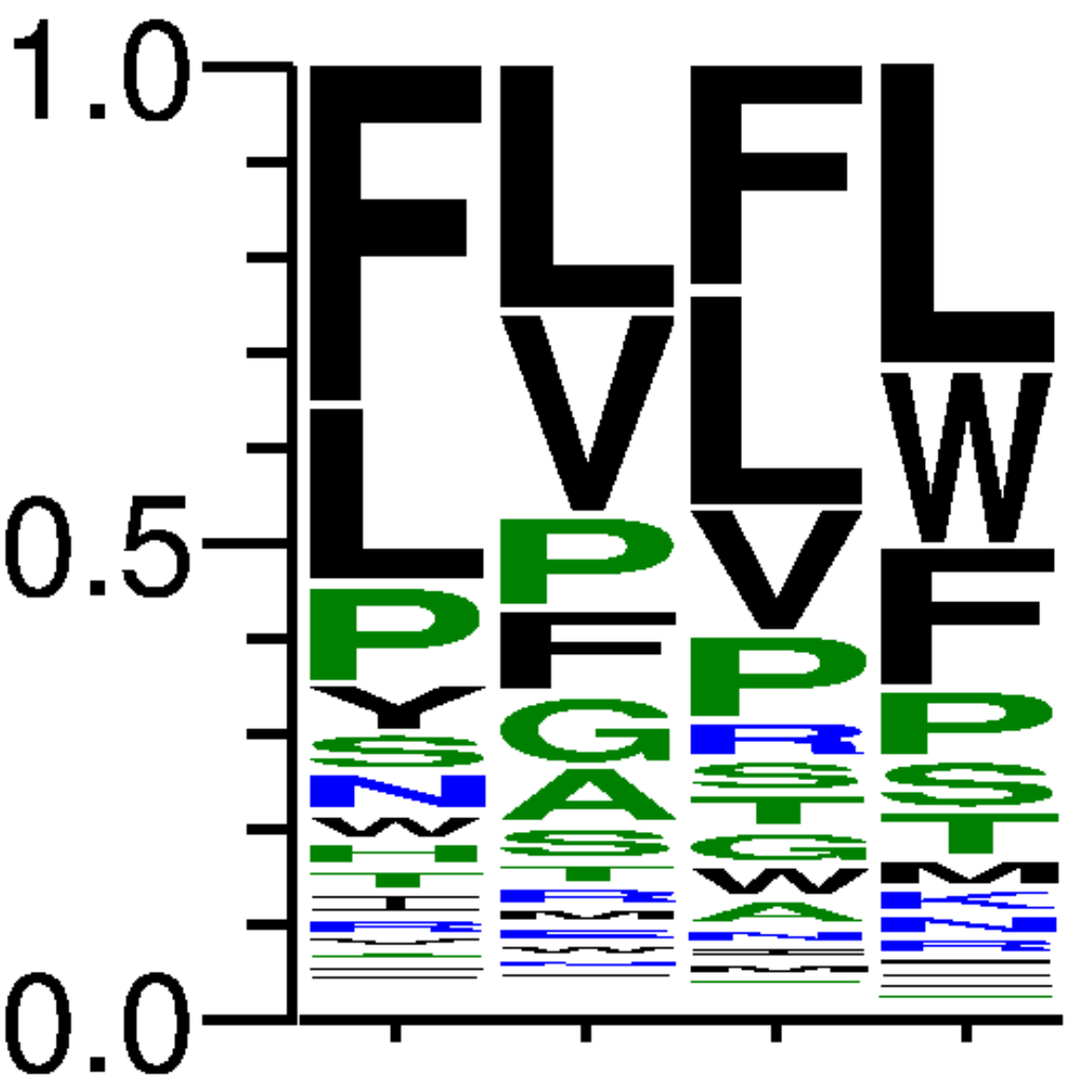 |

**Table S2**. Sequencing results of selected variants from the biopanning of the T7ZfQQR expressing the L5 and L6 library. The frequency logo was generated using all extracted amino acid residue sequences, where consecutive columns represent the frequencies obtained for randomized positions in the linkers.

| **Sample name** | **Nucleotide sequence used for selection** | **Number of reads passing filters** | **Frequency logo of amino acid residues from randomized region** |
| --- | --- | --- | --- |
| L5 | GGGGAAGAA | 41490 | See Figure 2B |
| L6 | GGGGAAGAA | 27850 | See Figure 2C |
